# Supplementary material for: Preparing Interns as Teachers: Teaching Fourth-Year Medical Students the Tenets of the One-Minute Preceptor Model
Source: MedEdPORTAL. 2023 Dec 26;19:11371. doi: 10.15766/mep_2374-8265.11371 (PMC10749993; doi:10.15766/mep_2374-8265.11371)
Supplement: Supplementary file 1 — Intern-as-Teacher Didactic.pptxCommitment and Justification Cases.docxTeach a General Rule Cases.docxFeedback Cases.docxFull OMP Practice Cases.docxOSTE Case.docxOSTE Rubric.docxPre-Post Evaluation.docxFacilitator Guide.docx [file mep_2374-8265.11371-s001.zip › B. Commitment and Justification Cases.docx]

Appendix B: Commitment and Justification Cases

## Instructions:

- Time:
  - 25 minutes for slides and practice
    - 10 minutes for slides
    - 15 minutes for practice
- Use these cases with Slide 16 pulled up
  - There are 5 cases for each specialty (Medicine, Pediatrics, Surgery)
  - Will give one packet of 5 cases to each group
- Each student will take one case
  - The student will read their case verbatim to one other member of the group
  - The person listening to the case will be the ‘intern’ and ask the person reading the case to commit to something and then ask them to justify that commitment
  - Should only be one commitment and one justification per case
  - Every member of the group will play both roles

## Internal Medicine Commitment/Justification Cases

**Case 1**

History of Present Illness:

- Andrea Curtis is a 21-year-old woman presenting with headache and fever that started last night. Her temperature at home was 101.5. Her headache is over entire head and worse with light. She notes neck pain and stiffness. Her roommate was sick last week as well but not sure what sick with.

She has no past medical or surgical history. She takes no medications. She does not smoke, drink or use illicit drugs.

On exam:

- Vital signs: Temperature 101, HR 115, BP 115/67, RR 18, SpO2 100% on Room air
- General: appears uncomfortable in bed
- Neck: nuchal rigidity noted
- Cardiovascular: tachycardic, no murmurs
- Neurological: awake, sleepy, answers questions appropriately

Labs:

- WBC 25 (92% PMNs), Hgb 13, plt 275. Rest of labs normal

LP:

- WBC 1550 (85% PMNs), Protein 150, Glucose 20

| Diagnosis | |
| --- | --- |
| What is the diagnosis?  What do you think is going on? | Bacterial meningitis |
| How would you justify your diagnosis? | Headache, fever, nuchal rigidity, and being a little sleepy  LP results show high WBC count and low glucose, which is consistent with bacterial meningitis. |
| Evaluation/Testing | |
| Any tests you would do? | Send PCR (meningitis/encephalitis panel) or culture on the CSF |
| How worried are you about this patient? | Her mental status is concerning but is still awake. I am worried about this patient and she will need close monitoring. |
| Treatment | |
| How would you like to treat him?  What antibiotics would you start? | Vancomycin and ceftriaxone. |

**Case 2**

History of Present Illness:

- Katrina Martin is an 18-year-old woman presenting with acute onset of RLQ pain. She noticed pain started 2 hours ago and has been worsening. She is having nausea and vomited twice. Subjective fever and some vaginal spotting after pain started. No chills, vaginal discharge, constipation or diarrhea.

No past medical or surgical history. She does not take any medications. No family history of chronic conditions.

Social:

- She does not smoke, drink or use illicit drugs. She is currently sexually active with her boyfriend. They do not use protection. She has inconsistent periods and her last period was 6 weeks ago, which is not atypical for her.

On exam:

- Vital signs: Temperature 99.9, HR 110, BP 100/60, RR 18, SpO2 100% on Room air
- General: appears uncomfortable in bed
- Abdominal: tender to palpation in RLQ, rebound and voluntary guarding present
- Rest of exam is normal

| Diagnosis | |
| --- | --- |
| What is the diagnosis?  What do you think is going on? | Ectopic pregnancy |
| How would you justify your diagnosis? | Acute onset of RLQ pain in a sexually active woman with last menstrual period 6 weeks ago and vaginal bleeding |
| Evaluation/Testing | |
| Any tests you would do? | Pelvic ultrasound  Pregnancy test |
| How worried are you about this patient? | She is stable for now, but if she has a ruptured ectopic, we will need to monitor her very closely |
| Treatment | |
| How would you like to treat her? | If she has an ectopic pregnancy, we will need to treat her accordingly either with medications or surgery depending on the ultrasound findings. |

**Case 3**

History of Present Illness:

- Jimmy Caldwell is a 75-year-old man presenting with rectal bleeding. Bleeding started 2 days ago. It is not associated with abdominal or rectal pain. His stools have been loose for the last 2 days but most of time he is constipated. Blood is bright red and coating the stool.

Past Medical and Surgical History: HTN and chronic constipation

Medications: Lisinopril and docusate as needed for constipation

Social History negative for smoking, drinking or use illicit drugs

On exam:

- Vital signs: Temperature 98.5, HR 100, BP 128/74, RR 18, SpO2 100% on Room air
- General: no acute distress
- Abdominal: soft, non-tender, non-distended
- Rectal: blood in rectal vault, two external hemorrhoids noted
- Everything else was normal

| Diagnosis | |
| --- | --- |
| What is the diagnosis?  What do you think is going on? | Diverticulosis |
| How would you justify your diagnosis? | Painless rectal bleeding in an older adult with a normal abdominal exam and vitals |
| Evaluation/Testing | |
| Any tests you would do? | Check a CBC and consider colonoscopy if the bleeding does not stop.  (If the bleeding doesn’t stop, he may need an intervention to stop the bleeding) |
| How worried are you about this patient? | Not worried at this point, but if bleeding continues, will need to monitor BP |
| Treatment | |
| How would you like to treat him? | Supportive care at this time (because he is stable) |

**Case 4**

History of Present Illness:

- Stephen Webster is a 55-year-old man presenting with chest pain to the Emergency Department. The chest pain started 30 minutes ago after climbing a flight of stairs at home. The pain is substernal and pressure-like, and it was 9/10 when it started. Resting and nitro given by EMS made it better, and the pain is now a 6/10. Moving made the pain worse. He has never had anything happen like this before.

Past Medical History: High blood pressure for 10 years and he takes amlodipine

No surgical history

Social History: He smokes 1ppd for 35 years. He does not drink alcohol or use illicit drugs. He is a truck driver and reports eating a lot of fast food.

Review of systems is otherwise negative except for feeling a little short of breath since this episode started but none before. Feeling flushed with this but not before it started. Otherwise negative.

On exam:

- Vital signs: Temperature 98.5, HR 110, BP 145/76, RR 18, SpO2 100% on Room air
- General: appears uncomfortable in bed, mildly diaphoretic
- Cardiovascular: regular rhythm, tachycardic, no murmurs/rubs/gallops
- Respiratory: clear to auscultation bilaterally, no wheezes/crackles
- Everything else was normal

His labs were normal except for an initial troponin of 150 (normal <14). The 2 hour value is still pending

His EKG showed sinus tachycardia but some new T wave inversions

| Diagnosis | |
| --- | --- |
| What is the diagnosis?  What do you think is going on? | Acute coronary syndrome |
| How would you justify your diagnosis? | 55 year old man with substernal chest pain that occurred with exertion and was relieved by nitro. He has risk factors like smoking and HTN. His initial troponin was elevated and his EKG shows signs of ischemia with T wave inversions that are new. |
| Evaluation/Testing | |
| Any tests you would do? | Will need to repeat the troponin in 2 hours with another EKG. (We need to see the trend of both as will determine if has ongoing heart damage)  Would like to discuss with cardiology as will likely need a stress test or catheterization |
| How worried are you about this patient? | Worried – could progress and need intervention |
| Treatment | |
| How would you like to treat him? | Continue nitroglycerin and call cardiology  (Nitroglycerin will help with the chest pain and cardiology can determine the timing of the stress test) |

**Case 5**

History of Present Illness:

- Faye Howard is a 68-year-old woman with acute onset of back pain being seen in the primary care clinic. The pain started when she was moving boxes in her garage, not lifting them, but moving them on the floor. The pain is in her mid-back and is an 8/10. It is constant with pain worsening with movement. She gets some relief with ibuprofen at home. Denies pain like this before or any falls or other injuries.

Past Medical History

- Breast cancer 2 year ago, ER+. Had a mastectomy and chest wall radiation. No chemotherapy. Has been on anastrozole since then

Social History:

- Walks everyday with her sister but her back pain has prevented her from doing this last 2 days
- Doesn’t smoke or drink alcohol or use illicit drugs

On exam:

- Vital signs: Temperature 98.5, HR 88, BP 135/76, RR 18, SpO2 100% on RA
- General: resting comfortably in bed, but pain with movements
- Heart and lungs were fine
- Musculoskeletal: back is tender to palpation over the T10 spinous process, no muscular tenderness noted back, no radicular pain with leg raise
- Neurological: normal
- Everything else was normal

| Diagnosis | |
| --- | --- |
| What is the diagnosis?  What do you think is going on? | Vertebral fracture possibly from new metastatic breast cancer |
| How would you justify your diagnosis? | She has acute back pain that is tender to palpation at T10. Her past medical history of breast cancer makes me concerned she might have metastatic disease to her spine, predisposing her to a fracture. |
| Evaluation/Testing | |
| Any tests you would do? | MRI of her thoracic spine to assess for fracture |
| How worried are you about this patient? | She does not have any neurological compromise on exam, so I am not worried right now |
| Treatment | |
| How would you like to treat her? | Start with oral acetaminophen for pain. Could add more pain medicines if not controlled. |

# Pediatrics Commitment/Justification Cases

**Case 1**

History of Present Illness:

- Andrea Curtis is a 10-year-old girl presenting with headache and fever that started last night. Her temperature at home was 101.5. Her headache is over entire head and worse with light. She notes neck pain and stiffness. Her sister was sick last week as well but not sure what sick with.

She has no past medical or surgical history. She takes no medications. She does not smoke, drink or use illicit drugs.

On exam:

- Vital signs: Temperature 101, HR 115, BP 115/67, RR 18, SpO2 100% on Room air
- General: appears uncomfortable in bed
- Neck: nuchal rigidity noted
- Cardiovascular: tachycardic, no murmurs
- Neurological: awake, sleepy, answers questions appropriately

Labs:

- WBC 25 (92% PMNs), Hgb 13, plt 275. Rest of labs normal

LP:

- WBC 1550 (85% PMNs), Protein 150, Glucose 20

| Diagnosis | |
| --- | --- |
| What is the diagnosis?  What do you think is going on? | Bacterial meningitis |
| How would you justify your diagnosis? | Headache, fever, nuchal rigidity, and being a little sleepy  LP results show high WBC count and low glucose, which is consistent with bacterial meningitis. |
| Evaluation/Testing | |
| Any tests you would do? | Send PCR (Biofire) or culture on the CSF (to find the specific bacteria involved) |
| How worried are you about this patient? | Her mental status is concerning but is still awake. I am worried about this patient and she will need close monitoring. |
| Treatment | |
| How would you like to treat her? or  What antibiotics would you start?  Why did you choose these antibiotics? | I would start vancomycin (*pronounced: van-co-my-o-sin*) and ceftriaxone (*pronounced: sef-try-ax-own*).  I think these are the correct antibiotics for bacterial meningitis |

**Case 2**

History of Present Illness:

- Katrina Martin is a 14-year-old girl presenting with acute onset of RLQ pain. She noticed pain started 2 hours ago and has been worsening. She is having nausea and vomited twice. Subjective fever. No chills, vaginal bleeding or discharge, constipation or diarrhea.

No past medical or surgical history. She does not take any medications. No family history of chronic conditions.

Social:

- She does not smoke, drink or use illicit drugs. She is not currently sexually active. She had her last menstrual period 2 weeks ago.

On exam:

- Vital signs: Temperature 99.9, HR 110, BP 100/60, RR 18, SpO2 100% on Room air
- General: appears uncomfortable in bed
- Abdominal: tender to palpation in RLQ, rebound and voluntary guarding present
- Rest of exam is normal

| Diagnosis | |
| --- | --- |
| What is the diagnosis?  What do you think is going on? | Appendicitis |
| How would you justify your diagnosis? | Young woman with acute onset right lower quadrant pain with nausea and vomiting. She is tender to palpation in the right lower quadrant with voluntary guarding |
| Evaluation/Testing | |
| Any tests you would do? | Abdominal ultrasound (to evaluate the appendix)  Ultrasound avoids unnecessary radiation in kids |
| How worried are you about this patient? | I am reassured that her abdominal exam does not show any peritoneal signs, but she will likely need urgent surgery |
| Treatment | |
| How would you like to treat her? | She will likely need surgery. No antibiotics right now. If her surgery is going to need to be delayed, then maybe later |

**Case 3**

History of Present Illness:

- Jody Henry is a 15-year-old girl who presented with right knee pain after a fall to the ED. She was playing in a soccer game when she planted her right foot, turned to go a different direction and thought she felt a ‘pop’ but wasn’t sure. The pain started immediately. It is stabbing in her right knee and has been a constant 9/10. Her knee has become more swollen in the 2 hours since her injury. She tried to walk off the field but felt ‘too unstable to walk’ and the pain was severe. She exercises regularly and has never had any other major injuries. No prior injury to her knees.

She has no past medical or surgical history. She takes no medications.

Social History: She does not drink or smoke or use illicit drugs. She is a high school student and soccer player.

On exam:

- Vital signs: Temperature 98.5, HR 85, BP 125/81, RR 12, SpO2 100% on RA
- Musculoskeletal: right knee is swollen and tender to palpation, anterior draw and Lachman are positive, left knee is normal
- Skin: no injury in skin overlying the right knee but skin is mildly red and swollen, no rashes noted
- Everything else was normal

Her labs are normal

Xray of her knee didn’t show a fracture

| Diagnosis | |
| --- | --- |
| What is the diagnosis?  What do you think is going on? | ACL tear |
| How would you justify your diagnosis? | Young athlete with a deceleration injury and immediate pain and swelling. She was unable to bear weight. Her anterior drawer and Lachman’s signs are both positive, which makes ACL tear most likely |
| Evaluation/Testing | |
| Any tests you would do? | MRI knee (for surgical planning) |
| How worried are you about this patient? | She is stable and her pain is controlled after acetaminophen given in ER. I am not worried at this time |
| Treatment | |
| How would you like to treat him? | Discuss with orthopedic surgery team about timing of her ACL repair. She will need surgery to be able to walk or play sports again |

**Case 4**

History of Present Illness:

- Kristen Simpson is a 4-day-old who presents to clinic with jaundice. She developed jaundice at 48-hours that has been progressing since that time. She has been acting normally at home. Weight is nearly back to birth weight.

She was born by a normal vaginal delivery. Normal prenatal screen. Has been feeding well, but her mom notes that breastfeeding has been harder than expected. She is stooling and peeing normally.

On exam:

- Vital signs: Temperature 98.5, HR 120, BP 90/45, RR 12, SpO2 100% on RA
- Skin: jaundice noted throughout. No other rashes
- Cardiovascular: normal
- Abdomen: normal

Her bilirubin is 5.

| Diagnosis | |
| --- | --- |
| What is the diagnosis?  What do you think is going on? | Physiologic jaundice |
| How would you justify your diagnosis? | 4-day old with jaundice that started after 36-hours. She has been eating, stooling and voiding normally. She is acting normally and her bilirubin is 5. |
| Evaluation/Testing | |
| Any tests you would do? | None right now (as long as she continues to eat, pee, and poop). Would recommend getting hemolysis labs if not improving |
| How worried are you about this patient? | No as her exam and mental status are good  (If she were to get more sleepy or stop eating/peeing/pooping, then I would be more worried) |
| Treatment | |
| How would you like to treat her? | Encourage more feeding (breast or formula) for 2 days and return. If she eats more, her symptoms should improve. |

**Case 5**

History of Present Illness:

- Bobby Newton is an 8-year-old who presents to the ER with progressive shortness of air. He had nasal congestions and sore throat for the 2 days prior and has a nonproductive cough for the last several hours. He is having chest pain that started today. His mother tried albuterol nebulizers twice at home without improvement.

Past medical history of asthma. Uses albuterol inhaler at home.

On exam:

- Vital signs: Temperature 98.5, HR 130, BP 100/65, RR 40, SpO2 88% on RA
- General: appears drowsy but is arousable
- Cardiovascular: tachycardic, no murmurs
- Pulmonary: poor air movement, with expiratory wheezes, using accessory muscles to breathe

| Diagnosis | |
| --- | --- |
| What is the diagnosis?  What do you think is going on? | Asthma exacerbation |
| How would you justify your diagnosis? | 8-year-old with history of asthma presenting with progressive shortness of breath after likely upper respiratory infection. On exam, has poor air movement, expiratory wheezes, and is hypoxic |
| Evaluation/Testing | |
| Any tests you would do? | Chest X-ray (to evaluate for possible pneumonia) |
| How worried are you about this patient? | Yes, he is drowsy and hypoxic. May have impending respiratory failure |
| Treatment | |
| How would you like to treat him? | Start continuous albuterol and admit to the PICU with close monitoring. Given that he is drowsy and hypoxic, he needs close monitoring and treatment. |

## Surgery Commitment/Justification Cases

**Case 1**

History of Present Illness:

- Andrew Curtis is a 77-year-old man presenting with 35-pound weight loss over the last 5 months. He has been unable to eat much during that time as every time he tries to eat, he develops diffuse, severe abdominal pain. He has tried to eat smaller meals to improve the pain with little help.

He has a past medical history of HTN and is on lisinopril. He smokes 1ppd for last 55 years. He drinks 1 glass of bourbon each night. No illicit drugs.

On exam:

- Vital signs: Temperature 98, HR 85, BP 115/67, RR 18, SpO2 100% on Room air
- General: resting comfortably
- Cardiovascular: RRR, no murmurs/rubs/gallops, pulses in her extremities are trace
- Abdomen: soft, nontender, nondistended, possible bruit (*pronounced: brew-ey*)heard

Hemoccult is negative

| Diagnosis | |
| --- | --- |
| What is the diagnosis?  What do you think is going on? | Chronic mesenteric ischemia |
| How would you justify your diagnosis? | Older man with risk factors for vascular disease (smoking, HTN) who presented with weight loss and abdominal pain with eating. He has diminished peripheral pulses and a possible bruit on abdominal exam |
| Evaluation/Testing | |
| Any tests you would do? | CT-angiography of abdomen (to evaluate for narrowing of the blood vessels to the intestines) |
| How worried are you about this patient? | Not at this moment but she needs surgery soon |
| Treatment | |
| How would you like to treat him? | He will likely need revascularization for his mesenteric ischemia  (He will continue to have pain and possible intestine damage if the blood flow is not restored) |

**Case 2**

History of Present Illness:

- Katrina Martin is an 18-year-old woman presenting with acute onset of right lower quadrant pain. She noticed pain started 2 hours ago and has been worsening. She is having nausea and vomited twice. Subjective fever. No chills, vaginal bleeding or discharge, constipation or diarrhea.

No past medical or surgical history. She does not take any medications. No family history of chronic conditions.

Social:

- She does not smoke, drink or use illicit drugs. She is currently sexually active with her boyfriend. They do not use protection. She has inconsistent periods and her last period was 3 weeks ago, which is not atypical for her.

On exam:

- Vital signs: Temperature 99.9, HR 110, BP 100/60, RR 18, SpO2 100% on Room air
- General: appears uncomfortable in bed
- Abdominal: tender to palpation in RLQ, rebound and voluntary guarding present
- Rest of exam is normal

| Diagnosis | |
| --- | --- |
| What is the diagnosis?  What do you think is going on? | Appendicitis |
| How would you justify your diagnosis? | Young woman with acute onset right lower quadrant pain with nausea and vomiting. She is tender to palpation in the right lower quadrant with voluntary guarding |
| Evaluation/Testing | |
| Any tests you would do? | Abdominal CT scan (to evaluate the appendix) |
| How worried are you about this patient? | I am reassured that her abdominal exam does not show any peritoneal signs, but she will likely need urgent surgery |
| Treatment | |
| How would you like to treat her? | She will likely need surgery. No antibiotics right now. If her surgery is going to need to be delayed, then maybe later |

**Case 3**

History of Present Illness:

- Stephen Webster is a 55-year-old man who presented with chest pain after hitting the steering wheel column during a motor vehicle collision (MVC). Chest pain is constant and has not relieving factors. Pain a little worse with movement but not much. Never had pain like this before.

Past Medical History: High blood pressure for 10 years and he takes amlodipine

No surgical history

Social History: He does not smoke, drink alcohol or use illicit drugs. He is a truck driver

Review of systems is positive for chest pain, shortness of breath, and a feeling of unease.

On exam:

- Vital signs: Temperature 98.5, HR 110, BP in right arm is 175/95 and 150/82 in left arm, RR 18, SpO2 95% on Room air
- General: appears uncomfortable in bed, mildly diaphoretic
- Cardiovascular: regular rhythm, tachycardic, no murmurs/rubs/gallops
- Respiratory: clear to auscultation bilaterally, no wheezes/crackles
- MSK: chest is tender to palpation

His EKG showed sinus tachycardia but some new T wave inversions in anterior leads

| Diagnosis | |
| --- | --- |
| What is the diagnosis?  What do you think is going on? | Thoracic aortic injury |
| How would you justify your diagnosis? | Chest pain after hitting the steering column in a motor vehicle collision. He has asymmetric blood pressures in his arms. |
| Evaluation/Testing | |
| Any tests you would do? | CT-angiogram of his chest (to evaluate for possible aortic injury or aneurysm) |
| How worried are you about this patient? | I am worried. He might have injured his aorta and require surgery.  (Less likely a full rupture as his blood pressure is not low) |
| Treatment | |
| How would you like to treat him? | Monitor his blood pressure and follow up on his CT scan. Will likely need surgery soon.  (His CT scan will determine timing and type of surgery) |

**Case 4**

History of Present Illness:

- Faye Howard is a 68-year-old woman with acute onset of back pain being seen in the primary care clinic. The pain started when she was moving boxes in her garage, not lifting them, but moving them on the floor. The pain is in her mid-back and is an 8/10. It is constant with pain worsening with movement. She gets some relief with ibuprofen at home. Denies pain like this before or any falls or other injuries.

Past Medical History

- Breast cancer 2 year ago, ER+. Had a mastectomy and chest wall radiation. No chemotherapy. Has been on anastrozole since then

Social History:

- Walks everyday with her sister but her back pain has prevented her from doing this last 2 days
- Doesn’t smoke or drink alcohol or use illicit drugs

On exam:

- Vital signs: Temperature 98.5, HR 88, BP 135/76, RR 18, SpO2 100% on RA
- General: resting comfortably in bed, but pain with movements
- Heart and lungs were normal
- Musculoskeletal: back is tender to palpation over the T10 spinous process, no muscular tenderness noted back, no radicular pain with leg raise
- Everything else was normal

| Diagnosis | |
| --- | --- |
| What is the diagnosis?  What do you think is going on? | Vertebral fracture possibly from new metastatic breast cancer |
| How would you justify your diagnosis? | She has acute back pain that is tender to palpation at T10. Her past medical history of breast cancer makes me concerned she might have metastatic disease to her spine, predisposing her to a fracture. |
| Evaluation/Testing | |
| Any tests you would do? | MRI of her thoracic spine (to evaluate for fracture and possible metastasis to spine) |
| How worried are you about this patient? | She does not have any neurological compromise on exam, so I am not worried right now |
| Treatment | |
| How would you like to treat her? | Start with oral acetaminophen for pain. Could add more pain medicines if not controlled.  (Both are good for treating this type of pain) |

**Case 5**

History of Present Illness:

- Jody Henry is a 16-year-old woman who presented with right knee pain after a fall to the ED. She was playing in a soccer game when she planted her right foot, turned to go a different direction and thought she felt a ‘pop’ but wasn’t sure. The pain started immediately. It is stabbing in her right knee and has been a constant 9/10. Her knee has become more swollen in the 2 hours since her injury. She tried to walk off the field but felt ‘too unstable to walk’ and the pain was severe. She exercises regularly and has never had any other major injuries. No prior injury to her knees.

She has no past medical or surgical history. She takes no medications.

Social History: She does not drink or smoke or use illicit drugs. She is a high school student and soccer player.

On exam:

- Vital signs: Temperature 98.5, HR 85, BP 125/81, RR 12, SpO2 100% on RA
- Musculoskeletal: right knee is swollen and tender to palpation, anterior draw and Lachman are positive, left knee is normal
- Skin: no injury in skin overlying the right knee but skin is mildly red and swollen, no rashes noted
- Everything else was normal

Her labs are normal

Xray of her knee didn’t show a fracture

| Diagnosis | |
| --- | --- |
| What is the diagnosis?  What do you think is going on? | ACL tear |
| How would you justify your diagnosis? | Young athlete with a deceleration injury and immediate pain and swelling. She was unable to bear weight. Her anterior drawer and Lachman’s signs are both positive, which makes ACL tear most likely |
| Evaluation/Testing | |
| Any tests you would do? | MRI knee (to plan for surgical repair) |
| How worried are you about this patient? | She is stable and her pain is controlled after acetaminophen given in ER. I am not worried at this time |
| Treatment | |
| How would you like to treat her? | Discuss with orthopedic surgery team about timing of her ACL repair.  (She will need this in order to be able to walk or play sports again) |
