# Supplementary material for: Preparing Interns as Teachers: Teaching Fourth-Year Medical Students the Tenets of the One-Minute Preceptor Model
Source: MedEdPORTAL. 2023 Dec 26;19:11371. doi: 10.15766/mep_2374-8265.11371 (PMC10749993; doi:10.15766/mep_2374-8265.11371)
Supplement: Supplementary file 1 — Intern-as-Teacher Didactic.pptxCommitment and Justification Cases.docxTeach a General Rule Cases.docxFeedback Cases.docxFull OMP Practice Cases.docxOSTE Case.docxOSTE Rubric.docxPre-Post Evaluation.docxFacilitator Guide.docx [file mep_2374-8265.11371-s001.zip › C. Teach a General Rule Cases.docx]

Appendix C: Teach a General Rule Cases

## Instructions:

- Time:
  - 20 minutes for slides and practice
    - 10 minutes for slides
    - 10 minutes for practice
- Use these cases with Slide 21 pulled up
  - There is one sheet of cases for each specialty (Medicine, Pediatrics, Surgery)
  - Will give one sheet of cases to each student in the group based on specialty of choice
- Each student will go twice
  - Student will review the sheet of cases in front of them
  - They will read the short case out loud to the group and then provide a quick, general teaching point about that case
  - If a case is used by one student in the group, it can be reused by another but needs to have a different teaching point

## Internal Medicine Teach a General Rule Case Slips

Leland Waters is a 65-year-old man with known coronary artery disease that presents with chest pain. EKG shows 3mm of ST elevation in V2 to V6 with reciprocal ST depression. You are worried about STEMI.

Rochelle Barker is a 42-year-old woman with right upper quadrant abdominal pain that is worse after eating. Ultrasound showed numerous gallstones in her gallbladder. You are worried about biliary colic from gallstones.

Vivian Payne is an 18-year-old college student that presented with headache, fever to 102, neck stiffens, and photophobia. You are concerned about meningitis.

Bennie Diaz is a 50-year-old man with history of alcohol use disorder who presents with acute abdominal pain that bores through to his back. His lipase is 500 (upper limit of normal is 50). You think Bennie has acute pancreatitis.

Kelvin Reyes is a 45-year-old man with nasal congestion for 2 days. His drainage is yellow and copious. No cough or fever. No tenderness to palpation over sinuses. You think he has a viral upper respiratory infection.

Paula Barber is a 20-year-old woman who presents for vaginal discharge after unprotected intercourse with a new partner. You are concerned Paula has a sexually transmitted infection.

Homer Woods is a 60-year-old man with concern about a mole on his back that has been changing. It is asymmetric, has an irregular border, and is darker in one area. His spouse is worried he has melanoma.

Josefina Houston is a 68-year-old woman who went through menopause at age of 56. She started having vaginal bleeding 2 weeks ago. She is worried because her sister had endometrial cancer and had similar symptoms. You think she likely has endometrial cancer too.

Sadie Holland is a 46-year-old woman that noticed a firm breast mass 2 months ago. It has not changed with her menstrual cycles. On exam, you noted a 2-centimeter mass in her right breast that is not mobile. You think Sadie might have breast cancer.

Freda Mills is a 35-year-old woman who presented with fatigue. She noted associated constipation, hair thinning, skin dryness, and constipation. Her TSH is 25 (high). You think she likely has hypothyroidism.

Barbara George is a 67-year-old woman who had her hip replaced 2 weeks ago and presents with shortness of breath. Her vital signs were significant for heart rate of 115 and oxygen saturation of 89% on room air (new). She mentions she stopped taking her enoxaparin due to pain from the shots. You think she likely has an acute pulmonary embolism.

Jane Frazier is a 54-year-old woman that presented to clinic the last 3 visits with blood pressure of >160 systolic. Her home blood pressure logs show no blood pressure below 140 systolic. Both parents and her sister have high blood pressure and take medications. Her blood pressure remains above goal and likely needs a medication to start today.

Maryanne Garcia is a 42-year-old woman that was in a motor vehicle collision resulting in severe damage to her spleen requiring a splenectomy. She is worried about what this might mean going forward.

## Pediatrics Teach a General Rule Case Slips

Natasha Moreno is a 9-month-old girl with fever to 102 at home. She has a bulging right tympanic membrane with purulent fluid behind it. You think she has acute otitis media.

Vivian Payne is a 17-year-old high school student who presented with headache, fever to 102, neck stiffness, and photophobia. You are concerned about meningitis.

Bridget Roy is an 8-year-old girl with a fever of 101.5 and sore throat. She has no cough. On exam, you note anterior cervical lymphadenopathy and exudates on tonsils. You diagnose her with Strep pharyngitis.

Paula Barber is a 17-year-old woman who presents for vaginal discharge after unprotected intercourse with a new partner. You are concerned Paula has a sexually transmitted infection.

Leigh Lucas is a 15-year-old girl presents after a suicide attempt by cutting her wrists with a razor blade. She continues to endorse suicidal ideation.

Dean Matthews is a 13-year-old with abdominal pain that started at the umbilicus and moved to his right lower quadrant. He has associated nausea and vomiting. On exam, you note some right lower quadrant tenderness and rebound. You think he likely has acute appendicitis.

Dan Moralex is a 14-year-old boy that presented with acute testicular pain he noted began during a soccer game. On exam, his right testicle is firm, extremely tender to palpation, and swollen. He is not sexually active. You are worried about testicular torsion.

Gabriel Saunders is a 14-day-old that presents with a fever of 103 at home. Given his age you are worried about this high fever could mean he has an infection.

Allen Vassily is a 3-year-old who present with rectal bleeding that coats his stools. He has pain with bowel movements and blood on wipes when mom changes diapers. On exam you find an anal fissure and think his pain and blood are from constipation causing an anal fissure.

Rosalie Carroll is a 4-year-old girl who presents with 6 days of fevers to 102, anorexia, and irritability. Her exam shows bilateral conjunctivitis (no discharge) with erythema of her lips, tongue, and pharynx. You are concerned she has Kawaski disease.

Gene Poole is a 1-week-old boy who presents with bilious emesis and lethargy. He has abdominal distension on exam and blood in his diaper. You are concerned he has malrotation.

## Surgery Teach a General Rule Case Slips

Rochelle Barker is a 42-year-old woman with right upper quadrant abdominal pain that is worse after eating for several months but now is having severe RUQ pain with vomiting. Ultrasound showed stranding around her gallbladder with a leukocytosis. You are concerned about acute cholecystitis.

Barbara McKinzie is a 24-year-old woman that is 32 weeks pregnant that slipped on the ice coming into work. She is now having abdominal pain and started having some vaginal bleeding. You are concerned about placental abruption.

Dean Matthews is a 13-year-old with abdominal pain that started at the umbilicus and moved to his right lower quadrant. He has associated nausea and vomiting. On exam, you note some right lower quadrant tenderness and rebound. You think he likely has acute appendicitis.

Sadie Holland is a 46-year-old woman that noticed a firm breast mass 2 months ago. It has not changed with her menstrual cycles. On exam, you noted a 2-centimeter mass in her right breast that is not mobile with 2 palpable lymph nodes in her right axilla. She has never had a mammogram. You think Sadie might have breast cancer.

Barbara George is a 67-year-old woman who had her hip replaced 2 weeks ago and presents with shortness of breath. Her vital signs were significant for heart rate of 115 and oxygen saturation of 89% on room air (new). She mentions she stopped taking her enoxaparin due to pain from the shots. You think she likely has an acute pulmonary embolism.

Maryanne Garcia is a 42-year-old woman that was in a motor vehicle collision resulting in severe damage to her spleen requiring a splenectomy. She is worried about what this might mean going forward.

Linda Joseph is a 64-year-old woman with no known past medical history who presents with right hip pain after a fall. She takes no medications and all her labs are normal. Her exam shows an externally rotated hip that is tender to palpation. You are concerned about a right hip fracture.

Esther Bryant is a 37-year-old woman with PMH of 3 C-sections who presents with 1-day of nausea, bilious vomit, and crampy abdominal pain. She has not had a bowel movement in a few days and is not passing gas. Her abdomen is soft, but distended without guarding or rebound. You are concerned she has a small bowel obstruction.

Drew Todd is an 18-year-old man who presents with right knee redness and swelling after a fall with abrasion last week. He has limited range of motion on exam with significant erythema and swelling noted. His labs show a leukocytosis and elevated ESR/CRP. You are concerned about septic arthritis.

Sheldon Mann is a 1-week-old boy who presents with bilious emesis and lethargy. He has abdominal distension on exam and blood in his diaper. You are concerned he has malrotation.

Don Wilkerson is a 34-year-old man who presents with 1-day of groin pain that developed while exercising. He has a non-erythematous (though tender) bulge on his medial thigh below the right inguinal ligament. You are concerned about an incarcerated femoral hernia.
