# Supplementary material for: Preparing Interns as Teachers: Teaching Fourth-Year Medical Students the Tenets of the One-Minute Preceptor Model
Source: MedEdPORTAL. 2023 Dec 26;19:11371. doi: 10.15766/mep_2374-8265.11371 (PMC10749993; doi:10.15766/mep_2374-8265.11371)
Supplement: Supplementary file 1 — Intern-as-Teacher Didactic.pptxCommitment and Justification Cases.docxTeach a General Rule Cases.docxFeedback Cases.docxFull OMP Practice Cases.docxOSTE Case.docxOSTE Rubric.docxPre-Post Evaluation.docxFacilitator Guide.docx [file mep_2374-8265.11371-s001.zip › D. Feedback Cases.docx]

Appendix D: Feedback Cases

## Instructions:

- Time:
  - 25 minutes for slides and practice
    - 10 minutes for slides
    - 15 minutes for practice
- Use these cases with Slide 29 pulled up
  - There are 5 cases for each specialty (Medicine, Pediatrics, Surgery)
  - Will give one packet of 5 cases to each group
- Each student will take one case
  - The student will read their case verbatim to one other member of the group
  - The person listening to the case will be the ‘intern’ and will provide feedback based on the presentation to the person reading the case
  - Every member of the group will play both roles

## Internal Medicine Feedback Cases

1. Headache

Tracey Flowers is a 31-year-old woman who presents with new headaches. They first began 2 months ago when she had a severe headache that required her to leave work. She went home and slept, and it went away. She thought she was just dehydrated. Then, she was fine until yesterday. She developed a severe, pounding headache across her forehead. It came on gradually over about 15 minutes and lasted for 6 hours. She again left work, took 2 ibuprofen, and went to lie down. She had trouble sleeping but it improved during that time. It finally went away later that evening. She has no other complaints but does note recent nasal congestion, and her 4-year-old son has been sick with a cold. She has no past medical history and takes no medications. No allergies. No alcohol or drug use.

On exam she is:

- Afebrile, heart rate 85, blood pressure 116/84
- She is alert, well-appearing
- HEENT exam has moist mucous membranes, normal pupils
- Normal heart sounds, no murmurs
- Normal breath sounds
- Abdomen benign
- On neuro exam, she has normal cranial nerves 2 through 12, normal reflexes, normal strength throughout, normal finger to nose
- I tried to look in her eyes and they seem normal

My assessment is that this is a 31-year-old healthy woman with a new headache. I think tension headache is most likely but I would get an MRI to make sure there’s nothing serious causing it.

2. Fever

Veronica Doyle is a 64-year-old woman with diabetes and COPD, admitted for AKI on CKD who developed a fever. She spiked to 102.1 last night. They gave her Tylenol and it came down to 100.6. This morning she was 100.8. She says she “feels fine.” Her vitals had a fever. Her exam was normal. Her labs are pending. My assessment is: this is a 64-year-old woman with diabetes, COPD, and AKI on CKD who has a new fever. My differential for the fever is:

1. Sepsis from an infected catheter- She just had tunneled dialysis catheter placed yesterday. I would draw two sets of blood cultures.
2. UTI- She has a Foley. I would check a UA.
3. Pneumonia- She has no symptoms but I would get a chest x-ray anyway.
4. I don’t think she has cellulitis or an abscess.
5. It could be non-infectious, but I think infection is more likely.

She seems stable, so I think we can work it up before starting antibiotics.

3. Abdominal pain

Loraine Allison is 45-year-old woman who presents with belly pain. It’s primarily in her right upper quadrant. No vomiting or diarrhea. In the ED, she’s had labs and an x-ray so far. She received IV fluids and morphine for pain. They contacted us to admit her. Her vital signs are heart rate 92, blood pressure 110/68, no fever.

On my exam:

- She is uncomfortable but alert, not particularly ill appearing
- Normal heart and lung sounds
- Abdomen is non-distended, tender to palpation in right upper quadrant with no rebound or guarding, Murphy’s sign negative
- Brown stool on rectal exam

Labs are normal, including her white count and liver function tests.

Her x-ray showed a normal gas pattern.

In summary, this is a 45-year-old healthy woman with acute right upper quadrant pain.

My differential is:

#1 Cholecystitis

#2 Cholangitis

#3 Choledocholithiasis

For next step, I’m not sure, but I think we should order a CT scan of her abdomen.

4. Confusion

Merle Banks is a 72-year-old man admitted to the hospital after a hip fracture, who developed a UTI, and is now confused. His confusion began around midnight. He was awake and saying things that didn’t make sense. He was awake most of the night. Early this morning, he became combative and required restraints. When I went to see him, he is able to answer questions but is still confused. He denies abdominal pain, dysuria, cough, shortness of breath or palpitations. He’s had no medication changes since yesterday. His vitals have a Tmax of 100.8, heart rate 95-118, and normal blood pressure. His exam was unchanged from prior. His labs are pending.

So, this is a 72-year-old man with hip fracture and UTI, now with altered mental status due to unknown cause. My plan would be to check a CBC, electrolytes, UA, CXR, blood culture and an ABG.

5. Anemia

Kristopher Lucas is 62-year-old man admitted to the hospital for alcohol withdrawal who now has anemia. He reports that his cravings and withdrawal symptoms are controlled. He denies any vomiting, blood in his stool, or other bleeding. He denies shortness of breath or palpitations. He denies abdominal pain or lightheadedness. His vitals are temp 98.4, heart rate 108, blood pressure 146/90. On exam, he is calm and has no tremors. His sclera are pale. His heart and lungs sounds are normal. His abdomen is soft and non-tender. No rashes or bruising.

His labs are notable for hemoglobin of 7.8 with a high MCV of 105. His hemoglobin was 9.2 yesterday with a similar MCV.

I definitely wouldn’t transfuse him right now because he’s not less than 7 and not having symptoms. We do need to figure out the cause, though. So, my differential is anemia due to bleeding or some bone marrow process. He reports no bleeding. We could confirm that with a rectal exam and then consider a bone marrow biopsy.

## Pediatrics Feedback Cases

1. Fever

Molly Bryant is a 12-year-old girl admitted for an asthma exacerbation who has now developed a fever. She spiked to 102.1 last night. They gave her Tylenol and it came down to 100.6. This morning she was 100.8. She says she’s “about the same.” Her vitals were only notable for fever. Her exam was unchanged. My assessment is that this is a 9-year-old girl with asthma exacerbation who has a new fever. My differential for her fever is:

1. Pneumonia- Her initial chest x-ray was clear but we should probably repeat it since it could be related to her asthma exacerbation.
2. UTI- Since it’s common, I would check a UA.
3. I don’t think she has strep throat or cellulitis.
4. It could be non-infectious, but I think infection is more likely.

She seems stable, so I think we should work it up before starting antibiotics.

2. Sore throat

Timothy Garrett is a 5-year-old boy with a sore throat. He started complaining about it yesterday and it has gotten worse. So, today he’s not eating well, but he’s still drinking well and urinating normally. His parents gave him ibuprofen for the pain which helped some. He has had some nasal congestion and a dry cough. He told me he’d had a little trouble breathing, but dad says he hasn’t noticed anything. He hasn’t had a fever at home, but he does now. Multiple kids in his daycare have been sick recently with colds. Dad was concerned because he’s not eating and “not acting himself.” On exam, his temperature is 101.8, heart rate 96. He looks really tired. He will interact but prefers to just lie there. The back of his throat is all red with a little bit of white on the sides and big tonsils. I think he might have swollen lymph nodes in his neck. Heart sounds are normal, no murmurs. Breath sounds are clear. Abdomen is benign. No rashes.

Overall, this is a 5-year-old boy with pharyngitis. Given his exposure to kids with colds, that’s probably most likely. We could consider doing a strep test, though.

3. Rash

Amber Jensen is a 2-month-old infant who presents with a rash. Mom notes that it first started on her neck 2 days ago and has gotten a little worse and spread to her back. She has not been particularly fussy and has had normal interactions with mom. She’s had no fever and no viral symptoms like runny nose or cough. She has been breastfeeding, stooling, and urinating well. Mom reports no new soaps or detergents. She exclusively breastfeeds, and mom has had no new food exposures. On exam, she is afebrile and playful. She’s well appearing other than the rash.

My assessment is that this is a healthy 2-month-old girl with a new rash of unknown cause. Since she’s doing well overall and doesn’t appear sick, I think we can reassure mom that this rash isn’t worrisome and what new symptoms to look out for.

4. Abdominal pain

Lance Higgins is 7-year-old boy who presents with belly pain. It’s “all over.” No vomiting or diarrhea. Tylenol hasn’t helped. His vital signs are heart rate 68, blood pressure 102/58, no fever.

On my exam:

- He seems uncomfortable but not particularly ill appearing
- He appears to have normal development and nutritional status
- Normal HEENT exam, no swollen lymph nodes, no throat changes
- Normal heart and lung sounds
- Abdomen has normal bowel sounds, a little distended, mildly tender to palpation throughout, no guarding or rebound

My differential for this 7-year-old boy with acute abdominal pain is:

#1 Constipation

#2 Gastroenteritis

#2 Appendicitis

Even though appendicitis is less likely, it’s a “can’t miss” diagnosis, so I would order a CT scan of his abdomen.

5. Vaginal discharge

Kayla Clarke is a 16-year-old girl who presents with vaginal discharge. She notes that it started 4 days ago with some vaginal itching. Then, 3 days ago she started having a whitish/yellow, foul-smelling discharge. Yesterday, she had a little bit of pain with urination. She has had no fever, abdominal pain, or back pain. She denies urinary frequency. She has no past medical history and takes no medications. She is doing well in school. On exam, she is afebrile, heart rate 82, blood pressure 114/72. Normal HEENT exam, normal heart sounds, normal breath sounds, abdomen is soft and non tender. I figured we could do her pelvic exam together.

My differential is:

1. Bacterial vaginosis
2. Chlamydia
3. Gonorrhea
4. Candida
5. Trichomonas

So, I think we should do a pelvic exam and send samples for all of these. I would probably empirically treat her with antibiotics while we wait on those to come back.

## Surgery Feedback Cases

1. Vomiting

Carolyn Vargas is a 67-year-old woman who presents with vomiting. It began this morning when she woke up, though she felt nauseous and bloated yesterday. She has vomited 3 times with no blood and no bile. She’s had no bowel movement in 5 days and hasn’t passed gas since yesterday. She feels her belly is swollen and tight. She denies fever. She hasn’t had anything to eat or drink since yesterday evening. Her past medical history is significant for laparotomy after a MVC as well as an appendectomy. She takes no medicines. No alcohol or substance use.

On exam:

- Afebrile, heart rate 114, blood pressure 108/74, respirations 22, normal sats
- Ill-appearing but alert, normal GCS
- Normal heart and lung sounds
- Abdomen is distended with increased bowel sounds, tympanic, tender to palpation with some guarding in all 4 quadrants
- Rectal exam with no stool
- Normal perfusion, no edema

CBC and BMP are pending.

So, this is a 67-year-old woman with vomiting and abdominal distension. The differential is broad. I would give her Zofran and wait on the labs to come back before we do anything else.

2. Fever

Claude Garner is a 73-year-old woman with a hip fracture s/p repair 2 days ago who developed a fever overnight. She spiked to 102.1 early this morning. They gave her Tylenol and it has since come down to 100.8. She says she “feels fine.” Her vitals were notable for fever. Her exam was unchanged. Her labs are pending. My assessment is that this is a 73-year-old woman with hip arthroplasty who has a new fever, possibly sepsis. I worry this could be an infected hip hardware. So, I think we need to image her hip with a CT and see if there is fluid there. It could also be sepsis from another cause. So, I would get blood cultures, chest x-ray, and a UA. CBC is already pending. We should check an ultrasound of her legs. I would start her on empiric antibiotics while we’re working her up.

3. Pre-Op

Heidi Swanson is a 36-year-old woman being seen for pre-op evaluation prior to partial thyroidectomy. Her only medical history is low risk papillary thyroid cancer. She takes no medications and has no drug allergies. She is very active and has no chest pain or shortness of breath with exertion. She’s had no recent fever or weight loss. She doesn’t smoke. On exam, she is well appearing. Normal HEENT. Normal heart and lung sounds. Benign abdomen. She has a palpable mass in her right thyroid that is non mobile and non tender. Her thyroid ultrasound showed a 2-centimeter tumor with no nodes involved. In summary, she’s a 36-year-old woman here for pre-op evaluation. For protocol, I would get an echo. We could consider a stress test.

4. Abdominal pain

Lorene Allison is 45-year-old woman who presents with belly pain. It’s primarily in her right upper quadrant. No vomiting or diarrhea. In the ED, she’s had labs and an x-ray so far. She received IV fluids and morphine for pain. They contacted us to admit her. Her vital signs are heart rate 92, blood pressure 110/68, no fever.

On my exam:

- She is uncomfortable but alert, not particularly ill appearing
- Normal heart and lung sounds
- Abdomen is non-distended, tender to palpation in right upper quadrant with no rebound or guarding, Murphy’s sign negative
- Brown stool on rectal exam

Labs are normal, including her white count and liver function tests.

Her x-ray showed a normal gas pattern.

In summary, this is a 45-year-old healthy woman with acute right upper quadrant pain.

My differential is:

#1 Cholecystitis

#2 Cholangitis

#3 Choledocholithiasis

For next step, I’m not sure, but I think we should order a CT scan of her abdomen.

5. Confusion

Pablo Hill is a 72-year-old man admitted to the hospital after an MVC with a traumatic pneumothorax and spleen laceration, who is now confused. His confusion began around midnight. He was awake most of the night. Early this morning, he became combative and required restraints. When I went to see him, he is able to answer questions but is still confused. He denies abdominal pain, dysuria, or palpitations. His breathing is about the same, no coughing. He’s had no medication changes since yesterday. His vitals have a Tmax of 100.8, heart rate 95-118, and normal blood pressure. Normal heart and lung sounds. Abdomen is still a little tender in the left upper quadrant but no change. His chest and belly bruising is unchanged. His chest tube box is still on water seal. Labs are pending.

So, this is a 72-year-old man with pneumothorax and spleen laceration, now with altered mental status due to unknown cause. My plan would be to check a CBC, electrolytes, UA, chest x-ray, blood culture and an ABG.
