# Supplementary material for: Preparing Interns as Teachers: Teaching Fourth-Year Medical Students the Tenets of the One-Minute Preceptor Model
Source: MedEdPORTAL. 2023 Dec 26;19:11371. doi: 10.15766/mep_2374-8265.11371 (PMC10749993; doi:10.15766/mep_2374-8265.11371)
Supplement: Supplementary file 1 — Intern-as-Teacher Didactic.pptxCommitment and Justification Cases.docxTeach a General Rule Cases.docxFeedback Cases.docxFull OMP Practice Cases.docxOSTE Case.docxOSTE Rubric.docxPre-Post Evaluation.docxFacilitator Guide.docx [file mep_2374-8265.11371-s001.zip › E. Full OMP Practice Cases.docx]

Appendix E: Full OMP Practice Cases

## Instructions:

- Time:
  - 25 minutes for slides and practice
    - 10 minutes for slides
    - 15 minutes for practice
- Use these cases with Slide 35 pulled up
  - There are 5 cases for each specialty (Medicine, Pediatrics, Surgery)
  - Will give one packet of 5 cases to each group
- Each student will take one case
  - The student will read their case verbatim to one other member of the group
  - The person listening to the case will be the ‘intern’ and will perform all steps of the OMP based on the case read to them
  - Should try to make as conversational as possible
  - Every member of the group will play both roles

## Internal Medicine Full OMP Practice

1. Pancreatitis

Veronica Wilson is our 68-year-old woman admitted with pancreatitis. Last night she slept well. No events overnight. She said her pain is “tolerable.” She only received IV pain medicine once yesterday evening. She has mild nausea but no vomiting. Her vitals are T-max 98.8, HR 80-90s, BP 110-130s/80-90s, RR 12-14, SpO2 98-100% RA. On exam, she was comfortable and alert. Normal heart sounds without murmurs. Clear lung sounds. Her abdomen is soft and only mildly tender in the epigastrium, no guarding or rebound. Her extremities are warm and well-perfused. Her labs have a normal BMP. Her potassium was low yesterday but is now normal at 4.1. WBC is normal. In summary, this is a 68-year-old woman with acute pancreatitis, likely due to alcohol use. Hospital day #2. Her symptoms and exam are significantly improved. She remains NPO with LR running at 125ml/hr.

2. Septic arthritis

Beatrice Burke is our 48-year-old woman admitted with septic arthritis of her knee. Overnight, she had a fever. She says her knee still hurts a lot this morning. She didn’t sleep well last night. Her vitals this morning are Temp 101.2, HR 104, BP 134/86, RR 14, SpO2 100%. On exam, she was in no acute distress. Normocephalic, atraumatic. Pupils equal. Normal heart and lung sounds. Her belly was soft and nondistended. Left knee is still really swollen and red, painful with any movement. Normal cranial nerves. Labs have a sodium 138, potassium 3.6, bicarb 22, creatinine 0.6. WBC 18, hemoglobin 12.8, platelets 320. Blood cultures are no growth to date. In summary, this is a 48-year-old woman with left knee septic arthritis. She seems stable, maybe a little more comfortable than yesterday, but still having fevers. We will continue her on IV vancomycin and ceftriaxone. Await cultures.

3. Alcohol withdrawal

Joey Dixon is our 57-year-old man with alcohol withdrawal. Overnight, he required no doses of diazepam. This morning he says he feels back to normal. He feels less anxious and is not having tremors. He’s had no nausea or vomiting. No headache. His vitals this morning are T97.6, HR 82, BP 118/74, RR 12, SpO2 99%. On exam, he is calm and well appearing. His pupils are equal and reactive. His mucous membranes are moist. His heart has a normal S1 and S2. His lungs are clear to auscultation bilaterally. His abdomen is soft and nontender. On neuro exam, he has normal reflexes and strength throughout. No tremor. Normal cranial nerves 2-12. Labs show a mild anemia of 10.6, unchanged from 10.4 yesterday. His BMP and LFTs are normal. In summary, this is a 57-year-old man admitted for alcohol withdrawal, now hospital day #4. His withdrawal seems to be well controlled. He is getting CIWA scores (which have been 0 to 2) with prn diazepam, but hasn’t needed any in 24 hours. Overall, I think he’s doing well.

4. DKA

Freddie Rose is our 23-year-old man with type 1 diabetes admitted yesterday for DKA. Overnight, his glucose values decreased to low 200s and he was started on D5 drip. He feels back to normal this morning and is asking to eat. He’s had no vomiting, and his belly pain has resolved. On exam he has been afebrile, HR 80-94, BP 108-116/60-68, RR 12-14, normal SpO2. Ins and outs have been even. On my exam, he was alert and well appearing. He had moist mucous membranes, normal pupils. Heart had S1 and S2 without murmurs, rubs, or gallops. His lungs were clear. His abdomen is nontender. Normal extremities. Normal neuro exam. Glucose values were in the 200s overnight, last one this morning was 220. Anion gap is 11. Bicarb is 20. Sodium and potassium normal. In summary, this is a 23-year-old man with type 1 diabetes who presented in DKA yesterday. He’s still on the insulin drip. His anion gap is now normal. He’s on D5 to avoid hypoglycemia. His electrolytes are all stable. His next BMP will be at 11 AM.

5. Seizures

Dan Bowen is our 31-year-old man with epilepsy who was admitted with worsening seizures. Yesterday evening, he had an EEG, and those results are pending. Overnight, his sister said he had multiple brief shaking episodes but they resolved before the nurse came in. He didn’t receive any lorazepam overnight. This morning he’s sleepy. His vitals were all normal today. On exam, he has normal pupils, extraocular movements intact. Normal heart and lung sounds. Cranial nerves normal. Reflexes normal. No abnormal movements. BMP and CBC were both normal. In summary, this is a 31-year-old man with epilepsy who has worsening seizures. His levetiracetam dose was increased yesterday. He appears to still be having events, though. His EEG is pending. Neurology is following him.

## Pediatrics Full OMP Practice

1. Bronchiolitis

Madeline Palmer is our 6-month-old girl admitted yesterday with bronchiolitis. No events overnight. Mom said that she was restless but better than the previous night at home. She still has cough and runny nose. Mom doing nasal suctioning. Taking a bottle in small amounts but urinating well. Her vitals are T-max 100.8, HR 130-150, BP 86/40-92/44, RR 30-34, SpO2 98% RA on 2L O2. Urine output is 1.8 ml/kg/hr. On exam, she is fussy. Mouth is moist. Lots of clear rhinorrhea. Normal heart sounds. Lungs clear to auscultation bilaterally. No retractions or grunting. Abdomen is benign. No labs. In summary, this is a 6-month-old girl with bronchiolitis, hospital day #2. Overall, she seems to be improved from yesterday. She is still on oxygen and IV fluids.

2. Septic arthritis

Nichole Romero is our 8-year-old girl admitted with septic arthritis of her knee. Overnight, she had a fever. She says her knee still hurts a lot this morning. She didn’t sleep well last night. Her vitals this morning are Temp 101.2, HR 120, BP 100/60, RR 14, SpO2 100%. On exam, she was in no acute distress. Normocephalic, atraumatic. Pupils equal. Normal heart and lung sounds. Her belly was soft and nondistended. Left knee still really swollen and red, painful with any movement. Normal cranial nerves. Labs have a sodium 138, potassium 3.6, bicarb 22, creatinine 0.6. WBC 18, hemoglobin 12.8, platelets 320. Blood cultures no growth to date. In summary, this is an 8-year-old girl with left knee septic arthritis. She seems stable, maybe a little more comfortable than yesterday, but still having fevers. We will continue her on IV vancomycin and ceftriaxone. Await cultures.

3. DKA

Troy Hammond is our 14-year-old boy with type 1 diabetes admitted yesterday for DKA. Overnight, his glucose values decreased to low 200s and he was started on D5 drip. He feels back to normal this morning and is asking to eat. He’s had no vomiting, and his belly pain has resolved. On exam he has been afebrile, HR 80-94, BP 108-116/60-68, RR 12-14, normal SpO2. Ins and outs have been even. On my exam, he was alert and well appearing. He had moist mucous membranes, normal pupils. Heart had S1 and S2 without murmurs, rubs, or gallops. His lungs were clear. His abdomen is nontender. Normal extremities. Normal neuro exam. Glucose values were in the 200s overnight, last one this morning was 220. Anion gap is 11. Bicarb is 20. Sodium and potassium normal. In summary, this is a 14-year-old boy with type 1 diabetes who presented in DKA yesterday. He’s still on the insulin drip. His anion gap is now normal. He’s on D5 to avoid hypoglycemia. His electrolytes are all stable. His next BMP will be at 11 AM.

4. Seizures

Owen Jones is our 11-year-old boy with epilepsy who was admitted with worsening seizures. Yesterday evening, he had an EEG, and those results are pending. Overnight, his dad said he had multiple brief shaking episodes but they resolved before the nurse came in. He didn’t receive any lorazepam overnight. This morning he’s sleepy. His vitals were all normal today. On exam, he has normal pupils, extraocular movements intact. Normal heart and lung sounds. Cranial nerves normal. Reflexes normal. No abnormal movements. BMP and CBC were both normal. In summary, this is an 11-year-old boy with epilepsy who has worsening seizures. His levetiracetam dose was increased yesterday. He appears to still be having events, though. His EEG is pending. Neurology is following him.

5. Neonatal jaundice

Isabel Watkins is our 2-day-old female infant with neonatal jaundice. Last night she breastfed well. She was under phototherapy all night. She’s had 1 wet diaper since she was admitted. Her vitals this morning are T98.4, HR 144, BP 80/50, RR 20, SpO2 100%. Urine output is 1.2 ml/kg/hr. On exam, she is calm and alert. Pupils equal. Normal heart sounds, no murmurs. Lungs clear to auscultation. Abdomen nondistended, no masses. Normal suck reflex. Normal Moro. No rashes. Bili this morning was 15. It was 17 yesterday. In summary, this is a 2-day-old full-term infant with neonatal jaundice. She remains in phototherapy, and her bilirubin level is coming down. We can continue to monitor that and her urine output.

## Surgery Full OMP Practice

1. Traumatic pneumothorax

Jessie Snyder is our 27-year-old man admitted with a pneumothorax after a stab wound. Overnight, he had no events. This morning, he is still in pain in his left chest. He says he is breathing a little better than yesterday, no coughing. His vitals are afebrile, HR 80-90s, BP 128/78, RR 12-16, SpO2 100% on 6L. On exam, he appears comfortable, no distress. Normal heart sounds. Left chest has some crackles, about the same as yesterday. Chest tube in place to suction. Abdomen is benign. Normal extremities. On labs, CBC and BMP today were normal. CXR shows minimal left pneumothorax on prelim read. In summary, this is a 27-year-old man with a traumatic pneumothorax after a stab wound to his left chest. He seems stable. We will continue the chest tube and oxygen. Await final read of his CXR.

2. Diverticulitis

Joann Davis is our 79-year-old woman with perforated diverticulitis s/p resection. No events overnight. This morning, she is having a lot of pain at her surgical incision. She feels nauseous but has had no vomiting. Small amount of output from her ostomy. Her vitals this morning are T 99.8, HR 98, BP 132/88, RR 16, SpO2 100% RA. On exam, she appears uncomfortable. Her heart and lung sounds are normal. Her abdomen is soft. Nontender except near her incision, which is red with a little yellow drainage. Her colostomy bag has brown stool. On her labs today, her WBC is 16, hemoglobin 9.5, platelets 280. Her BMP is normal. KUB showed normal gas pattern yesterday. In summary, this is a 79-year-old woman with perforated diverticulitis post-op day #4 after resection and colostomy. Her pain is worse and the incision site looks concerning. We are continuing her on pip-tazo for her diverticulitis. She has prn oxycodone for pain.

3. Septic arthritis

Beatrice Burke is our 48-year-old woman admitted with septic arthritis of her knee. Overnight, she had a fever. She says her knee still hurts a lot this morning. She didn’t sleep well last night. Her vitals this morning are Temp 101.2, HR 104, BP 134/86, RR 14, SpO2 100%. On exam, she was in no acute distress. Normocephalic, atraumatic. Pupils equal. Normal heart and lung sounds. Her belly was soft and nondistended. Left knee is still really swollen and red, painful with any movement. Normal cranial nerves. Labs have a sodium 138, potassium 3.6, bicarb 22, creatinine 0.6. WBC 18, hemoglobin 12.8, platelets 320. Blood cultures are no growth to date. In summary, this is a 48-year-old woman with left knee septic arthritis. She seems stable, maybe a little more comfortable than yesterday, but still having fevers. We will continue her on IV vancomycin and ceftriaxone. Await cultures.

4. Small bowel obstruction

Brenda Schmidt is our 51-year-old woman admitted with SBO. Overnight, she had increased pain, requiring increased doses of morphine. This morning, she says her pain is 10/10, across her entire abdomen. She feels nauseous. She’s had no bowel movement or flatus. Her vitals this morning are Temp 99.1, HR 104, BP 142/88, RR 14, SpO2 98% RA. On exam, she is very uncomfortable appearing but alert. Mouth is moist. PERRL. NG tube in place to low wall suction. Heart sounds normal, no murmurs. Lungs clear to auscultation. Abdomen is distended and tympanic, diffusely tender to palpation, but no rebound or guarding. Her CBC has a stable WBC and hemoglobin. Her BMP has a high bicarb at 31 and low potassium at 3.2, creatinine normal. In summary, this is a 51-year-old woman with small bowel obstruction due to adhesions from her prior abdominal surgeries. She’s stable. We’ll keep her NPO and continue her NG tube to suction. She has IV morphine prn for pain.

5. Gallstone pancreatitis

Veronica Wilson is our 68-year-old woman admitted with gallstone pancreatitis. Last night she slept well. No events overnight. She said her pain is “tolerable.” She only received IV pain medicine once yesterday evening. She has mild nausea but no vomiting. Her vitals are T-max 98.8, HR 80-90s, BP 110-130s/80-90s, RR 12-14, SpO2 98-100% RA. On exam, she was comfortable and alert. Normal heart sounds without murmurs. Clear lung sounds. Her abdomen is soft and only mildly tender in the epigastrium, no guarding or rebound. Her extremities are warm and well-perfused. On labs, her bili continues to trend down. It was 2.4 yesterday and is 1.8 today. Her BMP and CBC are normal. Her ultrasound yesterday showed a normal common bile duct. In summary, this is a 68-year-old woman with gallstone pancreatitis. Hospital day #2. Her symptoms, exam, and labs are significantly improved. She remains NPO with LR running at 125ml/hr.
