# Supplementary material for: Preparing Interns as Teachers: Teaching Fourth-Year Medical Students the Tenets of the One-Minute Preceptor Model
Source: MedEdPORTAL. 2023 Dec 26;19:11371. doi: 10.15766/mep_2374-8265.11371 (PMC10749993; doi:10.15766/mep_2374-8265.11371)
Supplement: Supplementary file 1 — Intern-as-Teacher Didactic.pptxCommitment and Justification Cases.docxTeach a General Rule Cases.docxFeedback Cases.docxFull OMP Practice Cases.docxOSTE Case.docxOSTE Rubric.docxPre-Post Evaluation.docxFacilitator Guide.docx [file mep_2374-8265.11371-s001.zip › F. OSTE Case.docx]

***Instructions****: You will read the verbatim to the student. See second paper about answers for medical decision making*

Chief Compliant: cough

History of Present Illness:

- Mr. Matt Henderson is a 75-year-old man with cough. The cough started one week ago but has been significantly worse in the last 24 hours. Initially cough was dry and associated with nasal drainage and a sore throat. No fever initially. Thought was a cold he caught from his granddaughter that was visiting the weekend before. However, in the last 24 hours, his cough is now productive of a yellow/green sputum and having fevers. Temperature was 101 last night. Has been increasingly short of air and having difficulty getting around house yesterday and today due to his illness.

Past Medical History: Arthritis in hands

No surgical history

Medications: Acetaminophen as needed for pain in hands

Social History: Former smoker that quit 30 years ago. Denies alcohol or other illicits

Family history: His father died of a heart attack at 75 and mother had hypertension

Review of systems is positive for what was discussed before. His nasal drainage is better but his throat is still sore due to coughing. He denies chest pain.

On exam:

- Vital signs: Temperature 101.2, HR 115, BP 145/76, RR 24, SpO2 90% on RA
- General: moderate respiratory distress, elderly appearing man
- HEENT: mild pharyngeal erythema without exudates
- Cardiovascular: regular rhythm, tachycardic, no murmurs/rubs/gallops
- Respiratory: Moderately labored respirations with crackles in the right lower lung field, no wheezes appreciated
- Everything else was normal

His labs were normal except for a white count of 17.5

His EKG showed sinus tachycardia but otherwise normal.

His chest X-ray showed a right lower lobe consolidation

Assessment and plan:

- So, in summary, Mr. Matt Henderson is a 75-year-old man with cough and fever.

Medical Decision Making:

Below are answers to common questions you might be asked about this case.

| What is the diagnosis? | Sepsis due to bacterial pneumonia |
| --- | --- |
| How would you justify this diagnosis? | Has sepsis due to 4 SIRS criteria and a source of likely pneumonia. The SIRS criteria he meets are fever, leukocytosis, tachycardia and tachypnea. His CXR shows a likely source of infection as well as the exam is consistent with crackles in the right base too |
| How would you like to treat?  What antibiotic would you start and how long? | Start antibiotics.  Would like to start levofloxacin 750mg daily for 5 days |
| Should we admit the patient? | Yes – in distress from breathing, which makes sending him home a concern |
| Any tests for work up of pneumonia? | Yes – obtain a sputum sample to send for analysis. Could consider sending urine strep antigen, but do not think a urine legionella antigen is needed since this presentation is not consistent with legionella |
| Other images considered? | No – don't think he needs a CT scan as the CXR was diagnostic. EKG is not concerning for ACS. |
| Argument against COPD exacerbation | No wheezes on exam, no hyperinflated lungs on CXR, no prior breathing difficulties. Smoking history is a risk factor but not guarantee to have COPD |
| Is this patient sick or not sick? | Sick – he is in moderate respiratory distress on exam and having labored breathing. |
| How worried are you about this patient? | I am worried about this patient. He is having trouble breathing but appears to be compensating and his oxygen saturation is appropriate given his known COPD. However, if his breathing does not improve soon, will need to consider getting an ABG for more evaluation. If that is abnormal, maybe BiPAP or intubation to help with work of breathing. |
| What ‘can’t miss’ diagnosis are you considering? | Certainly an active infection is an important thing not to miss. However, a PE can present with shortness of air, cough, tachycardia and tachypnea. But his Well’s score is only 1.5 (only points given were for HR >100), making him low risk for PE. No further testing needed at this time. |
