# Supplementary material for: Preparing Interns as Teachers: Teaching Fourth-Year Medical Students the Tenets of the One-Minute Preceptor Model
Source: MedEdPORTAL. 2023 Dec 26;19:11371. doi: 10.15766/mep_2374-8265.11371 (PMC10749993; doi:10.15766/mep_2374-8265.11371)
Supplement: Supplementary file 1 — Intern-as-Teacher Didactic.pptxCommitment and Justification Cases.docxTeach a General Rule Cases.docxFeedback Cases.docxFull OMP Practice Cases.docxOSTE Case.docxOSTE Rubric.docxPre-Post Evaluation.docxFacilitator Guide.docx [file mep_2374-8265.11371-s001.zip › H. Pre-Post Evaluation.docx]

Intern as Teacher Workshop

**PRE**-workshop Evaluation

**How confident are you in being able to do each of the following:**

(place an “X” in the appropriate box for each item)

|  | **Not at all confident** | **Slightly confident** | **Moderately confident** | **Quite confident** | **Extremely confident** |
| --- | --- | --- | --- | --- | --- |
| 1. Teach students as an intern |  |  |  |  |  |
| 1. Ask questions of a student to elicit a clinical commitment |  |  |  |  |  |
| 1. Ask questions of a student to probe for their justification |  |  |  |  |  |
| 1. Provide specific feedback to a student about what they did well |  |  |  |  |  |
| 1. Provide specific feedback to a student about how they can improve |  |  |  |  |  |
| 1. Provide a brief teaching point to a student |  |  |  |  |  |

Intern as Teacher Workshop

**POST**-workshop Evaluation

**How confident are you in being able to do each of the following:**

(place an “X” in the appropriate box for each item)

|  | **Not at all confident** | **Slightly confident** | **Moderately confident** | **Quite confident** | **Extremely confident** |
| --- | --- | --- | --- | --- | --- |
| 1. Teach students as an intern |  |  |  |  |  |
| 1. Ask questions of a student to elicit a clinical commitment |  |  |  |  |  |
| 1. Ask questions of a student to probe for their justification |  |  |  |  |  |
| 1. Provide specific feedback to a student about what they did well |  |  |  |  |  |
| 1. Provide specific feedback to a student about how they can improve |  |  |  |  |  |
| 1. Provide a brief teaching point to a student |  |  |  |  |  |
