# Supplementary material for: Preparing Interns as Teachers: Teaching Fourth-Year Medical Students the Tenets of the One-Minute Preceptor Model
Source: MedEdPORTAL. 2023 Dec 26;19:11371. doi: 10.15766/mep_2374-8265.11371 (PMC10749993; doi:10.15766/mep_2374-8265.11371)
Supplement: Supplementary file 1 — Intern-as-Teacher Didactic.pptxCommitment and Justification Cases.docxTeach a General Rule Cases.docxFeedback Cases.docxFull OMP Practice Cases.docxOSTE Case.docxOSTE Rubric.docxPre-Post Evaluation.docxFacilitator Guide.docx [file mep_2374-8265.11371-s001.zip › I. Facilitator Guide.docx]

Appendix I: Facilitator Guide

Session Overview:

This is a 2-hour workshop designed to teach the One-Minute Preceptor (OMP) model to 4^th^ year medical students by separating the skills into sections and allowing practice of each before putting together the entire OMP model. The session has a Power Point presentation and cases for each section. The cases are grouped by adult medicine, pediatrics, and surgery based on the student’s planned residency.

Session objectives:

By the end of this session, students will be able to:

- List the steps in the one-minute preceptor model
- Ask questions of a student to get a clinical commitment and justification
- Provide a brief teaching point to a student
- Provide specific, balanced feedback to a student

Pre-reading for attendees:

- Neher JO, Gordon KC, Meyer B, Stevens N. A five-step “microskills” model of clinical teaching. *J Am Board Fam Pract*. 1992;5(4):419-24. PMID: 1496899.

Before the Session:

1. Print materials
   1. One pre/post evaluation (Appendix H) with paired numbers per student
   2. Handout (one for each student)
   3. Cases for all 4 exercises
      1. Each group will need one copy of each exercise (Appendices B, C, D, and E)
         1. E.g. if you have 7 groups, will need 7 copies of all appendices
      2. Specific to the track for that workshop
2. Morning of
   1. Lay out papers according to where students will sit
   2. Goal is groups of 4-5 students
   3. Pull up PowerPoint (follows the outline below with speaker notes)

Session Outline:

| **Time** | **Topic** |
| --- | --- |
| 10 min | **Introduction to and orientation** |
| 25 min | **Commitment and justification cases** |
| 20 min | **Teaching cases** |
| 25 min | **Feedback cases** |
| 25 min | **Full OMP Practice** |
| 10 min | **Wrap-up** |

**Power Point Presentation**

- Detailed notes for each slide can be found in the slide deck (Appendix A)
- Groups should be 4-5 students per group as there are a maximum of 5 cases per exercise and each student needs their own case in each practice

Introduction and Orientation (10 minutes)

- Slides 1 – 10
- Start with reflections on M3. The goal is to get them talking about their experiences as students and have the reflect on effective teachers the worked with to stimulate participation early in the session.
- Introduce topic of OMP and that it is popular to teach due to the evidence to support it (1 systematic review and 1 RCT). Emphasizing that teaching is a skill and practice is key to improving that skill.
- Describe how the students will practice this skill. They will use role play and clinical cases to practice being the 3^rd^ year student and the ‘intern’ teaching them. Will break the OMP apart into sections before practicing all together:
  - Ask for a commitment and justification
  - Provide a brief teaching point
  - Provide reinforcing and constructive feedback
  - Perform the entire OMP
- While this can seem easy to master the skill takes time and practice (hence the multiple cases and practice sessions)

Commitment and Justification Cases (25 minutes)

- Slides 11 – 17
  - Details the logistics of getting a commitment and asking for a justification
  - Then will practice in their assigned small groups
- Provide the printed copies ‘Appendix B: Commitment and Justification Cases’ to each group (1 case per student)
- Each student will take one case
  - The student will read their case verbatim to one other member of the group
  - The person listening to the case will be the ‘intern’ and ask the person reading the case to commit to something and then ask them to justify that commitment
  - Should only be one commitment and one justification per case
  - Every member of the group will play both roles
- Debrief this skill

Teaching a General Rule Cases (20 minutes)

- Slides 18 – 22
  - Details the logistics of teaching a general rule
  - Then will practice in their assigned small groups
- Provide the printed copies ‘Appendix C: Teaching a General Rule Cases’ to each group (1 sheet per student)
- Each student will go twice
  - Student will review the sheet of cases in front of them
  - They will read the short case out loud to the group and then provide a quick, general teaching point about that case
  - If a case is used by one student in the group, it can be reused by another but needs to have a different teaching point
- Debrief this skill

Feedback Cases (25 minutes)

- Slides 23 – 30
  - Details the logistics of providing both reinforcing and constructive feedback
  - Then will practice in their assigned small groups
- Provide the printed copies ‘Appendix D: Feedback Cases’ to each group (1 case per student)
- Each student will take one case
  - The student will read their case verbatim to one other member of the group
  - The person listening to the case will be the ‘intern’ and will provide feedback based on the presentation to the person reading the case
  - Every member of the group will play both roles
- Debrief this skill

Full OMP Practice (25 minutes)

- Slides 31 – 36
  - Details the logistics of performing the entire OMP
  - Then will practice in their assigned small groups
- Provide the printed copies ‘Appendix E: Full OMP Practice Cases’ to each group (1 case per student)
- Each student will take one case
  - The student will read their case verbatim to one other member of the group
  - The person listening to the case will be the ‘intern’ and will perform all steps of the OMP based on the case read to them
  - Should try to make as conversational as possible
  - Every member of the group will play both roles
- Debrief the skill

Wrap Up (10 minutes)

- Slides 37 – 39
- OSTE assessment:
- Case will be presented by a standardized patient (SP) who will be playing a third-year student (named Sam Russell)
- Assessed by SP via rubric (included on slide 39 and in Appendix G)
