# Supplementary material for: Preparing Interns as Teachers: Teaching Fourth-Year Medical Students the Tenets of the One-Minute Preceptor Model
Source: MedEdPORTAL. 2023 Dec 26;19:11371. doi: 10.15766/mep_2374-8265.11371 (PMC10749993; doi:10.15766/mep_2374-8265.11371)
Supplement: Supplementary file 1 — Intern-as-Teacher Didactic.pptxCommitment and Justification Cases.docxTeach a General Rule Cases.docxFeedback Cases.docxFull OMP Practice Cases.docxOSTE Case.docxOSTE Rubric.docxPre-Post Evaluation.docxFacilitator Guide.docx [file mep_2374-8265.11371-s001.zip › A. Intern-as-Teacher Didactic.pptx]

## Slide 1
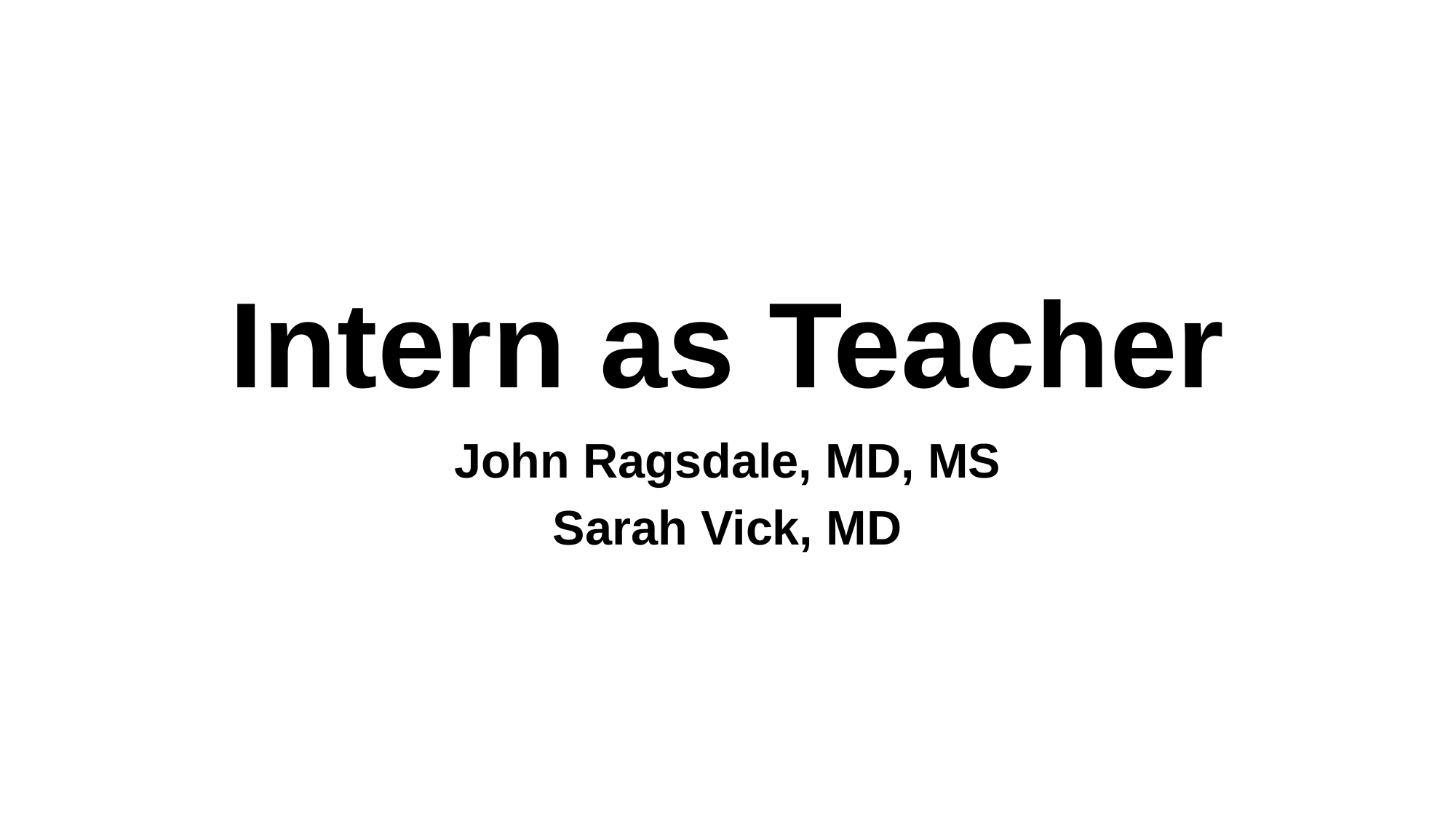

# Intern as Teacher
John Ragsdale, MD, MS
Sarah Vick, MD

## Slide 2
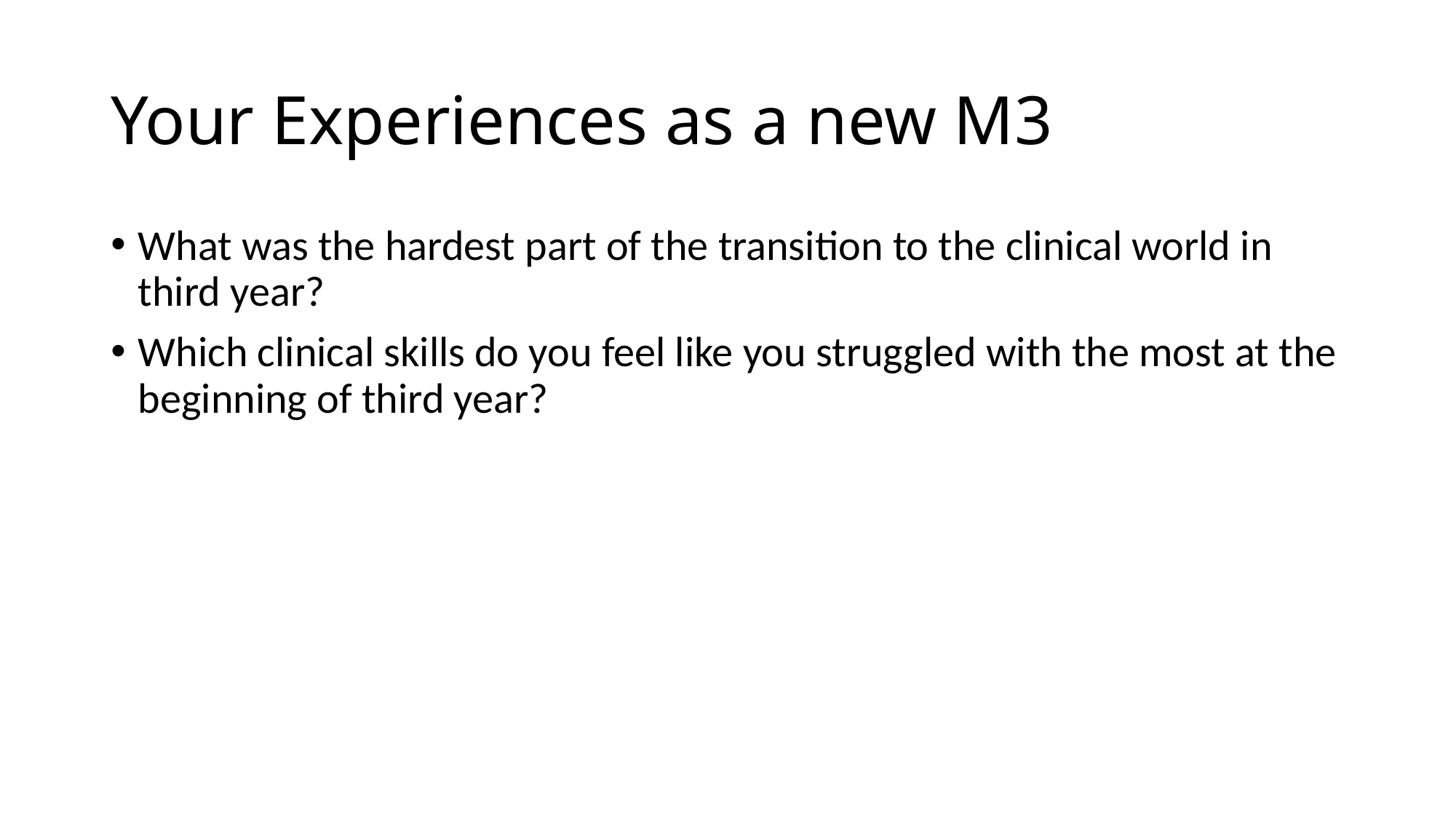

# Your Experiences as a new M3
What was the hardest part of the transition to the clinical world in third year?
Which clinical skills do you feel like you struggled with the most at the beginning of third year?

## Slide 3
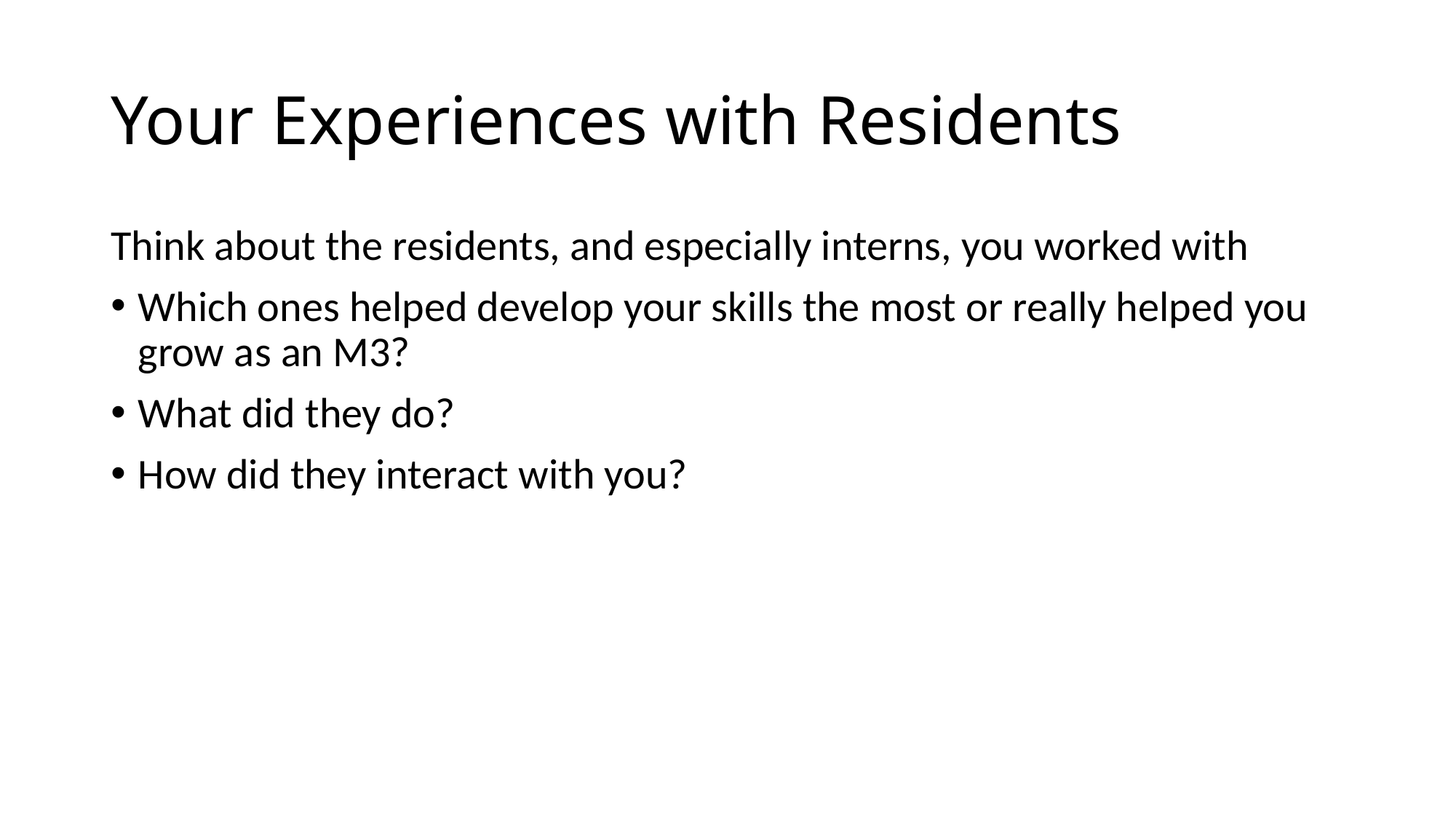

# Your Experiences with Residents
Think about the residents, and especially interns, you worked with
Which ones helped develop your skills the most or really helped you grow as an M3?
What did they do?
How did they interact with you?

## Slide 4
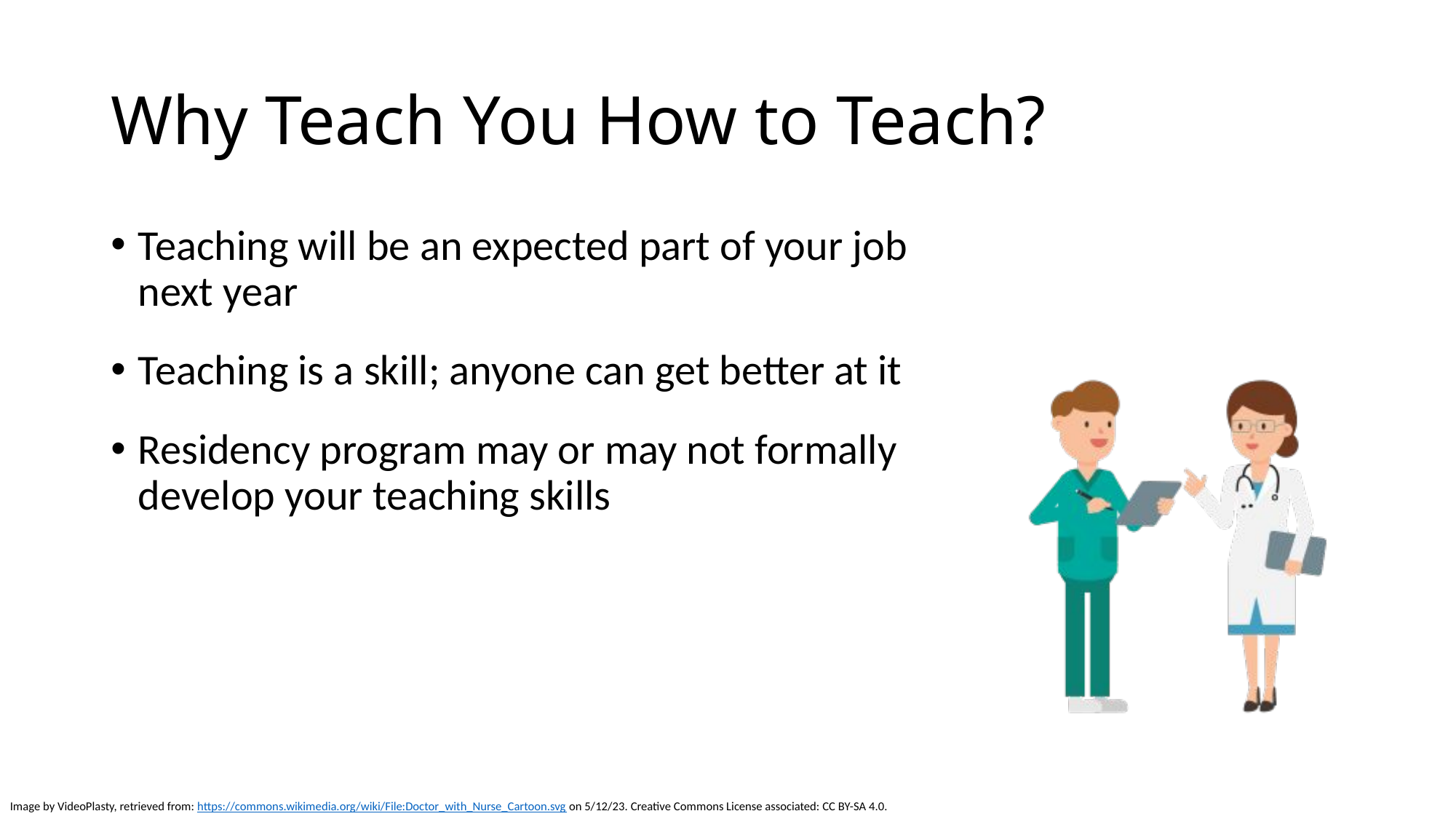

# Why Teach You How to Teach?
Teaching will be an expected part of your job next year
Teaching is a skill; anyone can get better at it
Residency program may or may not formally develop your teaching skills
Image by VideoPlasty, retrieved from: https://commons.wikimedia.org/wiki/File:Doctor_with_Nurse_Cartoon.svg on 5/12/23. Creative Commons License associated: CC BY-SA 4.0.

## Slide 5
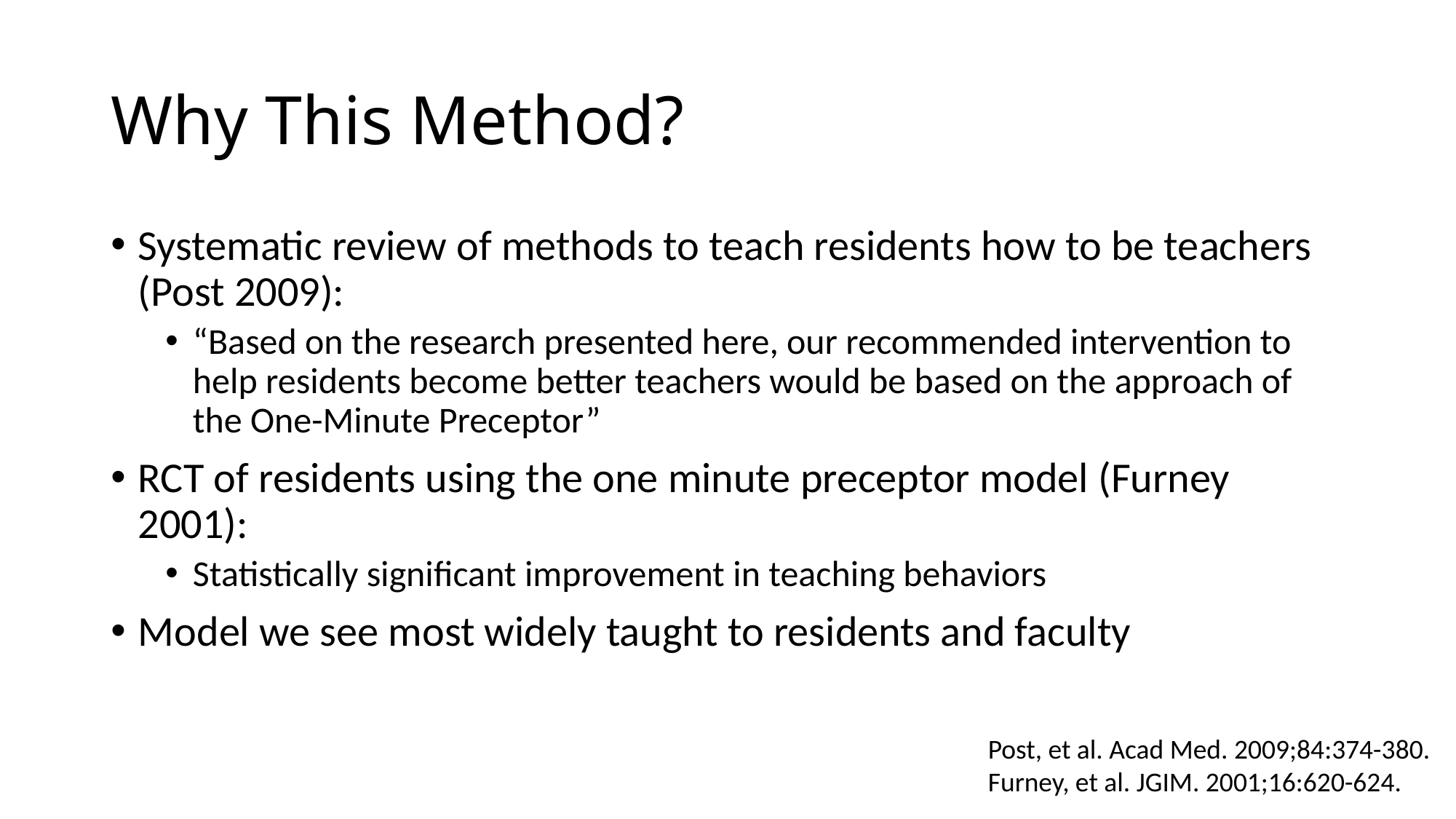

# Why This Method?
Systematic review of methods to teach residents how to be teachers (Post 2009):
“Based on the research presented here, our recommended intervention to help residents become better teachers would be based on the approach of the One-Minute Preceptor”
RCT of residents using the one minute preceptor model (Furney 2001):
Statistically significant improvement in teaching behaviors
Model we see most widely taught to residents and faculty
Post, et al. Acad Med. 2009;84:374-380.
Furney, et al. JGIM. 2001;16:620-624.

## Slide 6
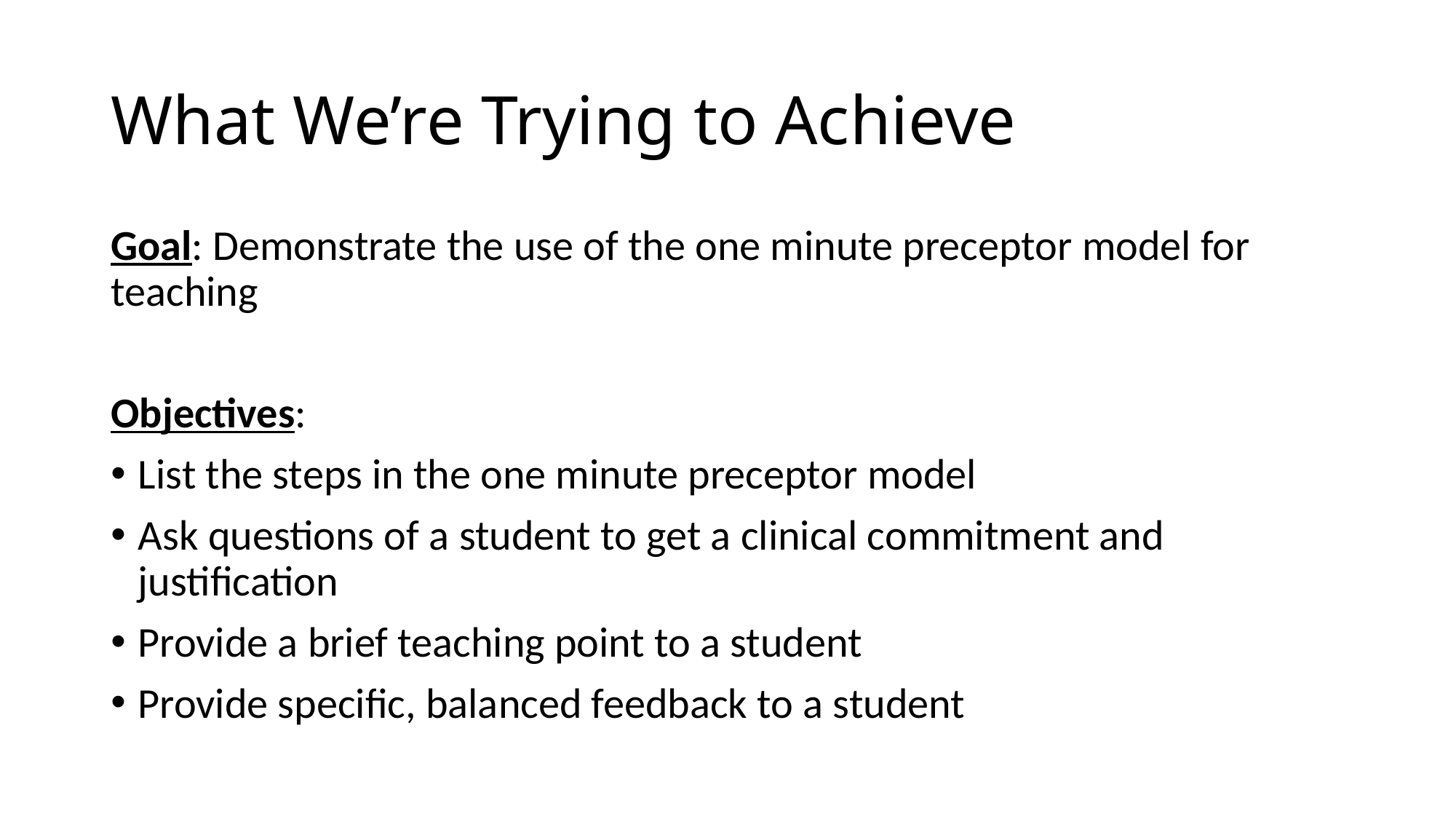

# What We’re Trying to Achieve
Goal: Demonstrate the use of the one minute preceptor model for teaching
Objectives:
List the steps in the one minute preceptor model
Ask questions of a student to get a clinical commitment and justification
Provide a brief teaching point to a student
Provide specific, balanced feedback to a student

## Slide 7
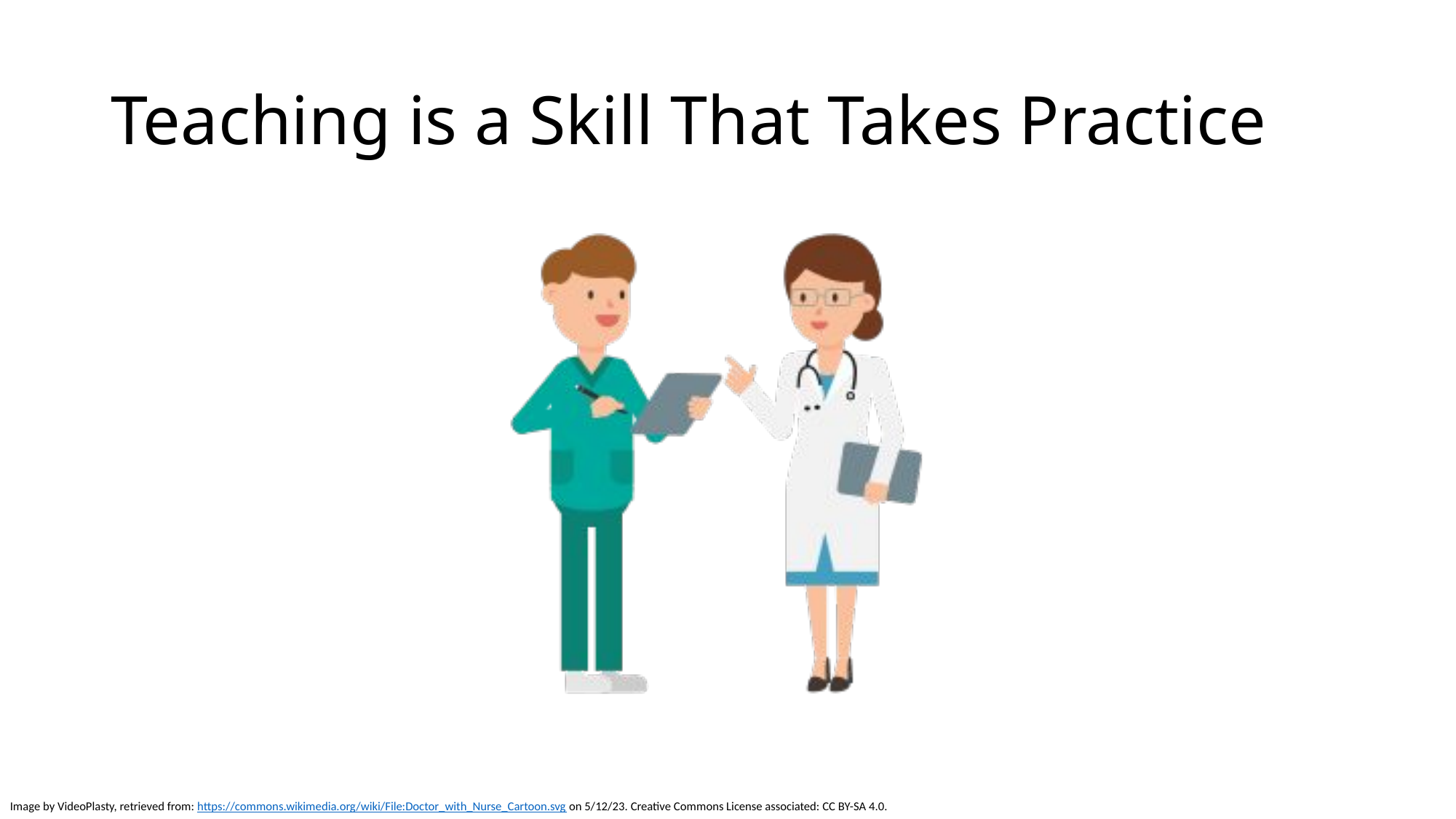

# Teaching is a Skill That Takes Practice
Image by VideoPlasty, retrieved from: https://commons.wikimedia.org/wiki/File:Doctor_with_Nurse_Cartoon.svg on 5/12/23. Creative Commons License associated: CC BY-SA 4.0.

## Slide 8
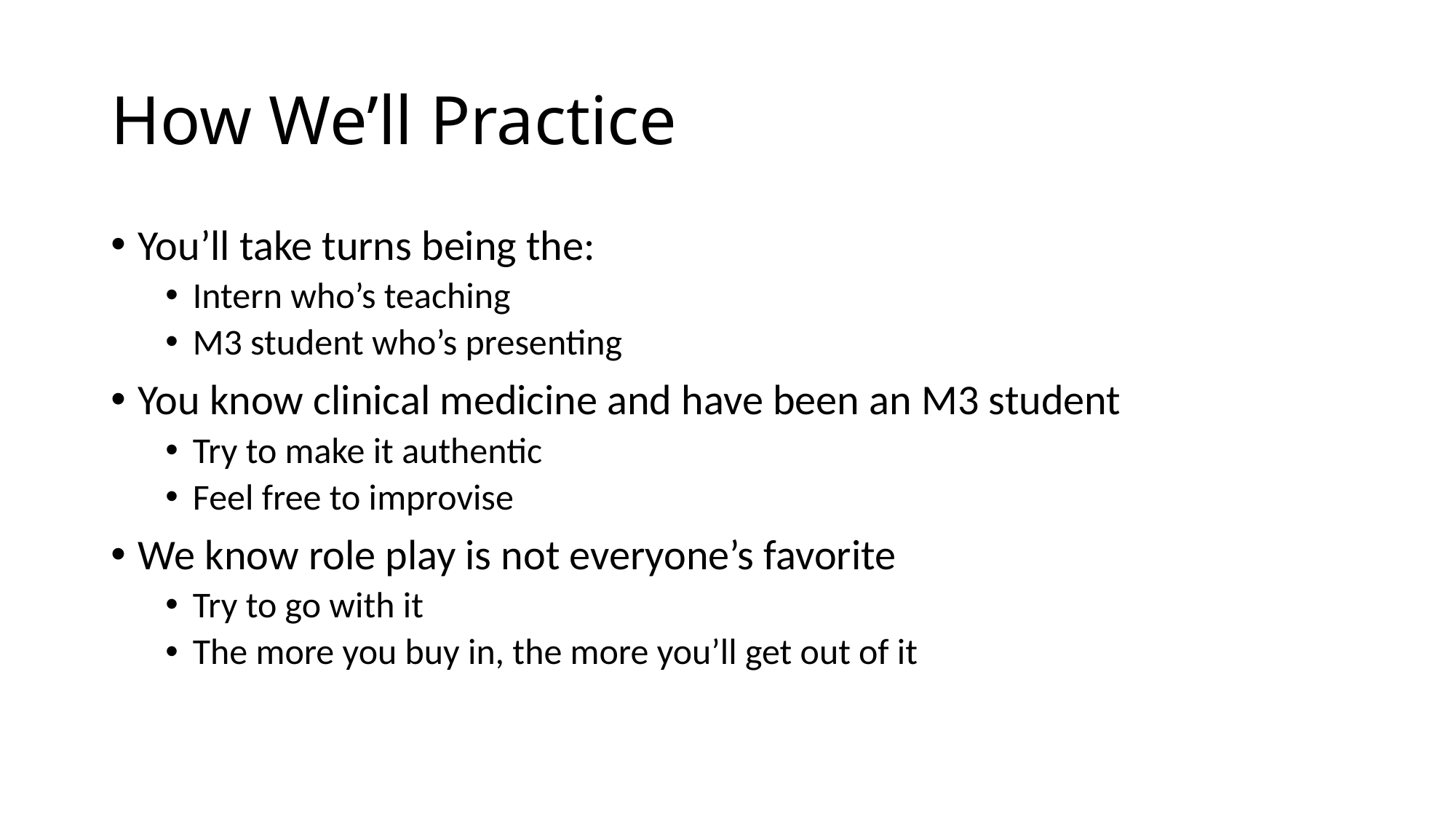

# How We’ll Practice
You’ll take turns being the:
Intern who’s teaching
M3 student who’s presenting
You know clinical medicine and have been an M3 student
Try to make it authentic
Feel free to improvise
We know role play is not everyone’s favorite
Try to go with it
The more you buy in, the more you’ll get out of it

## Slide 9
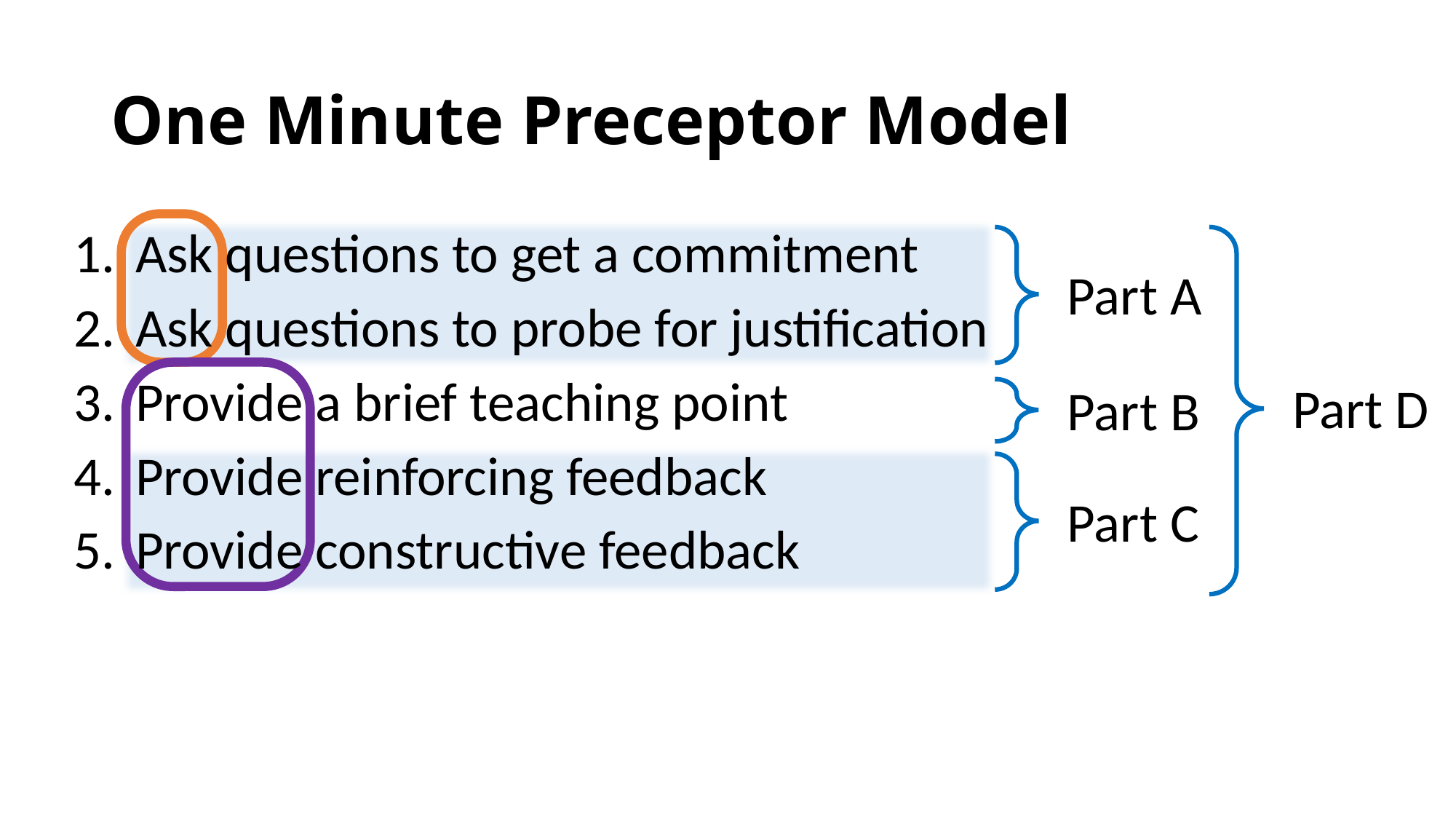

# One Minute Preceptor Model
Ask questions to get a commitment
Ask questions to probe for justification
Provide a brief teaching point
Provide reinforcing feedback
Provide constructive feedback
Part A
Part D
Part B
Part C

## Slide 10
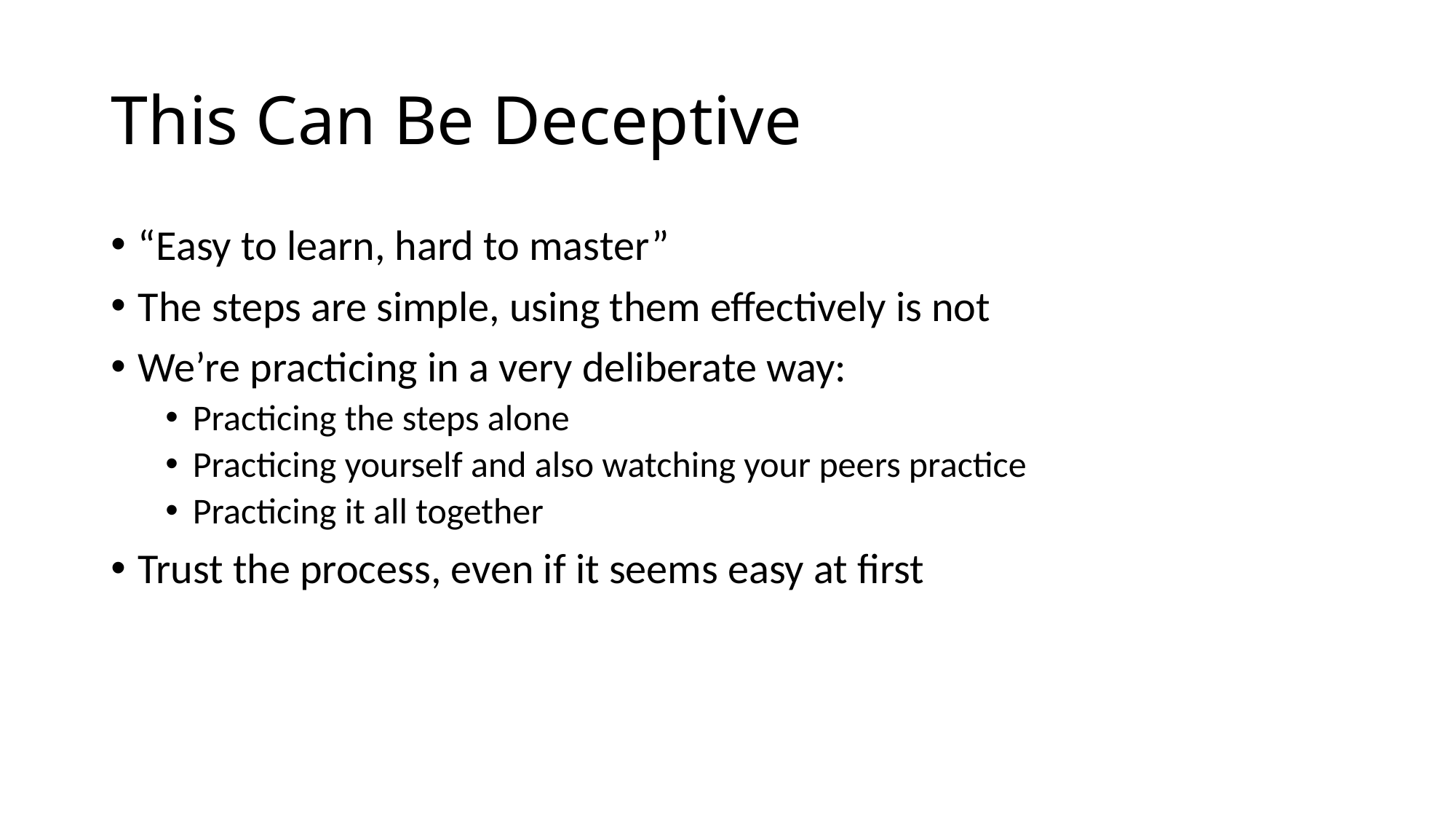

# This Can Be Deceptive
“Easy to learn, hard to master”
The steps are simple, using them effectively is not
We’re practicing in a very deliberate way:
Practicing the steps alone
Practicing yourself and also watching your peers practice
Practicing it all together
Trust the process, even if it seems easy at first

## Slide 11
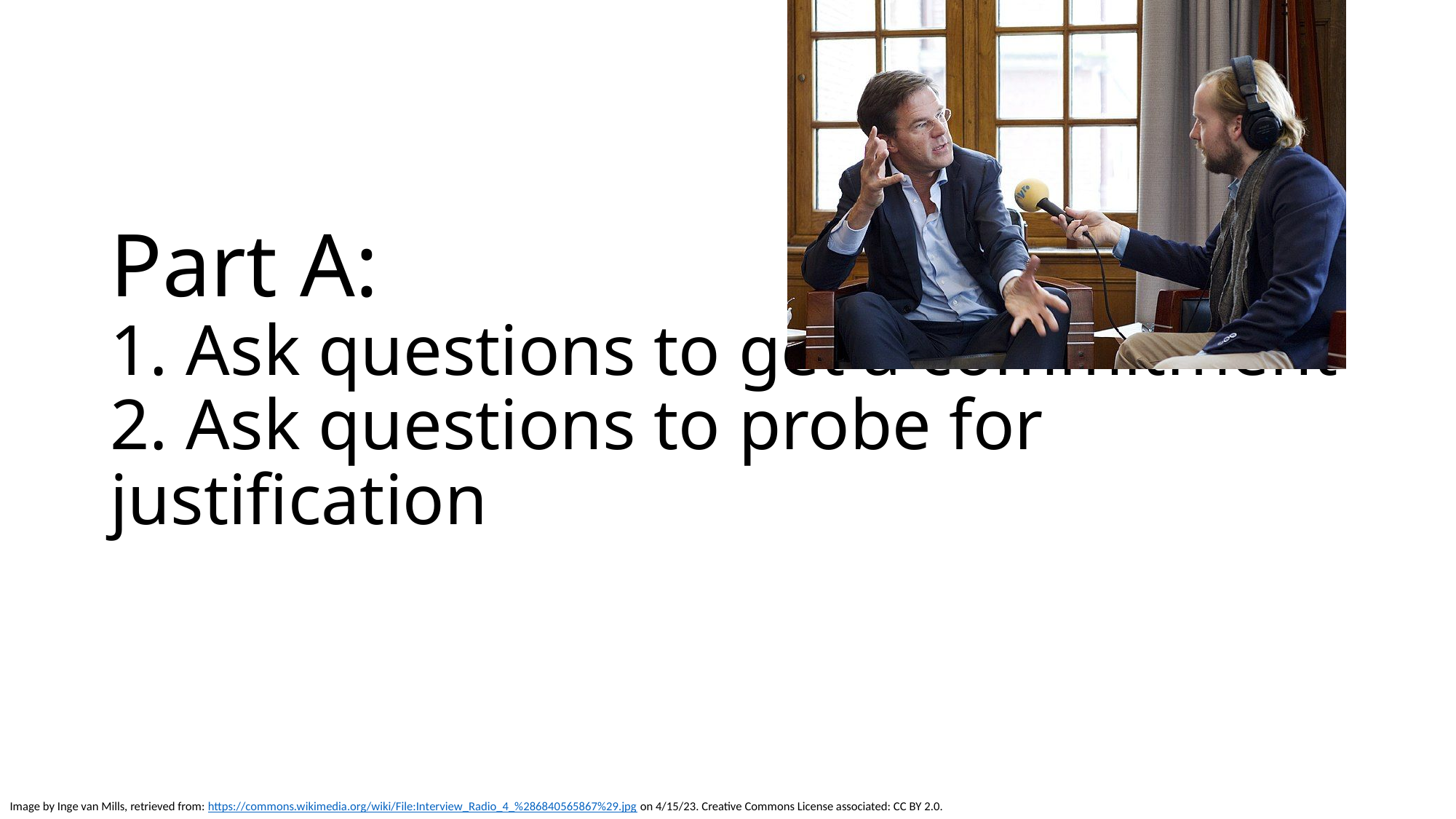

# Part A: 1. Ask questions to get a commitment 2. Ask questions to probe for justification
Image by Inge van Mills, retrieved from: https://commons.wikimedia.org/wiki/File:Interview_Radio_4_%286840565867%29.jpg on 4/15/23. Creative Commons License associated: CC BY 2.0.

## Slide 12
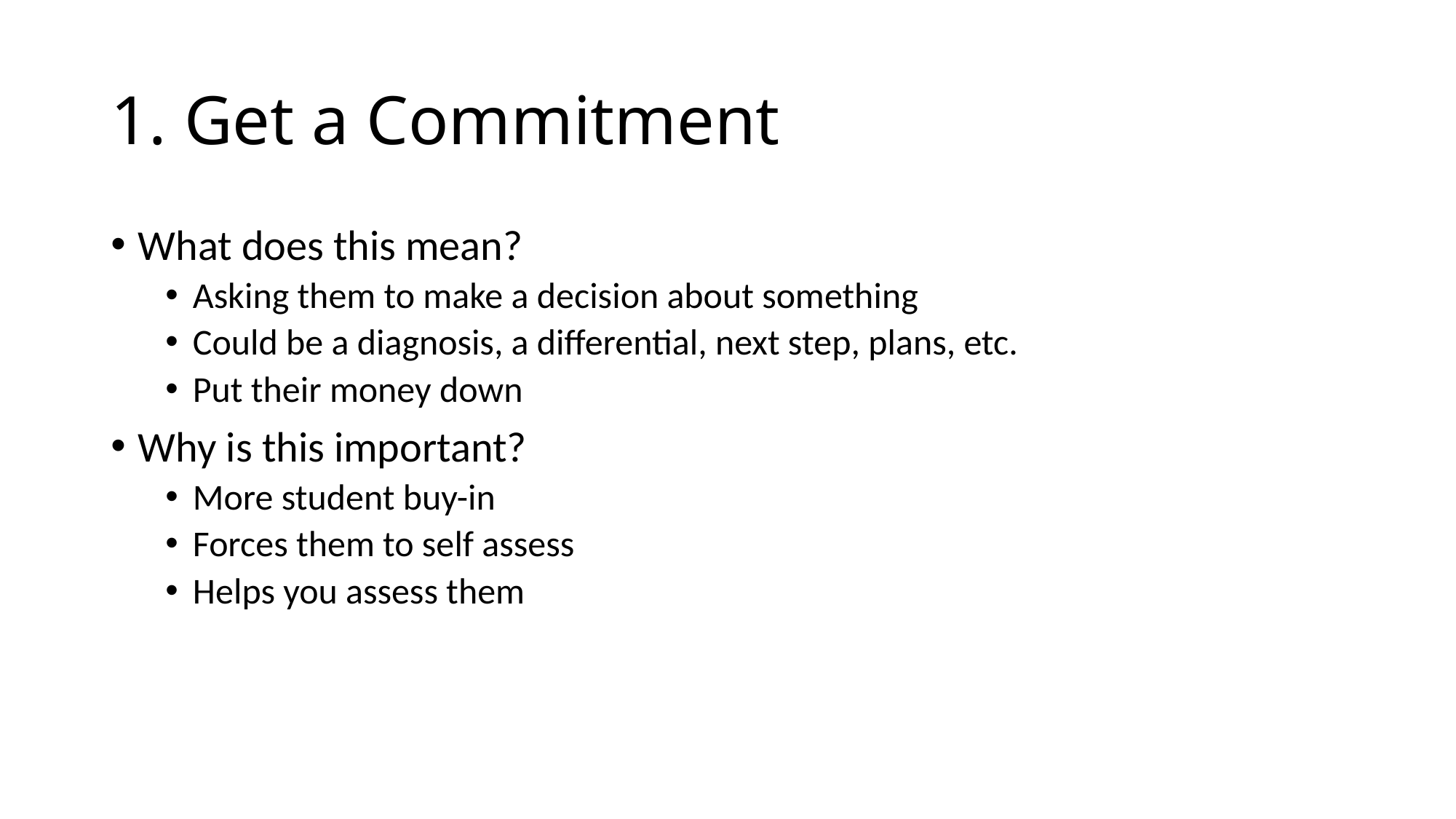

# 1. Get a Commitment
What does this mean?
Asking them to make a decision about something
Could be a diagnosis, a differential, next step, plans, etc.
Put their money down
Why is this important?
More student buy-in
Forces them to self assess
Helps you assess them

## Slide 13
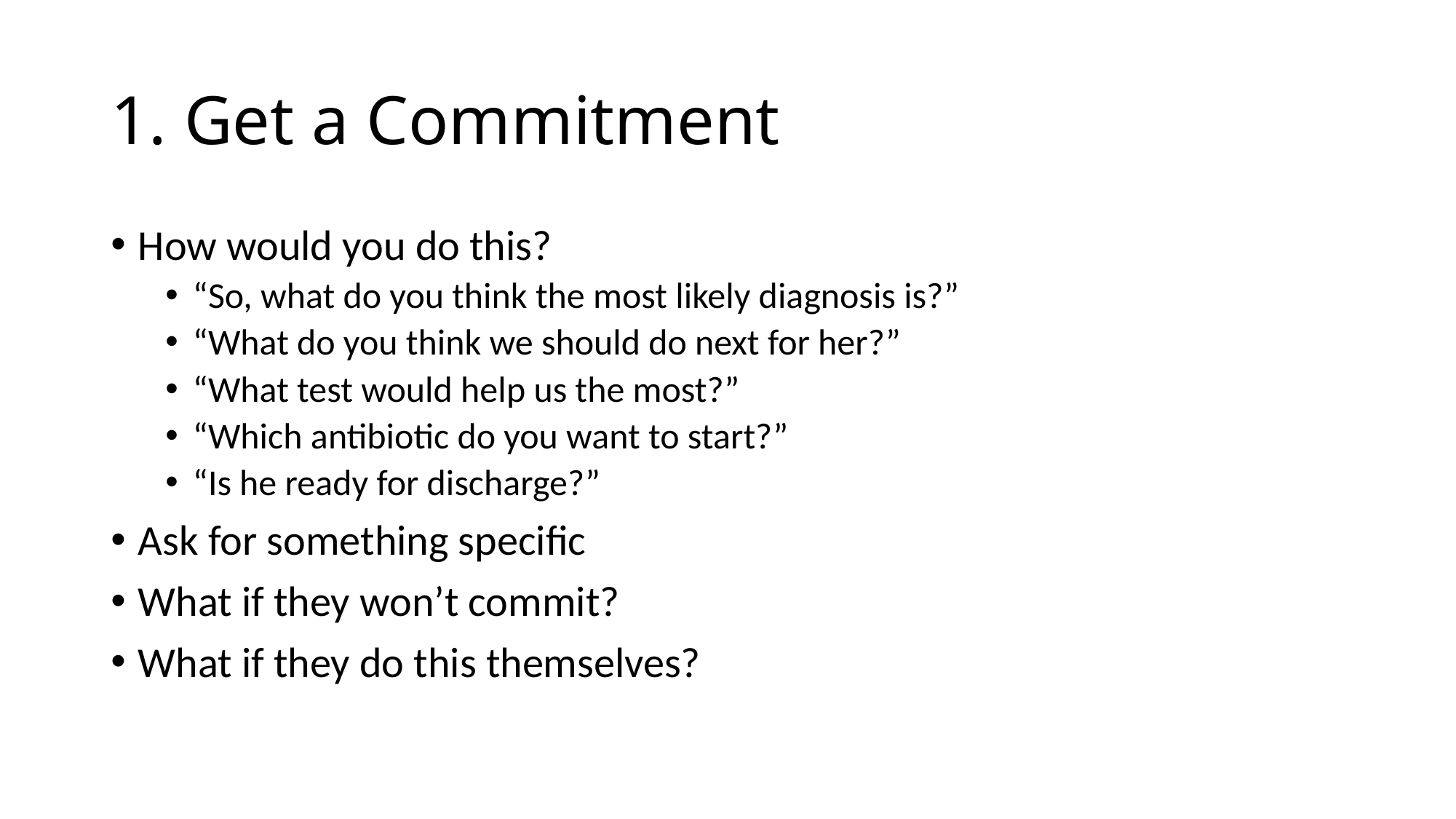

# 1. Get a Commitment
How would you do this?
“So, what do you think the most likely diagnosis is?”
“What do you think we should do next for her?”
“What test would help us the most?”
“Which antibiotic do you want to start?”
“Is he ready for discharge?”
Ask for something specific
What if they won’t commit?
What if they do this themselves?

## Slide 14
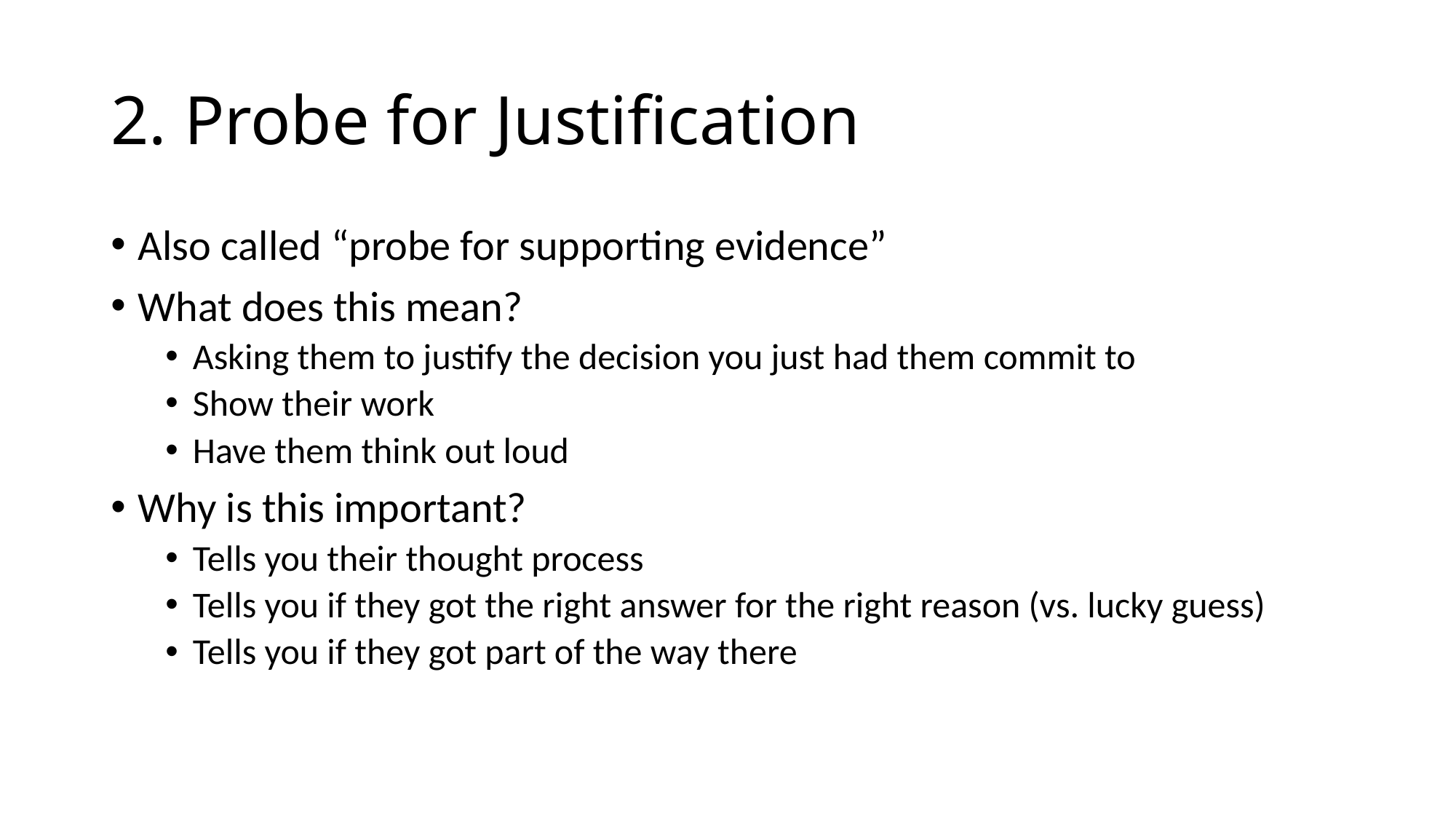

# 2. Probe for Justification
Also called “probe for supporting evidence”
What does this mean?
Asking them to justify the decision you just had them commit to
Show their work
Have them think out loud
Why is this important?
Tells you their thought process
Tells you if they got the right answer for the right reason (vs. lucky guess)
Tells you if they got part of the way there

## Slide 15
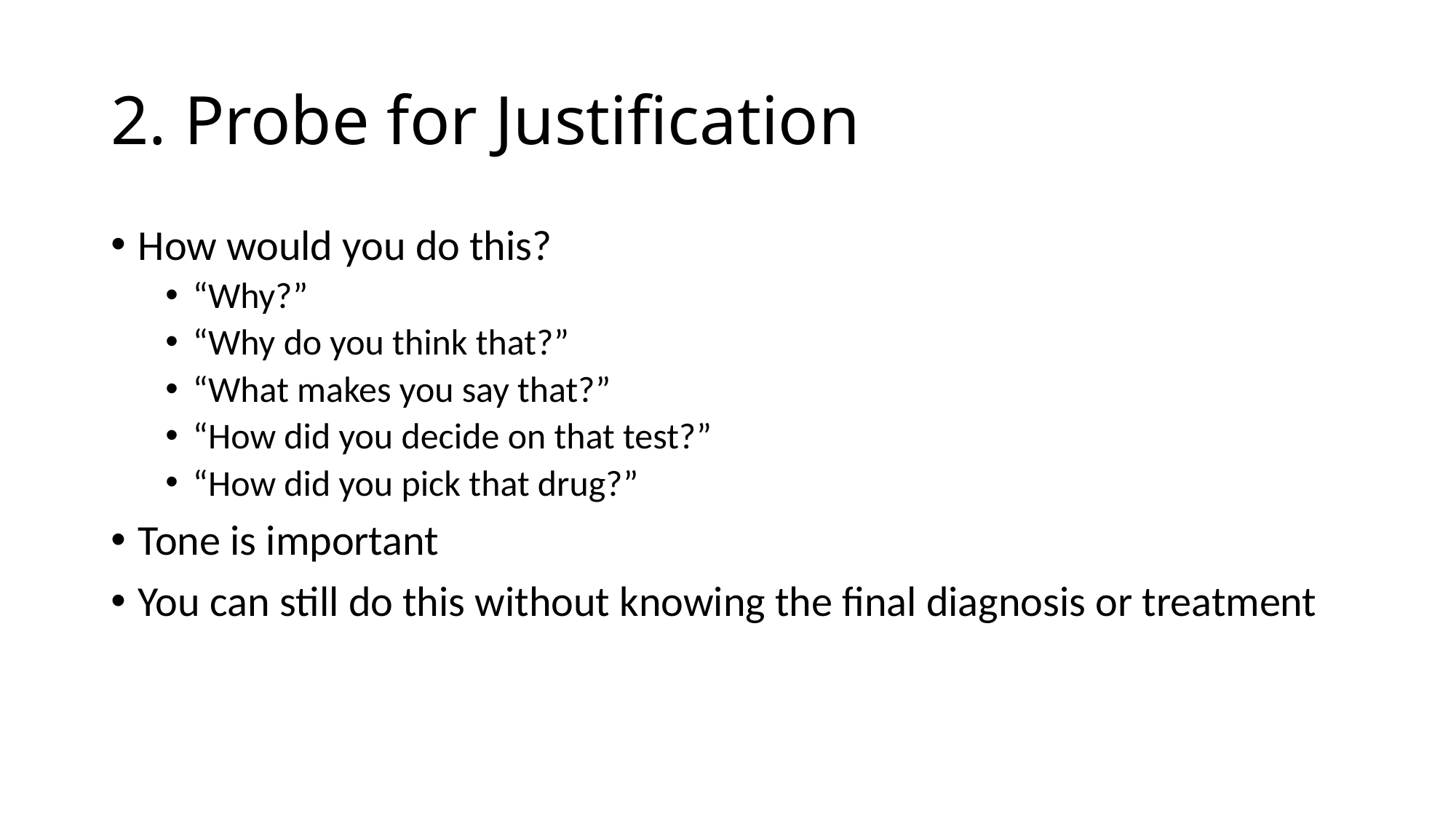

# 2. Probe for Justification
How would you do this?
“Why?”
“Why do you think that?”
“What makes you say that?”
“How did you decide on that test?”
“How did you pick that drug?”
Tone is important
You can still do this without knowing the final diagnosis or treatment

## Slide 16
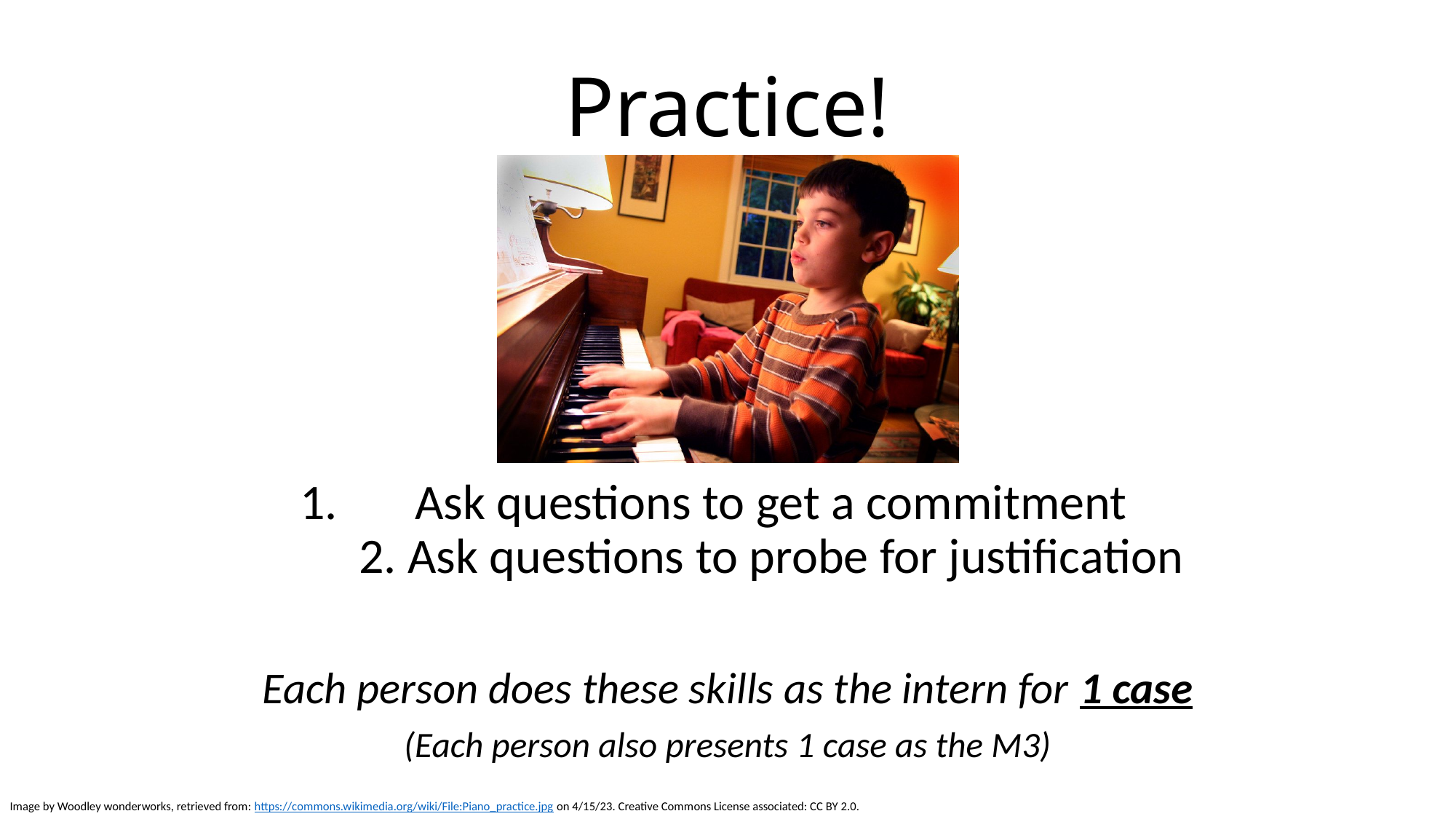

# Practice!
Ask questions to get a commitment
2. Ask questions to probe for justification
Each person does these skills as the intern for 1 case
(Each person also presents 1 case as the M3)
Image by Woodley wonderworks, retrieved from: https://commons.wikimedia.org/wiki/File:Piano_practice.jpg on 4/15/23. Creative Commons License associated: CC BY 2.0.

## Slide 17
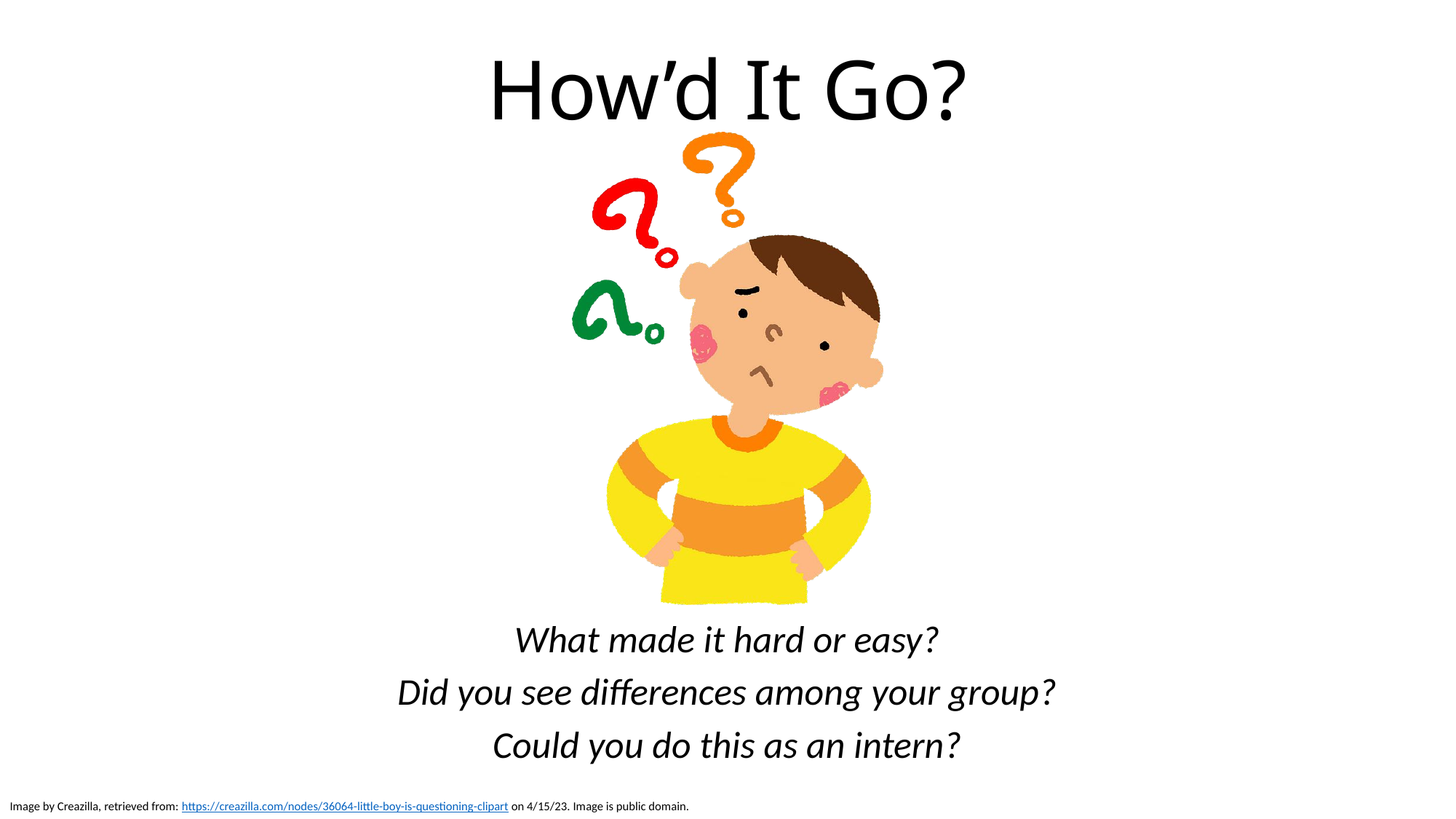

How’d It Go?
What made it hard or easy?
Did you see differences among your group?
Could you do this as an intern?
Image by Creazilla, retrieved from: https://creazilla.com/nodes/36064-little-boy-is-questioning-clipart on 4/15/23. Image is public domain.

## Slide 18
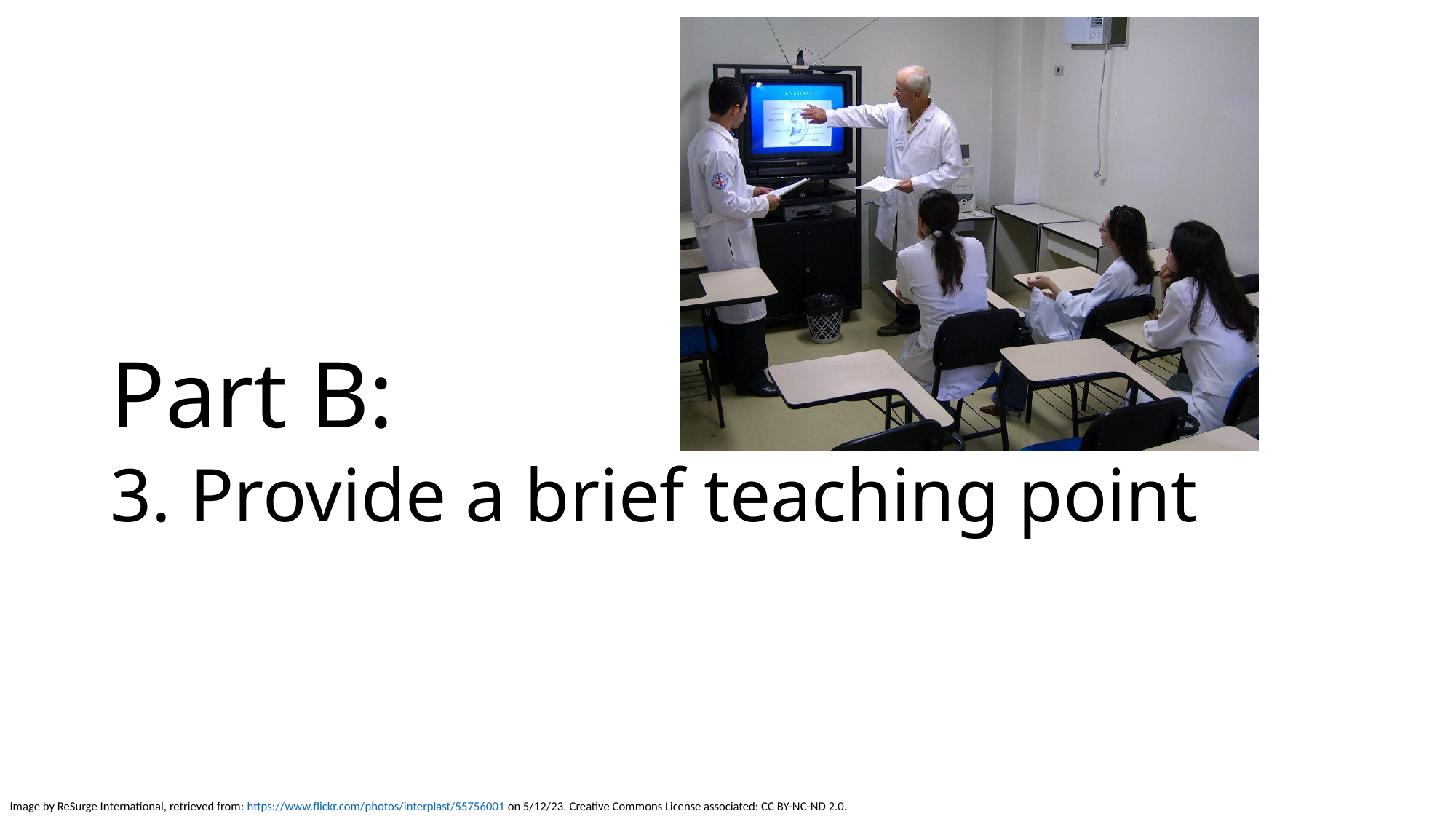

# Part B: 3. Provide a brief teaching point
Image by ReSurge International, retrieved from: https://www.flickr.com/photos/interplast/55756001 on 5/12/23. Creative Commons License associated: CC BY-NC-ND 2.0.

## Slide 19
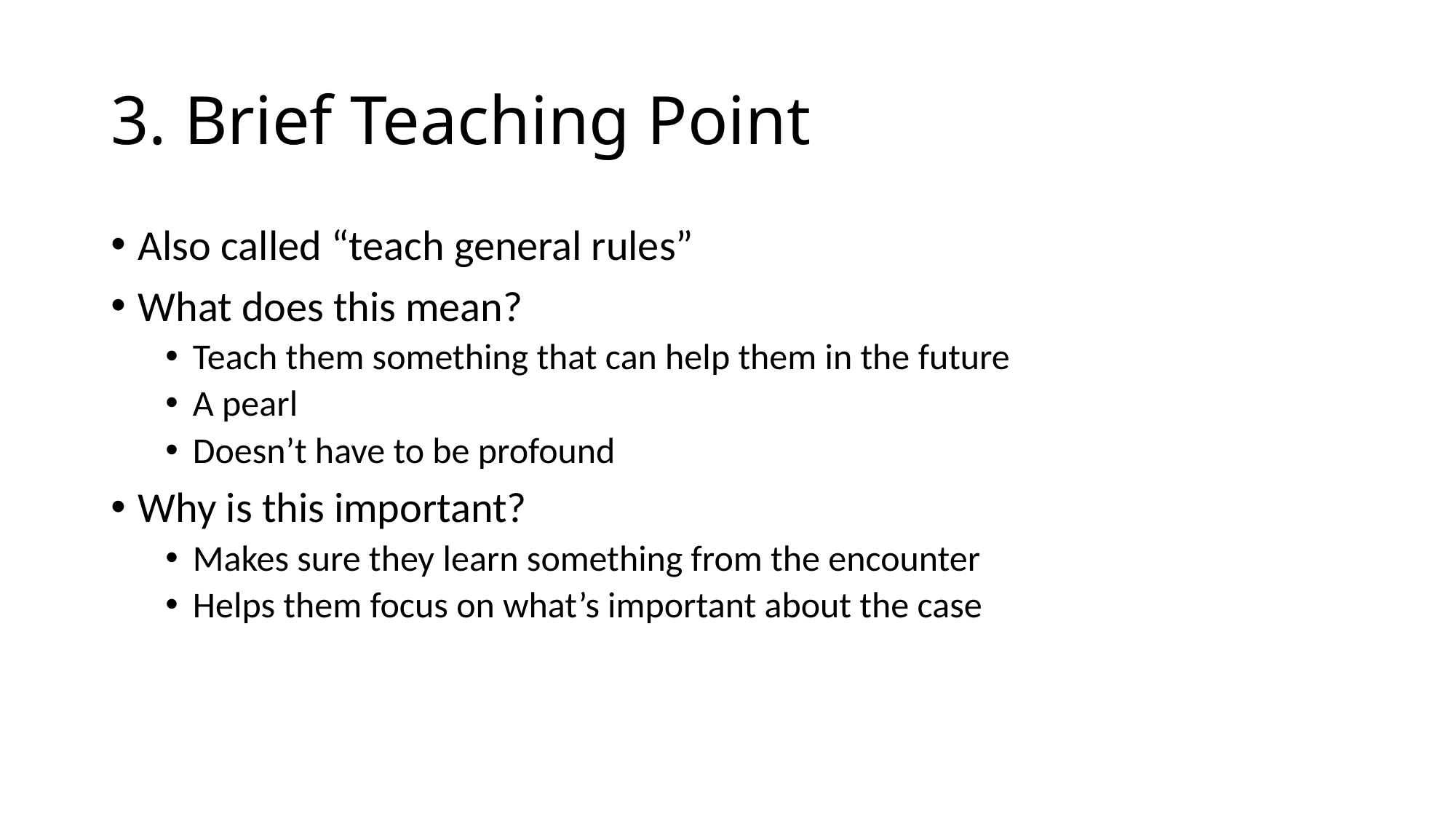

# 3. Brief Teaching Point
Also called “teach general rules”
What does this mean?
Teach them something that can help them in the future
A pearl
Doesn’t have to be profound
Why is this important?
Makes sure they learn something from the encounter
Helps them focus on what’s important about the case

## Slide 20
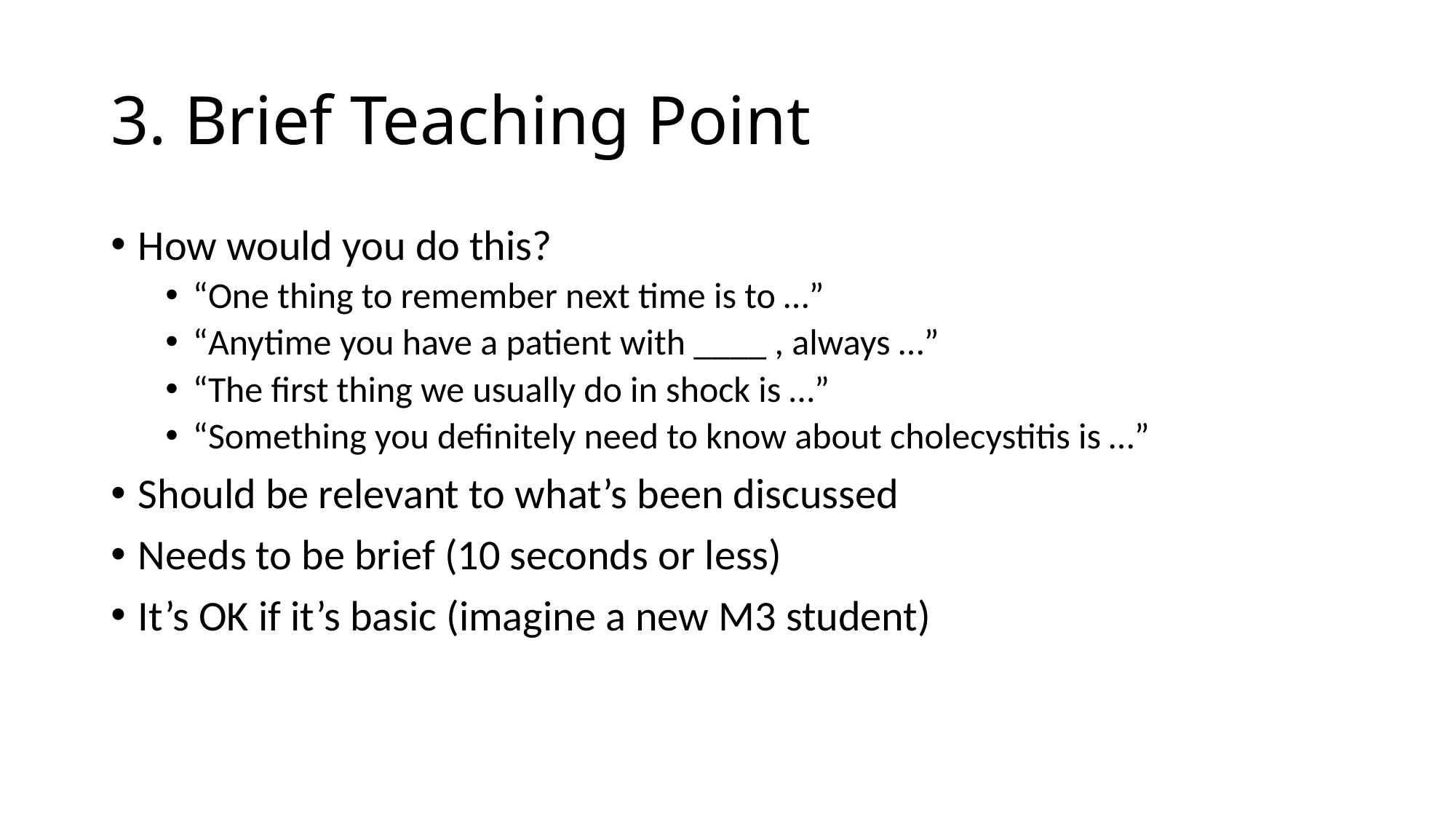

# 3. Brief Teaching Point
How would you do this?
“One thing to remember next time is to …”
“Anytime you have a patient with ____ , always …”
“The first thing we usually do in shock is …”
“Something you definitely need to know about cholecystitis is …”
Should be relevant to what’s been discussed
Needs to be brief (10 seconds or less)
It’s OK if it’s basic (imagine a new M3 student)

## Slide 21
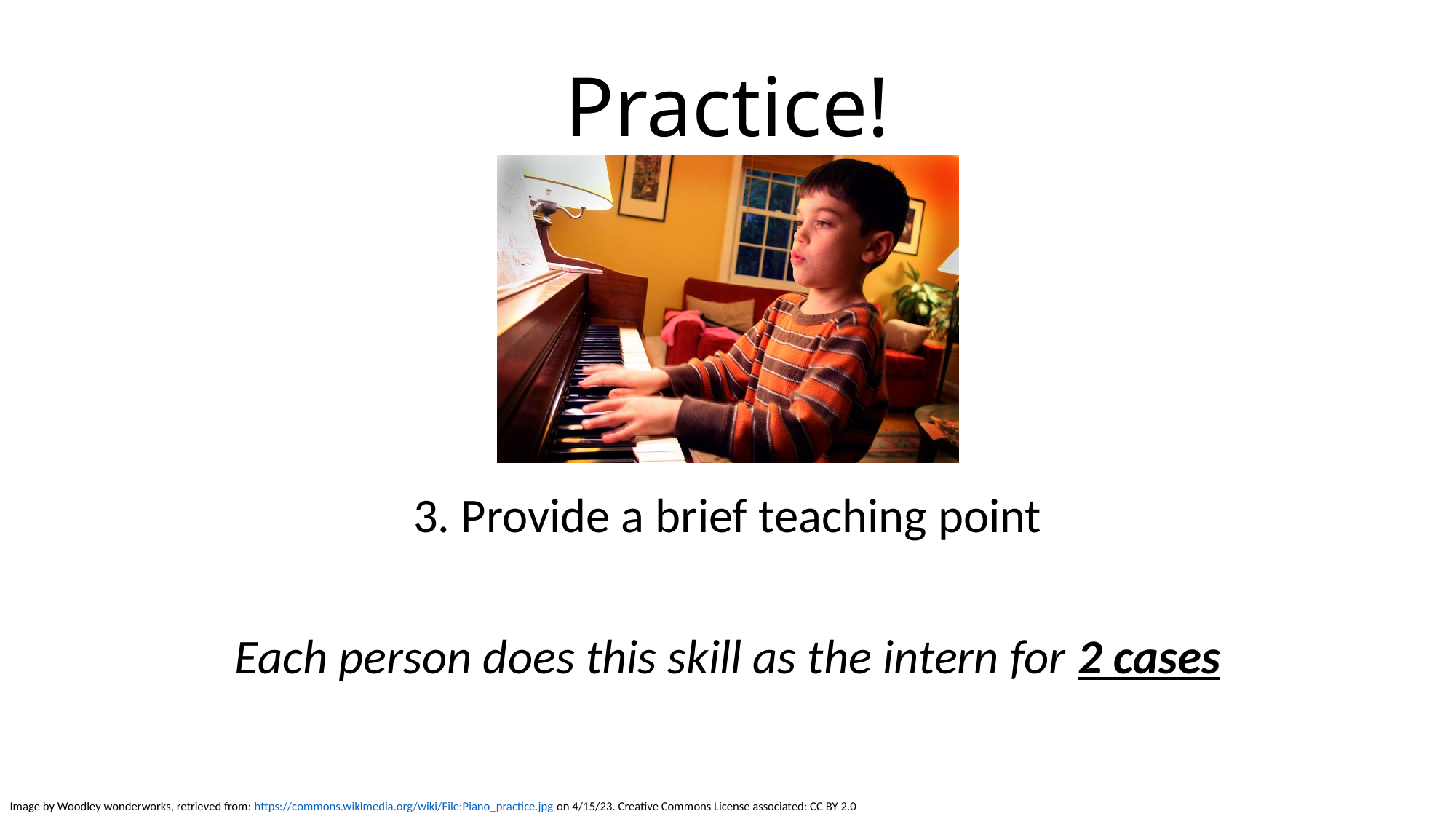

# Practice!
3. Provide a brief teaching point
Each person does this skill as the intern for 2 cases
Image by Woodley wonderworks, retrieved from: https://commons.wikimedia.org/wiki/File:Piano_practice.jpg on 4/15/23. Creative Commons License associated: CC BY 2.0

## Slide 22
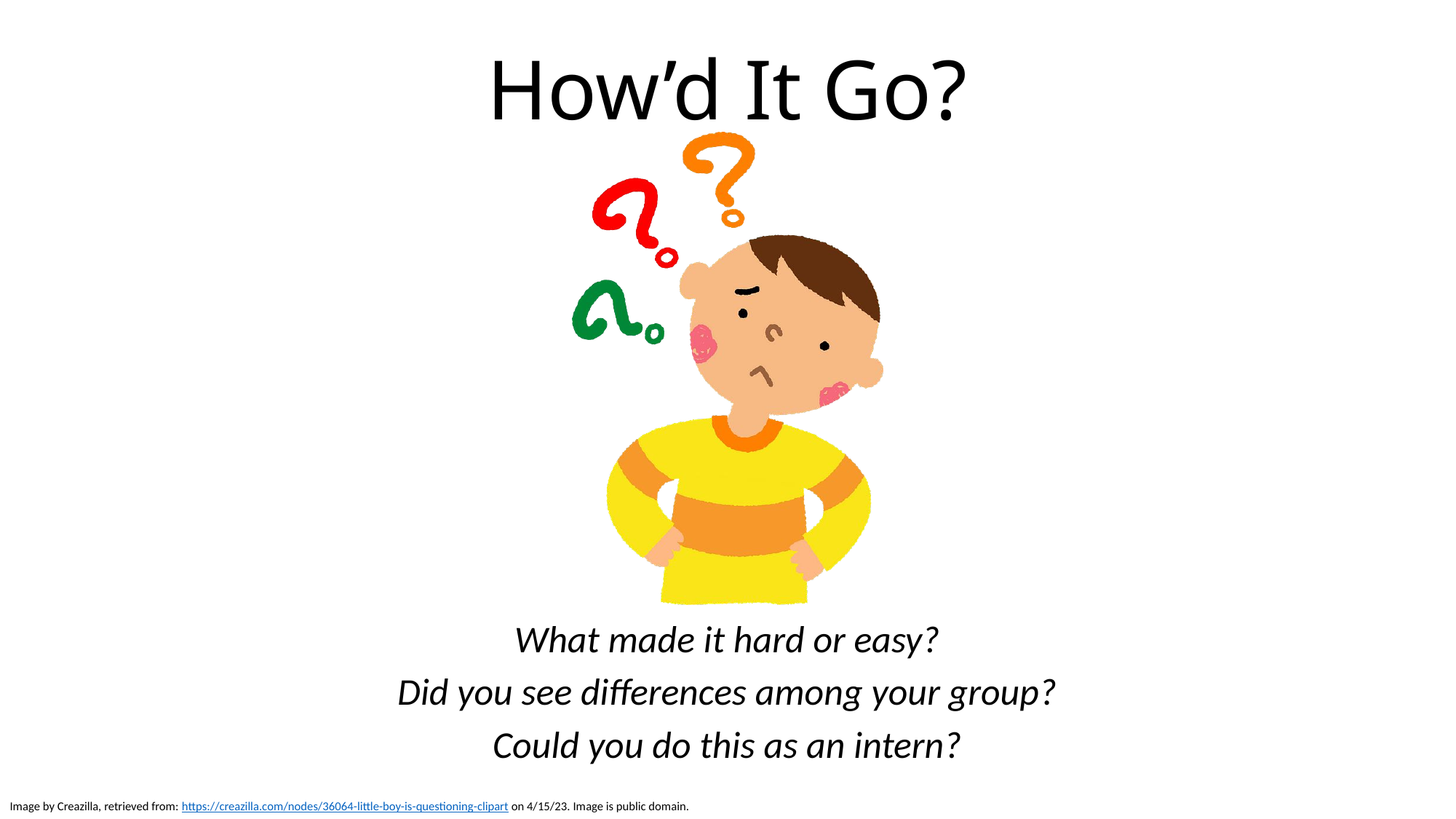

How’d It Go?
What made it hard or easy?
Did you see differences among your group?
Could you do this as an intern?
Image by Creazilla, retrieved from: https://creazilla.com/nodes/36064-little-boy-is-questioning-clipart on 4/15/23. Image is public domain.

## Slide 23
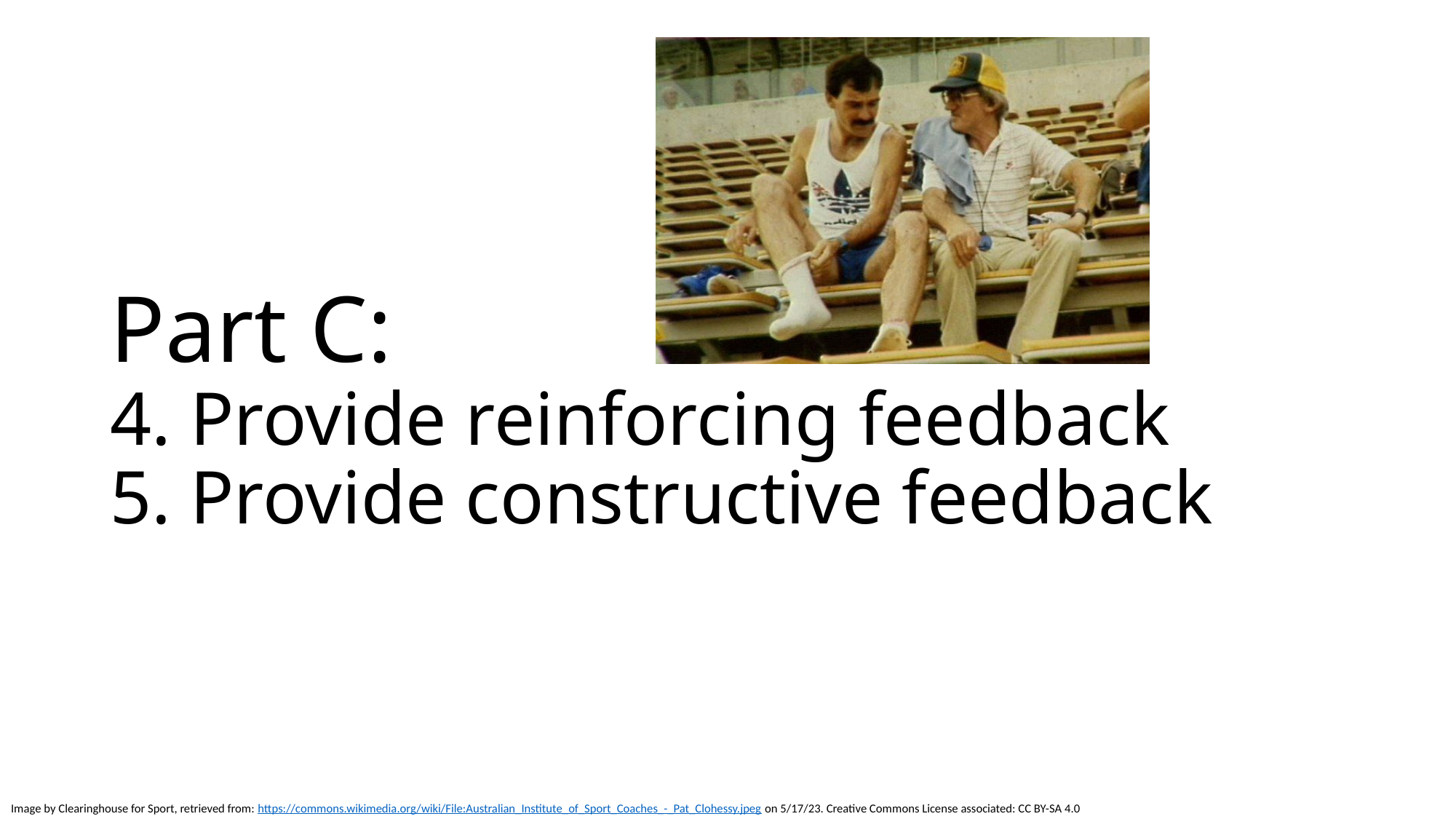

# Part C: 4. Provide reinforcing feedback 5. Provide constructive feedback
Image by Clearinghouse for Sport, retrieved from: https://commons.wikimedia.org/wiki/File:Australian_Institute_of_Sport_Coaches_-_Pat_Clohessy.jpeg on 5/17/23. Creative Commons License associated: CC BY-SA 4.0

## Slide 24
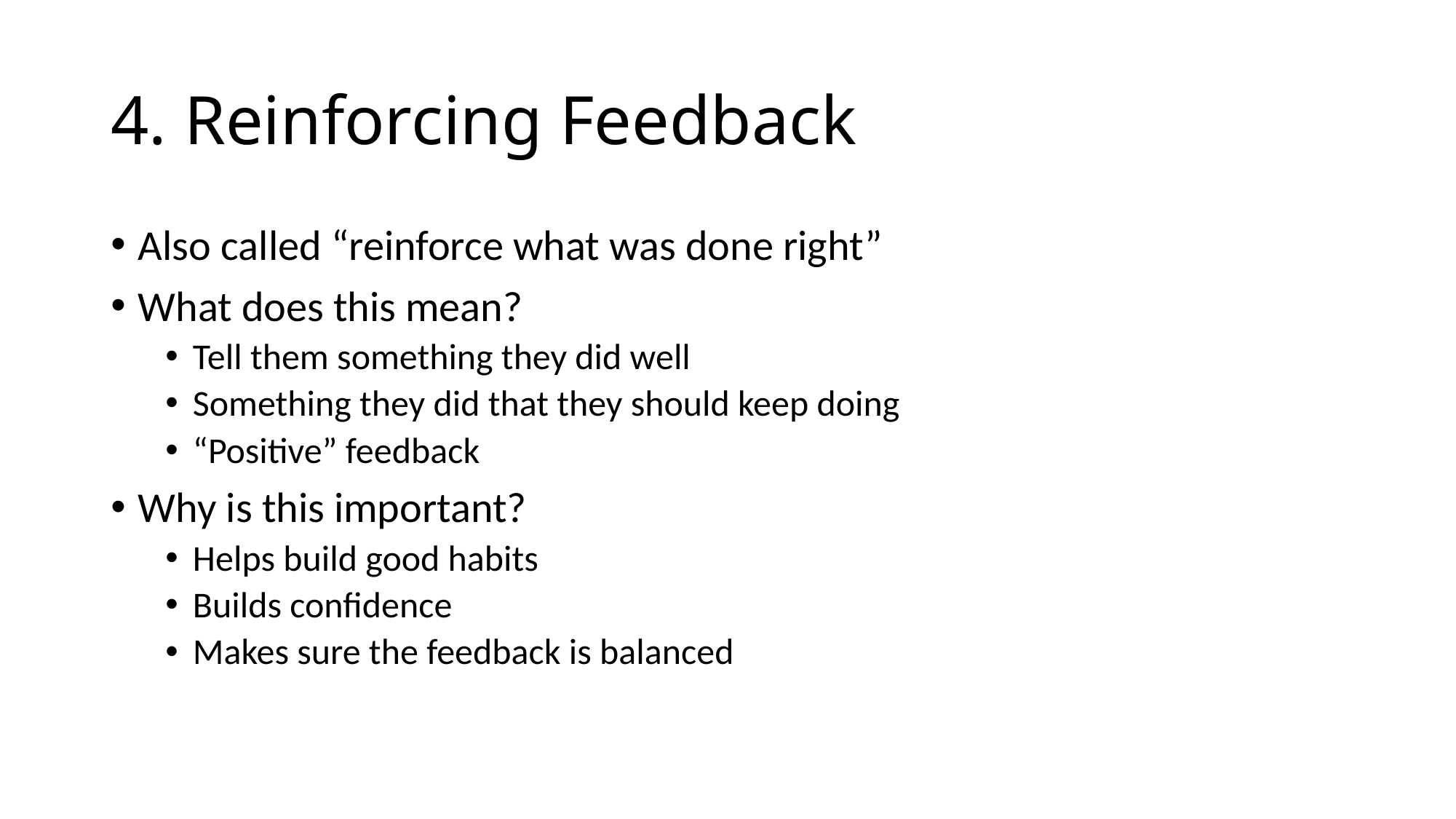

# 4. Reinforcing Feedback
Also called “reinforce what was done right”
What does this mean?
Tell them something they did well
Something they did that they should keep doing
“Positive” feedback
Why is this important?
Helps build good habits
Builds confidence
Makes sure the feedback is balanced

## Slide 25
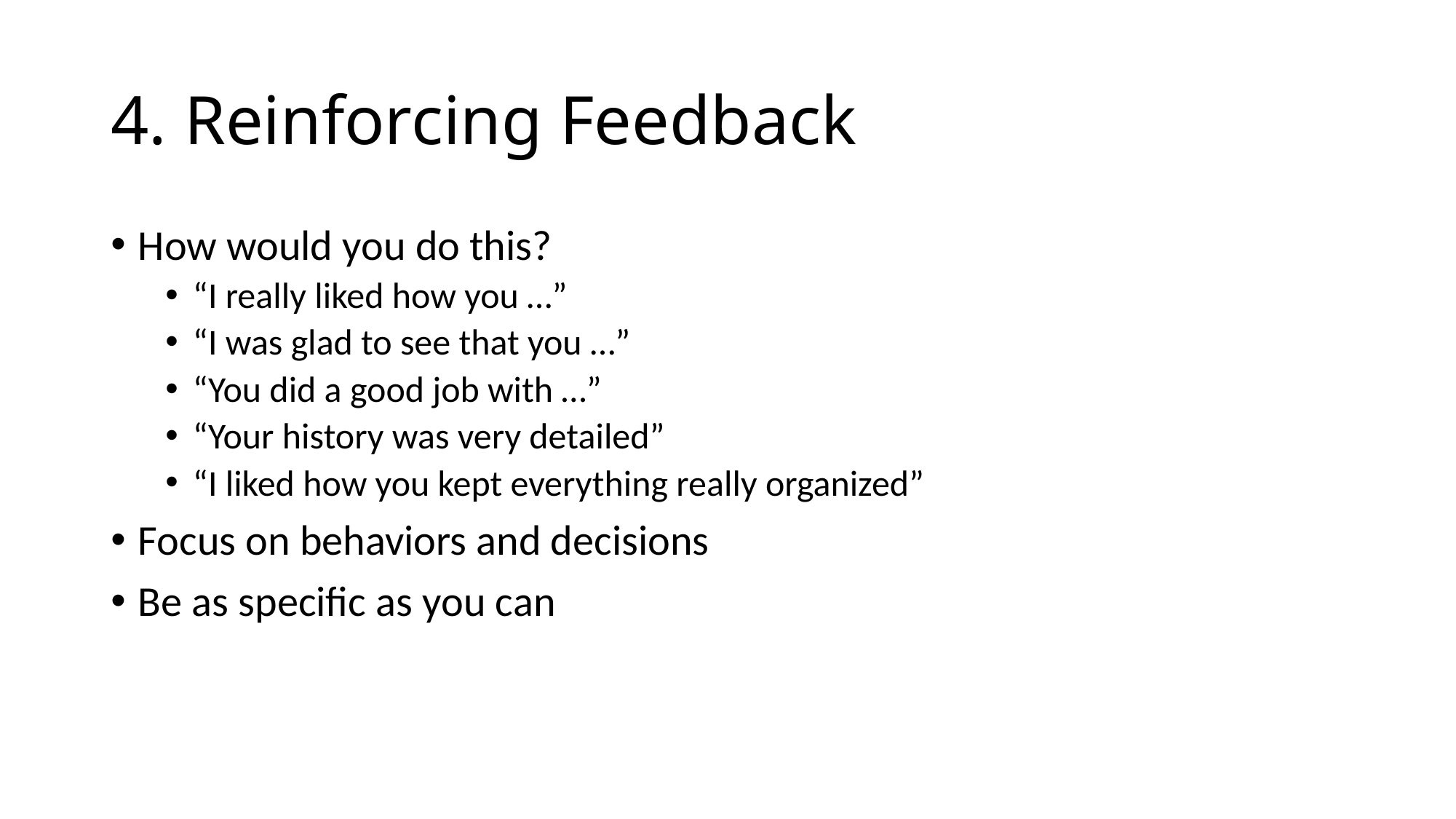

# 4. Reinforcing Feedback
How would you do this?
“I really liked how you …”
“I was glad to see that you …”
“You did a good job with …”
“Your history was very detailed”
“I liked how you kept everything really organized”
Focus on behaviors and decisions
Be as specific as you can

## Slide 26
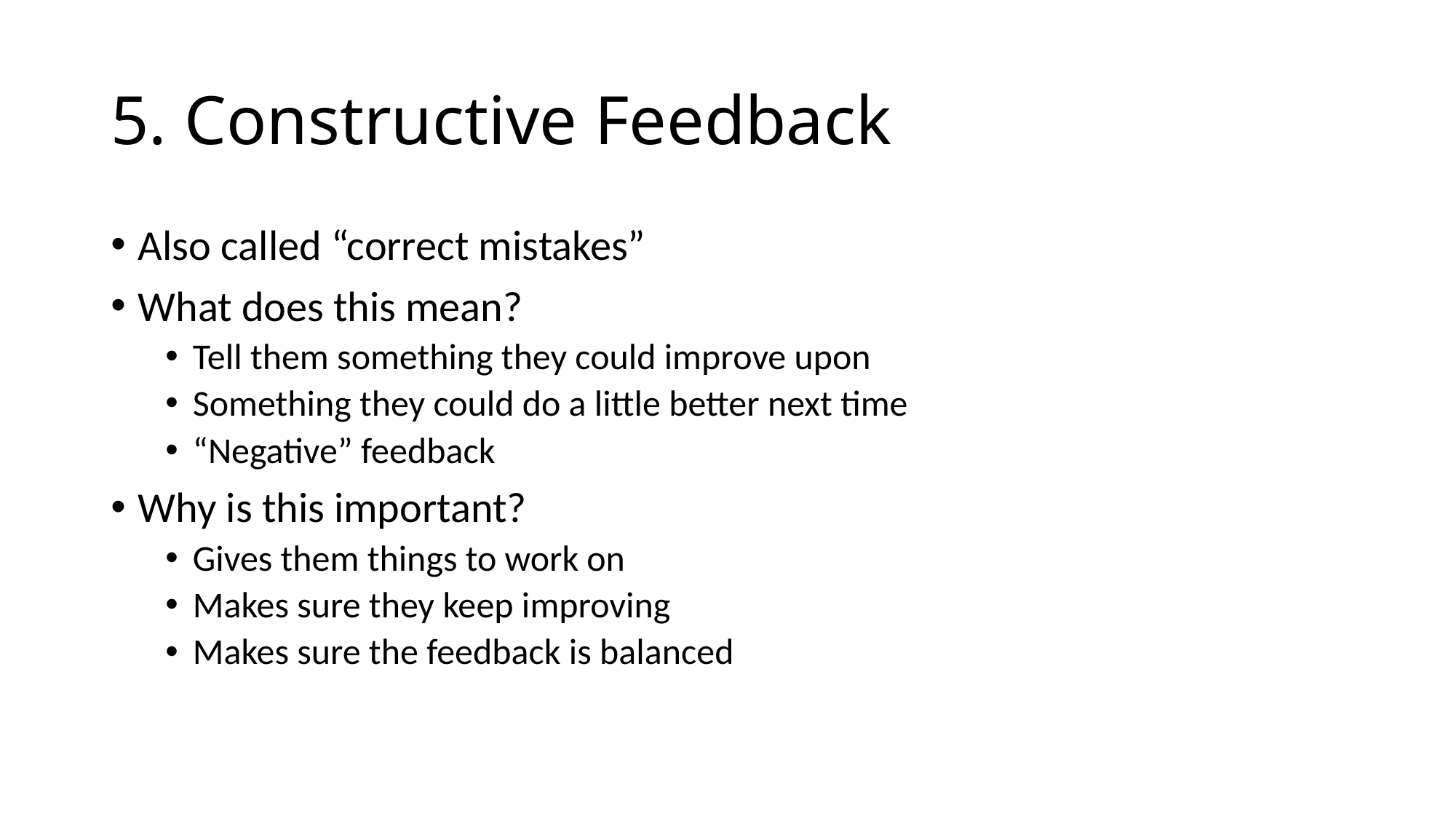

# 5. Constructive Feedback
Also called “correct mistakes”
What does this mean?
Tell them something they could improve upon
Something they could do a little better next time
“Negative” feedback
Why is this important?
Gives them things to work on
Makes sure they keep improving
Makes sure the feedback is balanced

## Slide 27
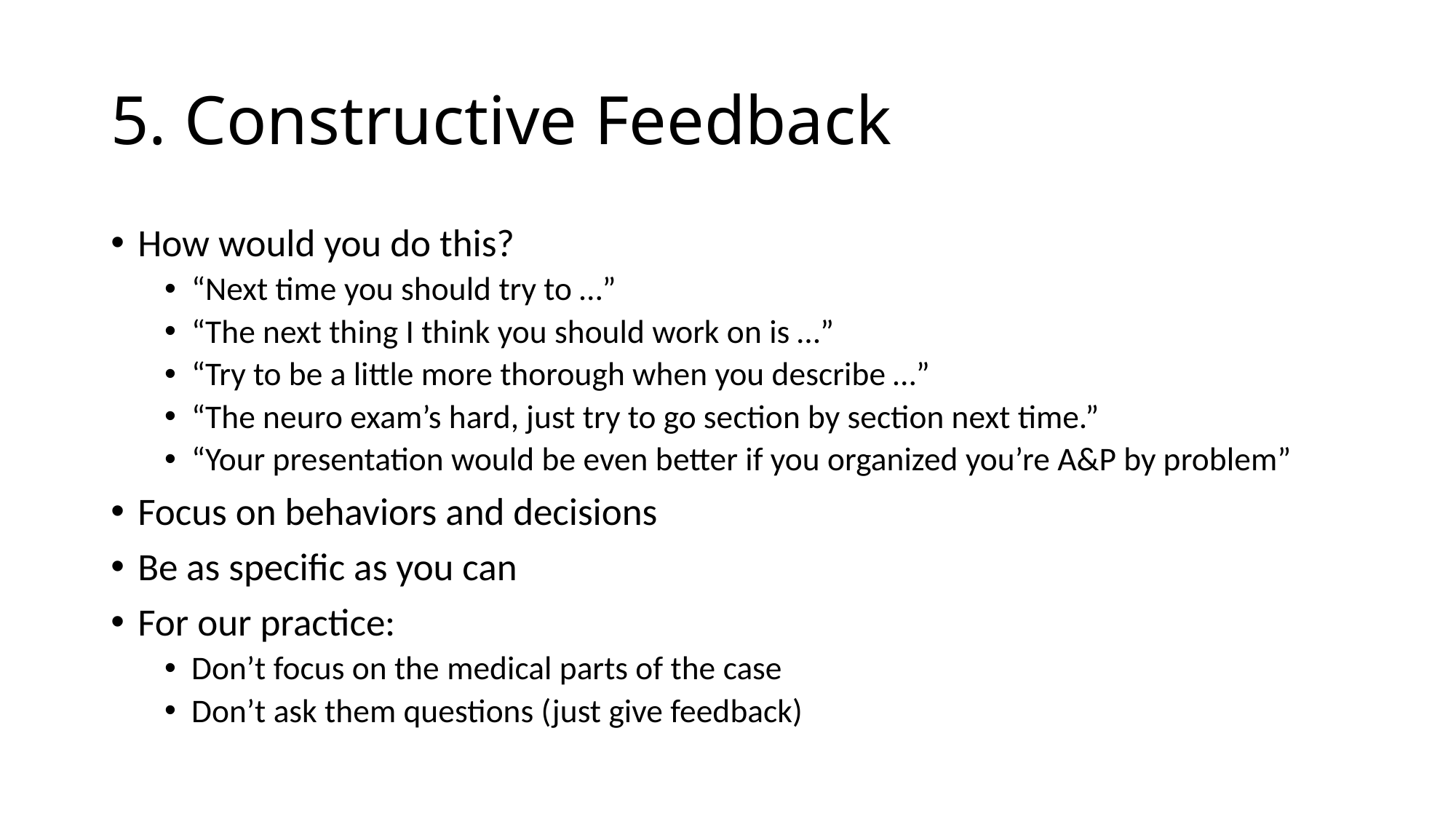

# 5. Constructive Feedback
How would you do this?
“Next time you should try to …”
“The next thing I think you should work on is …”
“Try to be a little more thorough when you describe …”
“The neuro exam’s hard, just try to go section by section next time.”
“Your presentation would be even better if you organized you’re A&P by problem”
Focus on behaviors and decisions
Be as specific as you can
For our practice:
Don’t focus on the medical parts of the case
Don’t ask them questions (just give feedback)

## Slide 28
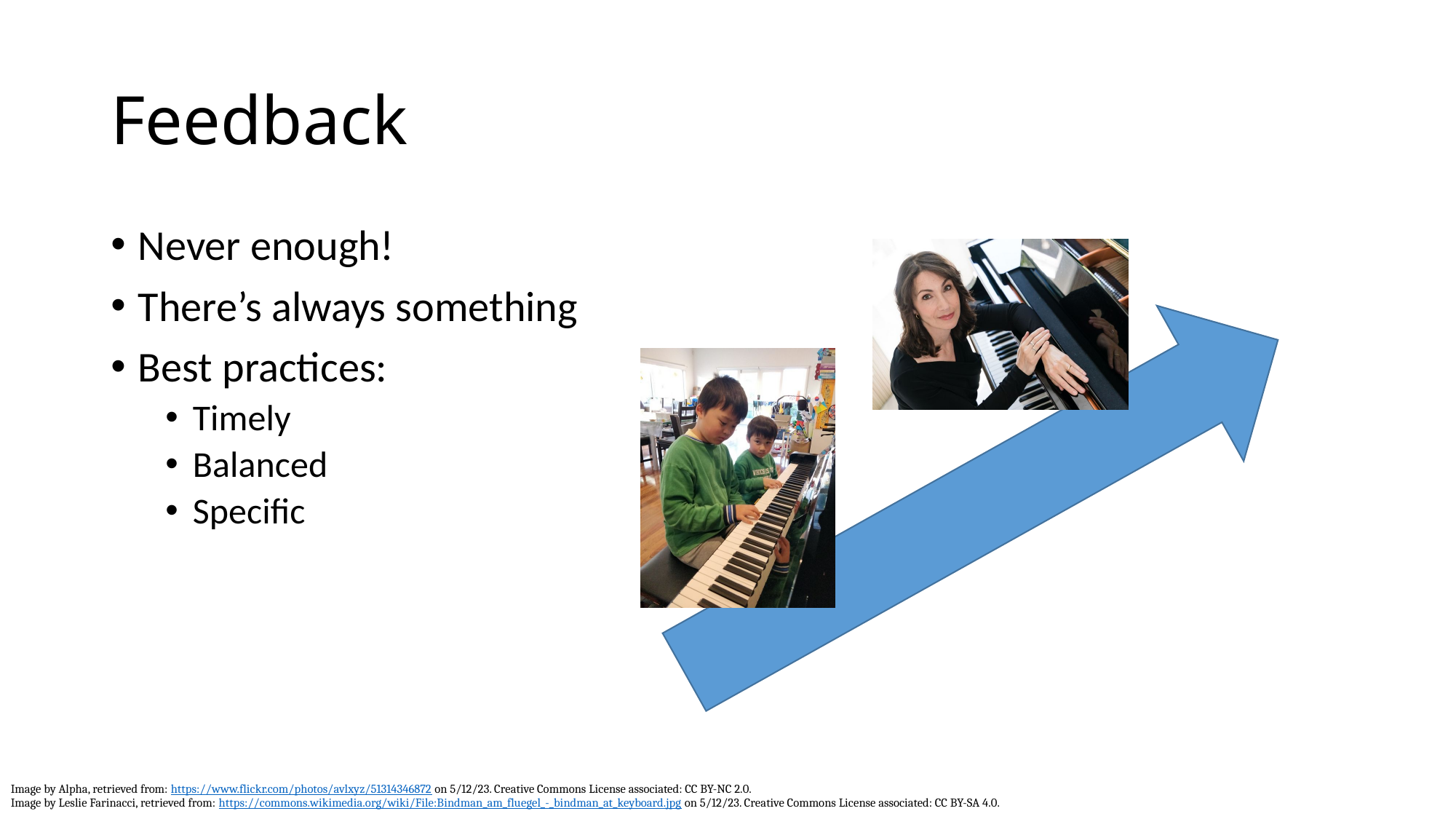

# Feedback
Never enough!
There’s always something
Best practices:
Timely
Balanced
Specific
Image by Alpha, retrieved from: https://www.flickr.com/photos/avlxyz/51314346872 on 5/12/23. Creative Commons License associated: CC BY-NC 2.0.
Image by Leslie Farinacci, retrieved from: https://commons.wikimedia.org/wiki/File:Bindman_am_fluegel_-_bindman_at_keyboard.jpg on 5/12/23. Creative Commons License associated: CC BY-SA 4.0.

## Slide 29
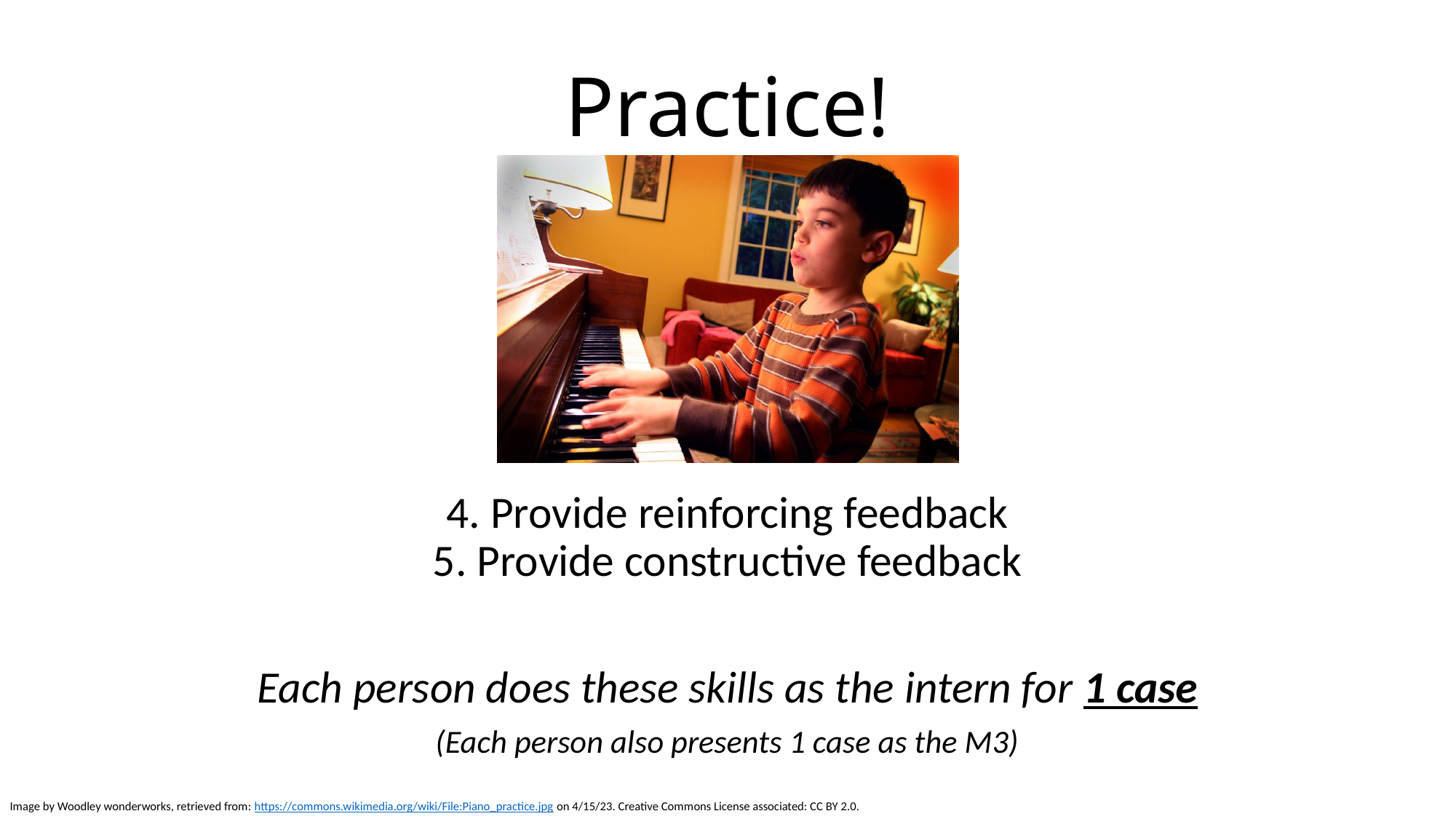

# Practice!
4. Provide reinforcing feedback
5. Provide constructive feedback
Each person does these skills as the intern for 1 case
(Each person also presents 1 case as the M3)
Image by Woodley wonderworks, retrieved from: https://commons.wikimedia.org/wiki/File:Piano_practice.jpg on 4/15/23. Creative Commons License associated: CC BY 2.0.

## Slide 30
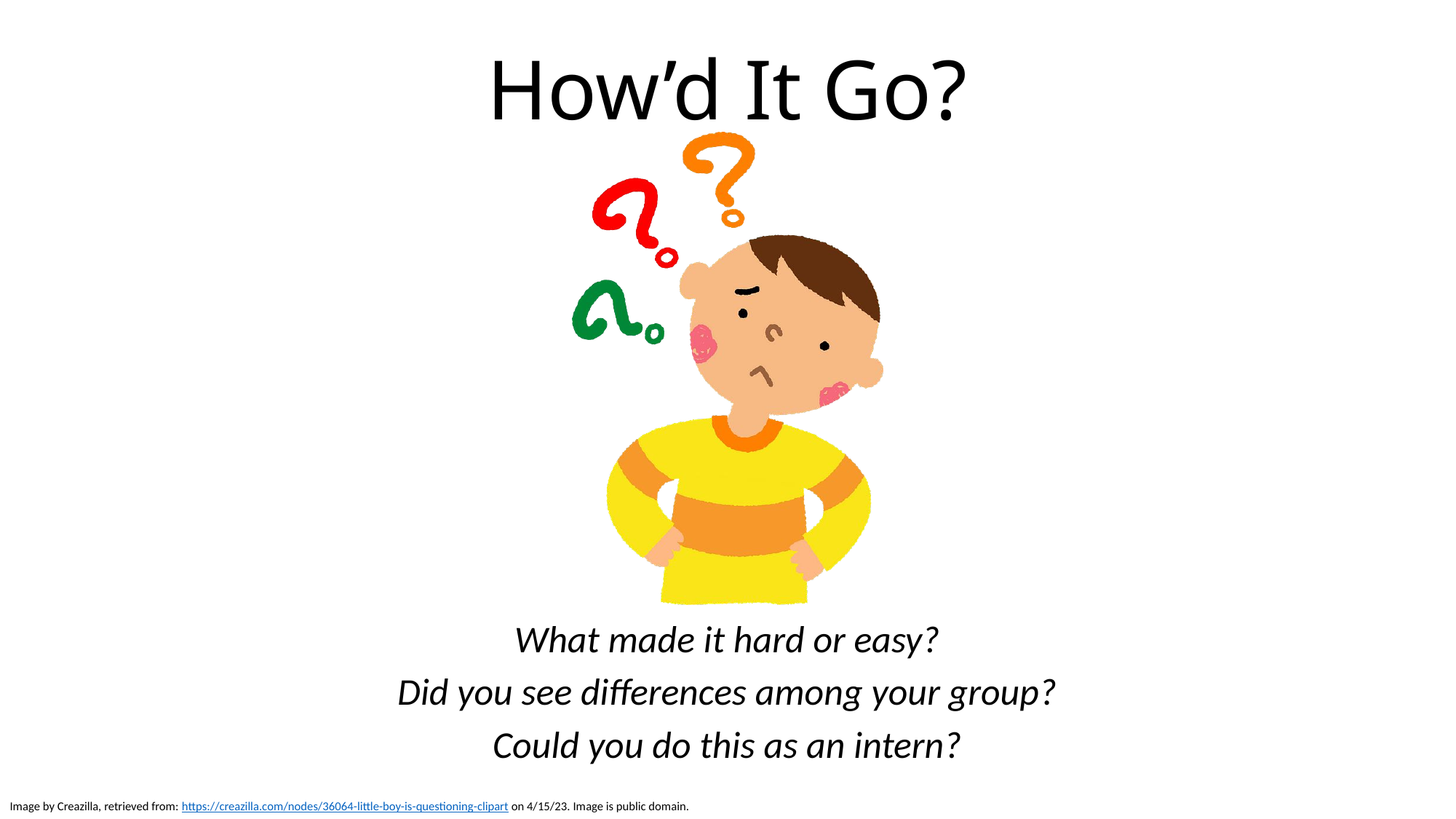

How’d It Go?
What made it hard or easy?
Did you see differences among your group?
Could you do this as an intern?
Image by Creazilla, retrieved from: https://creazilla.com/nodes/36064-little-boy-is-questioning-clipart on 4/15/23. Image is public domain.

## Slide 31
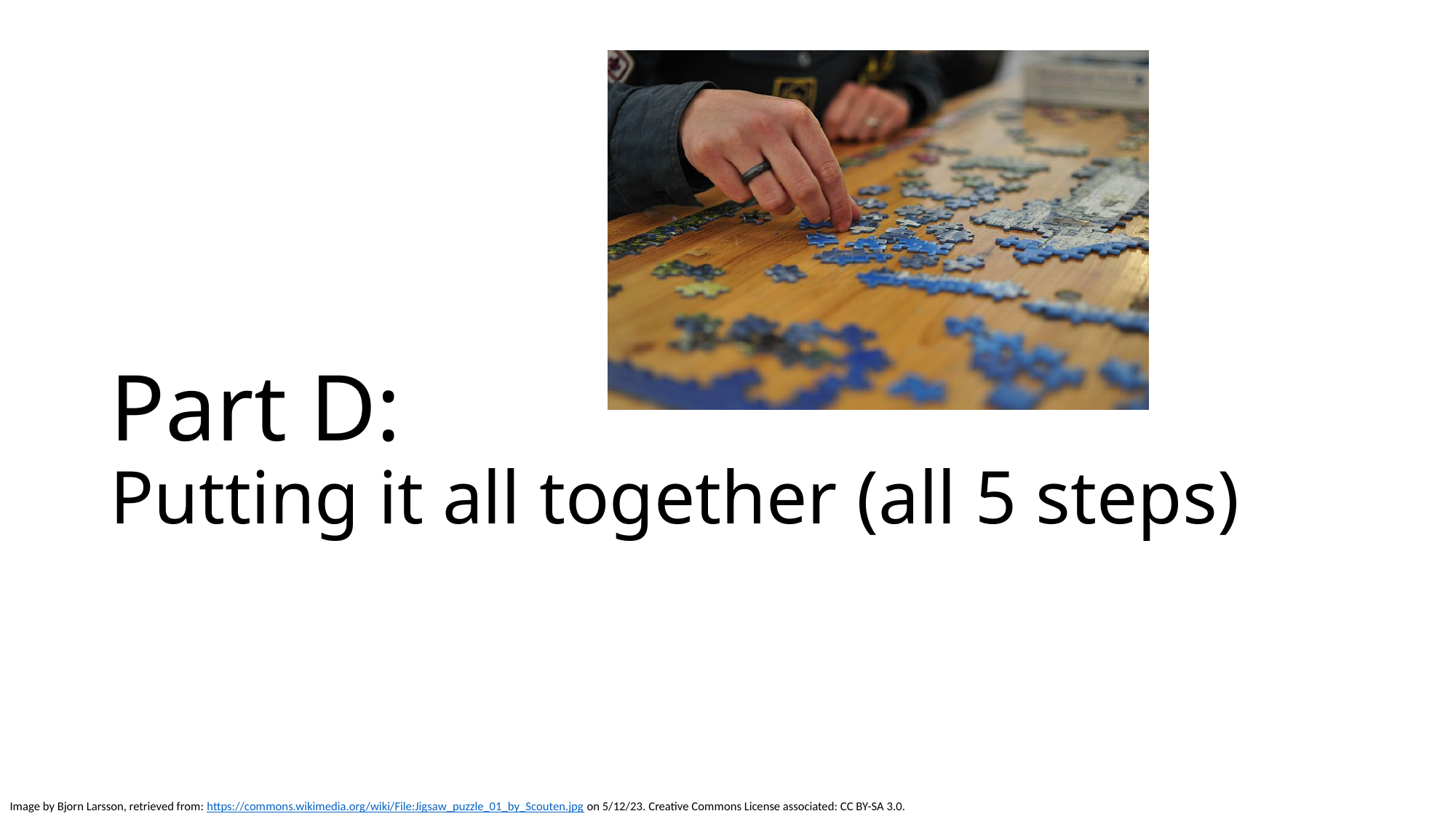

# Part D: Putting it all together (all 5 steps)
Image by Bjorn Larsson, retrieved from: https://commons.wikimedia.org/wiki/File:Jigsaw_puzzle_01_by_Scouten.jpg on 5/12/23. Creative Commons License associated: CC BY-SA 3.0.

## Slide 32
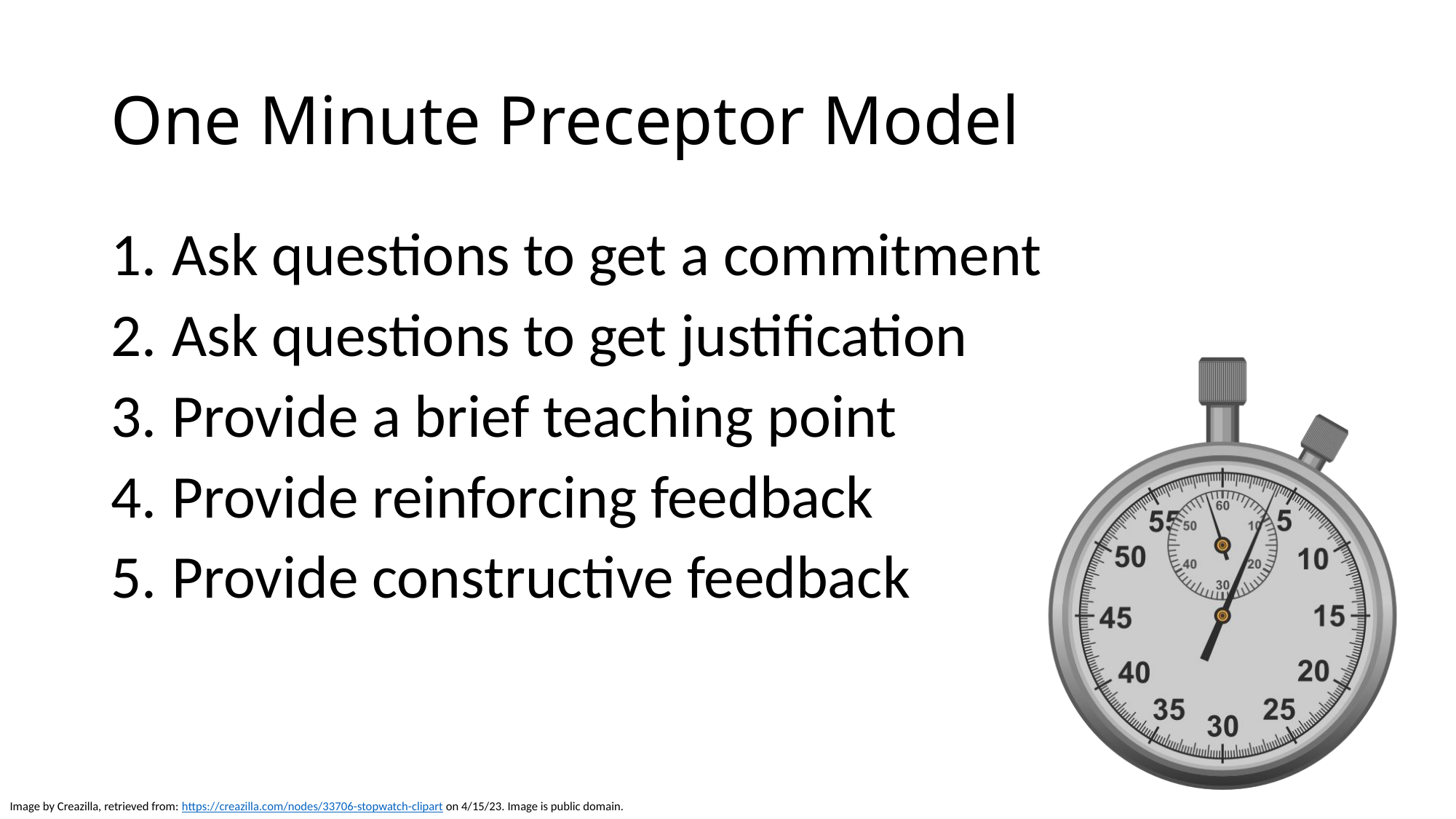

# One Minute Preceptor Model
Ask questions to get a commitment
Ask questions to get justification
Provide a brief teaching point
Provide reinforcing feedback
Provide constructive feedback
Image by Creazilla, retrieved from: https://creazilla.com/nodes/33706-stopwatch-clipart on 4/15/23. Image is public domain.

## Slide 33
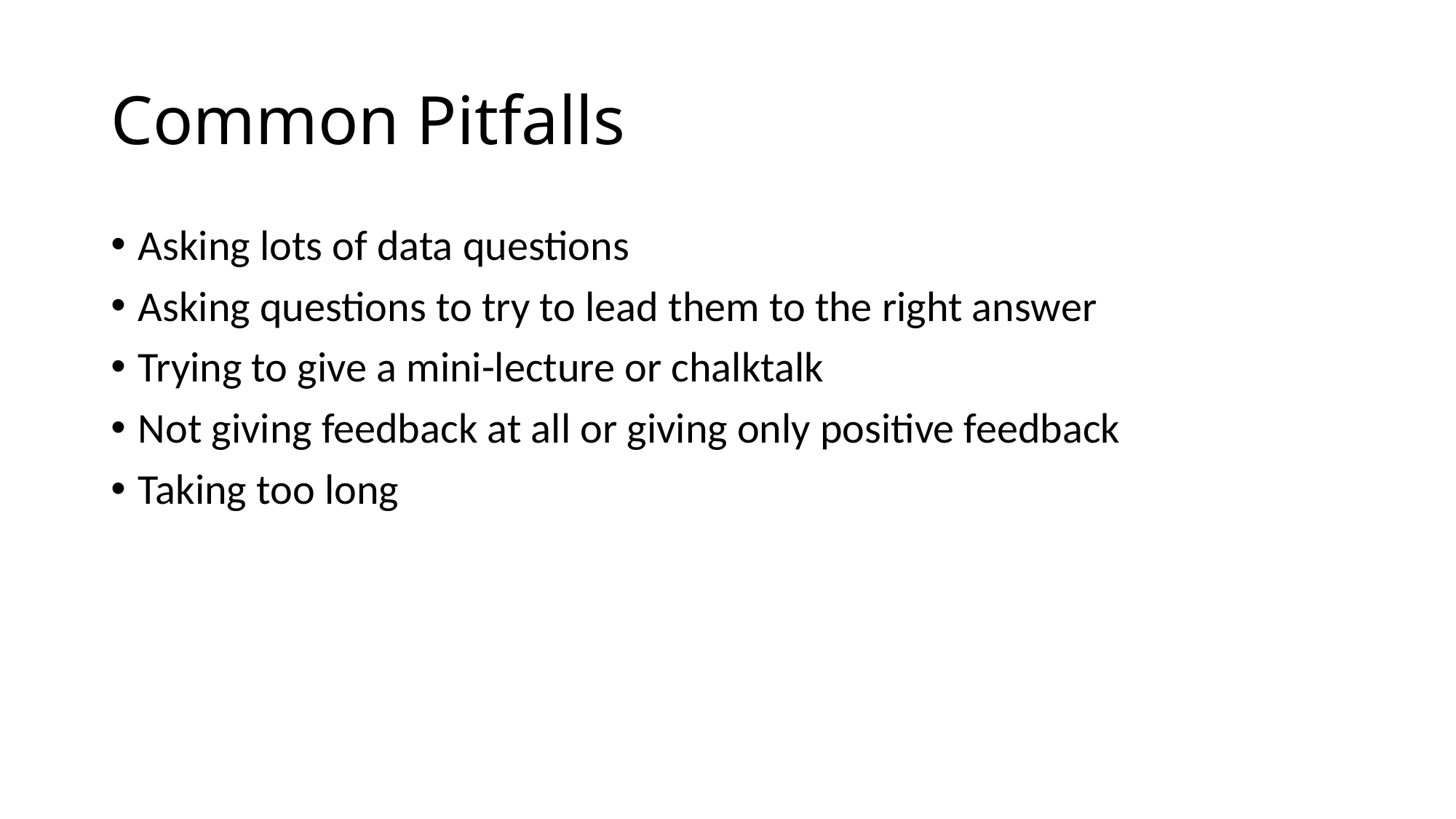

# Common Pitfalls
Asking lots of data questions
Asking questions to try to lead them to the right answer
Trying to give a mini-lecture or chalktalk
Not giving feedback at all or giving only positive feedback
Taking too long

## Slide 34
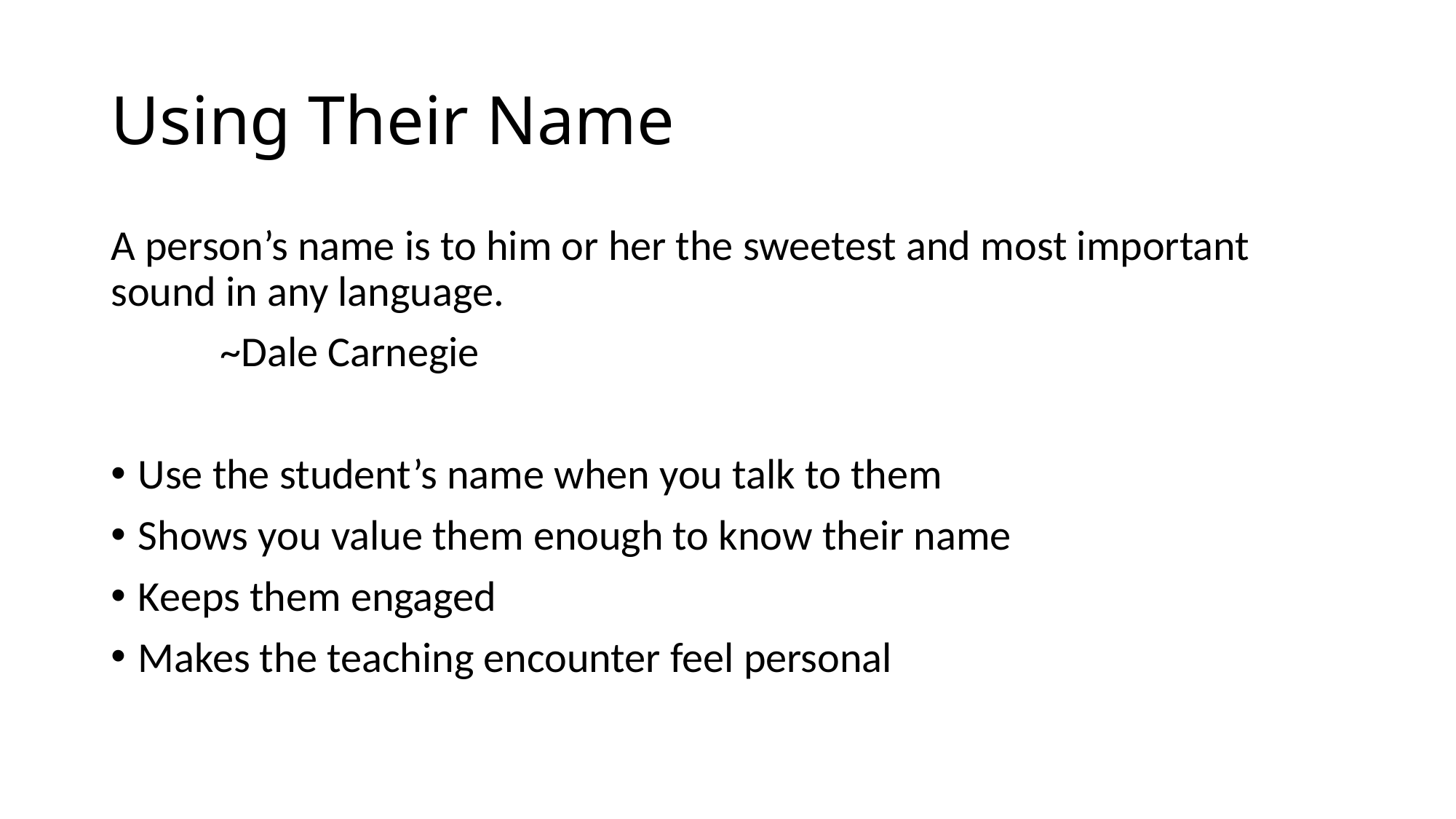

# Using Their Name
A person’s name is to him or her the sweetest and most important sound in any language.
	~Dale Carnegie
Use the student’s name when you talk to them
Shows you value them enough to know their name
Keeps them engaged
Makes the teaching encounter feel personal

## Slide 35
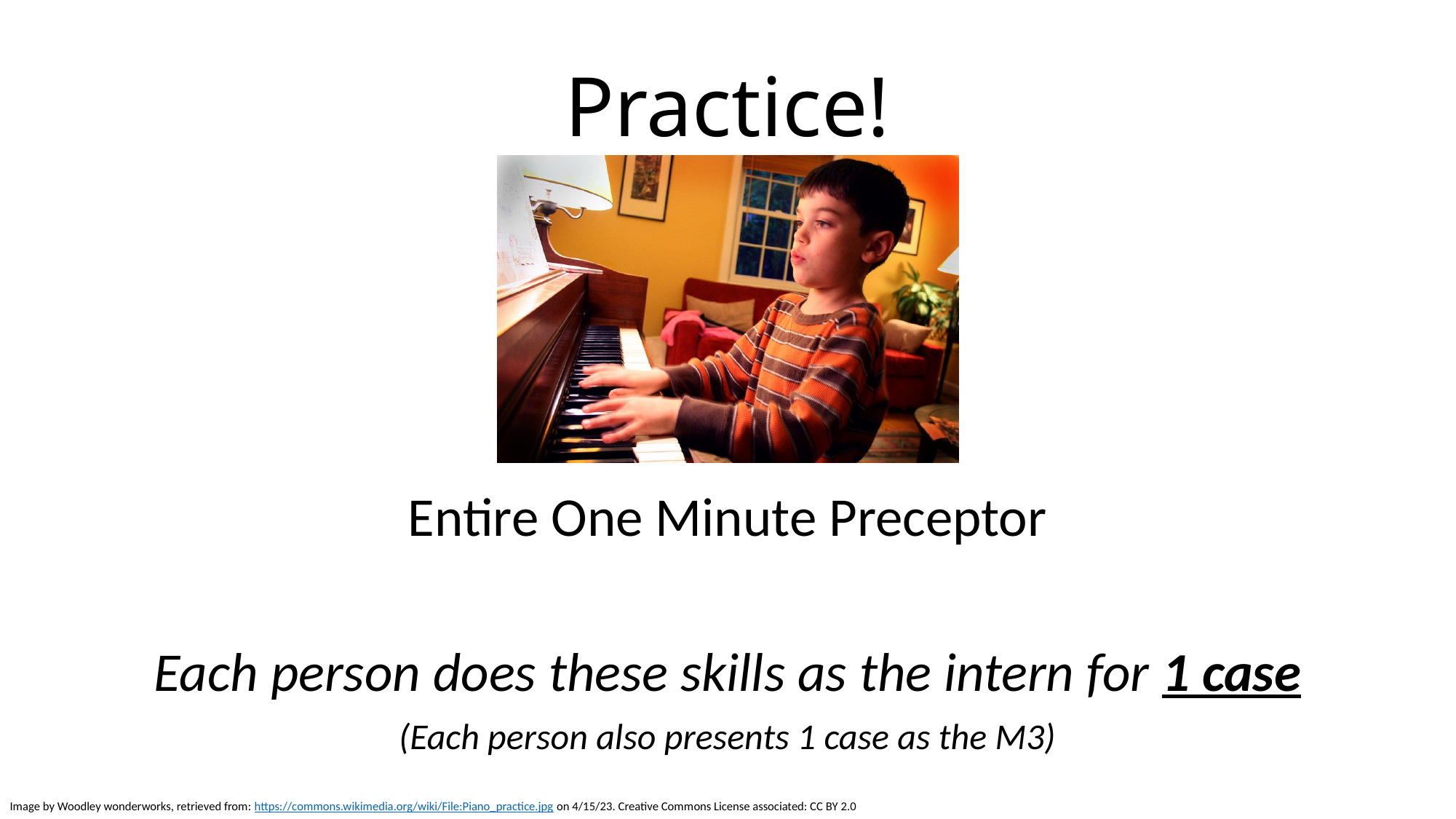

# Practice!
Entire One Minute Preceptor
Each person does these skills as the intern for 1 case
(Each person also presents 1 case as the M3)
Image by Woodley wonderworks, retrieved from: https://commons.wikimedia.org/wiki/File:Piano_practice.jpg on 4/15/23. Creative Commons License associated: CC BY 2.0

## Slide 36
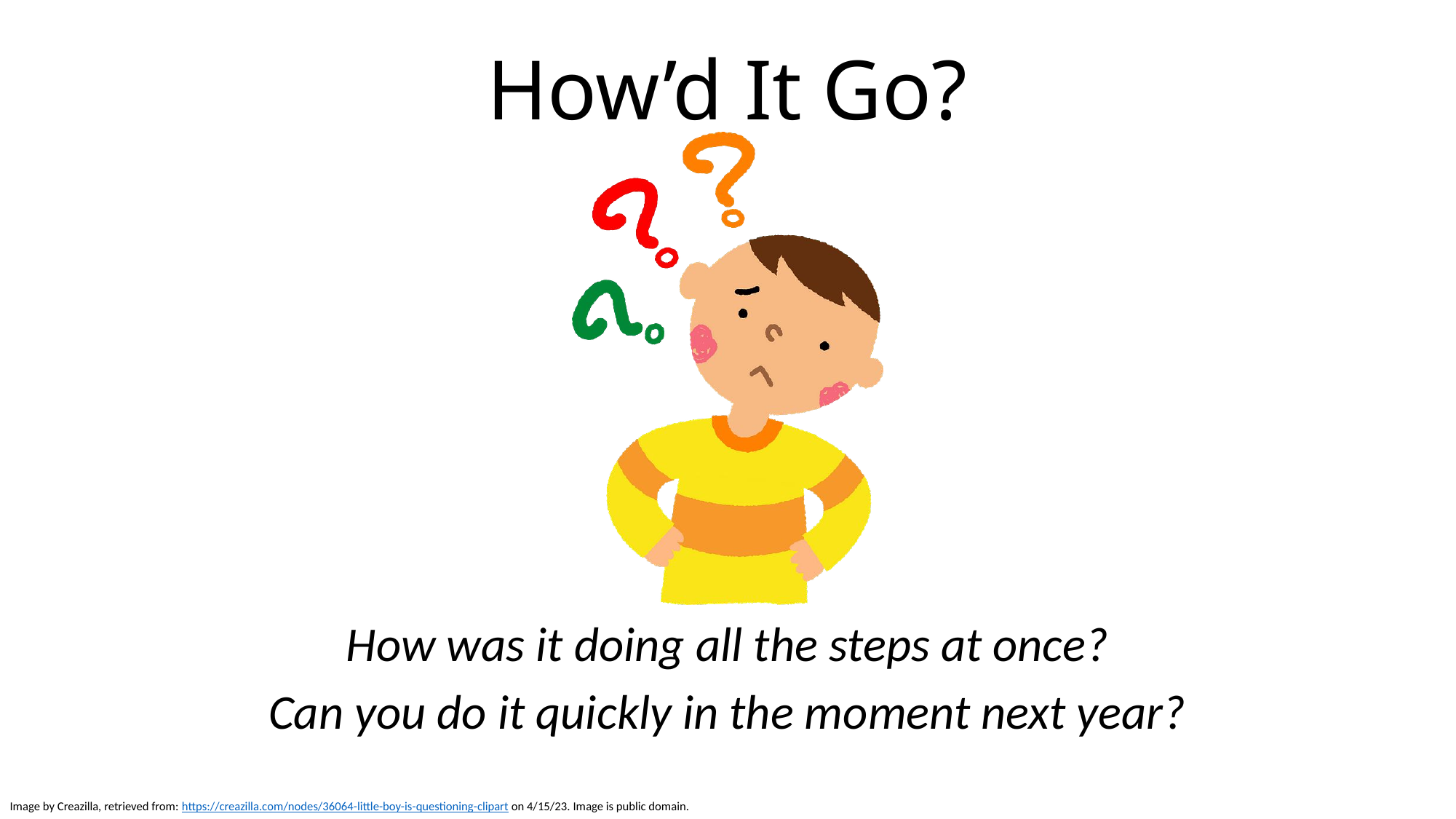

How’d It Go?
How was it doing all the steps at once?
Can you do it quickly in the moment next year?
Image by Creazilla, retrieved from: https://creazilla.com/nodes/36064-little-boy-is-questioning-clipart on 4/15/23. Image is public domain.

## Slide 37
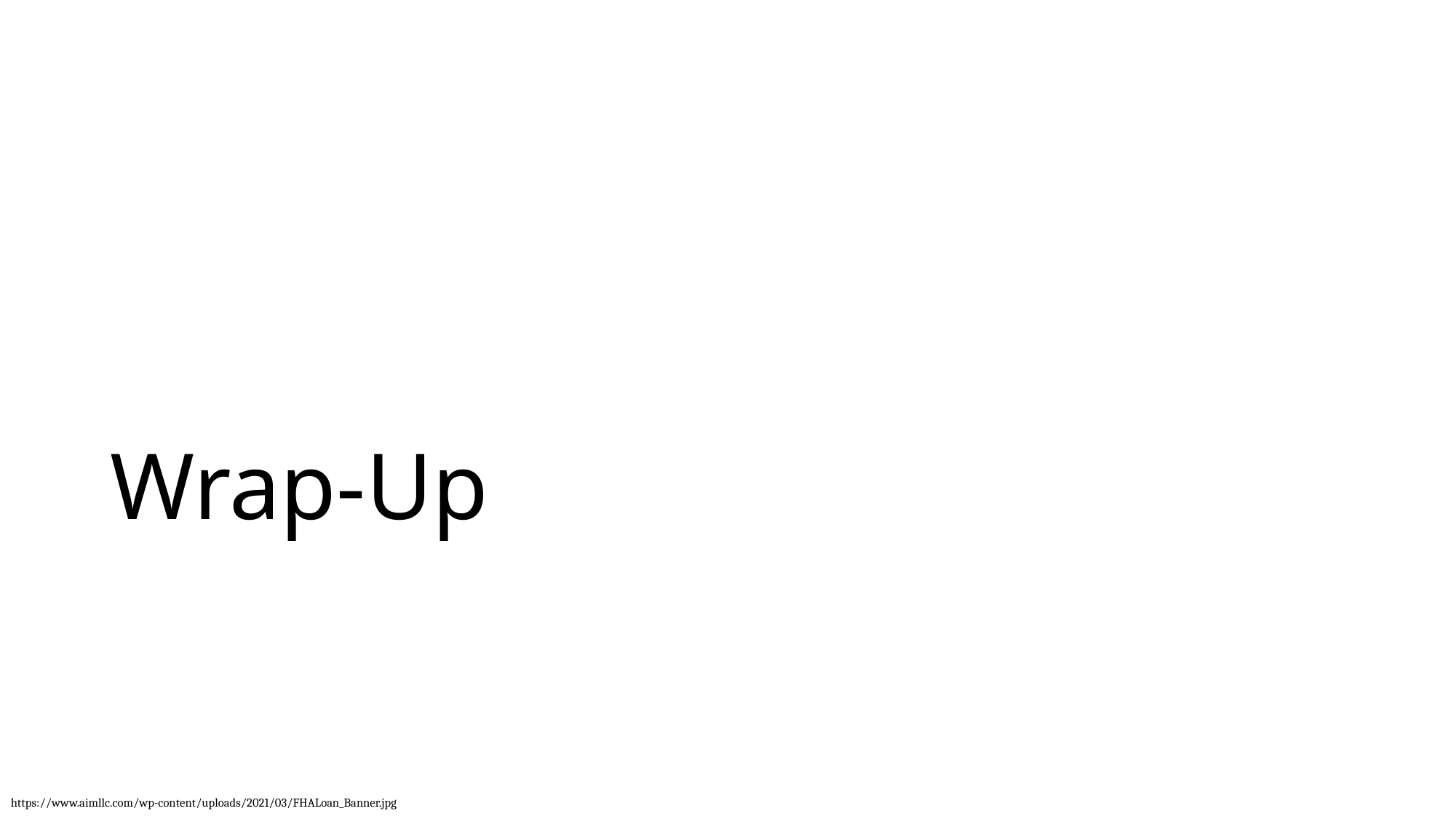

# Wrap-Up
https://www.aimllc.com/wp-content/uploads/2021/03/FHALoan_Banner.jpg

## Slide 38
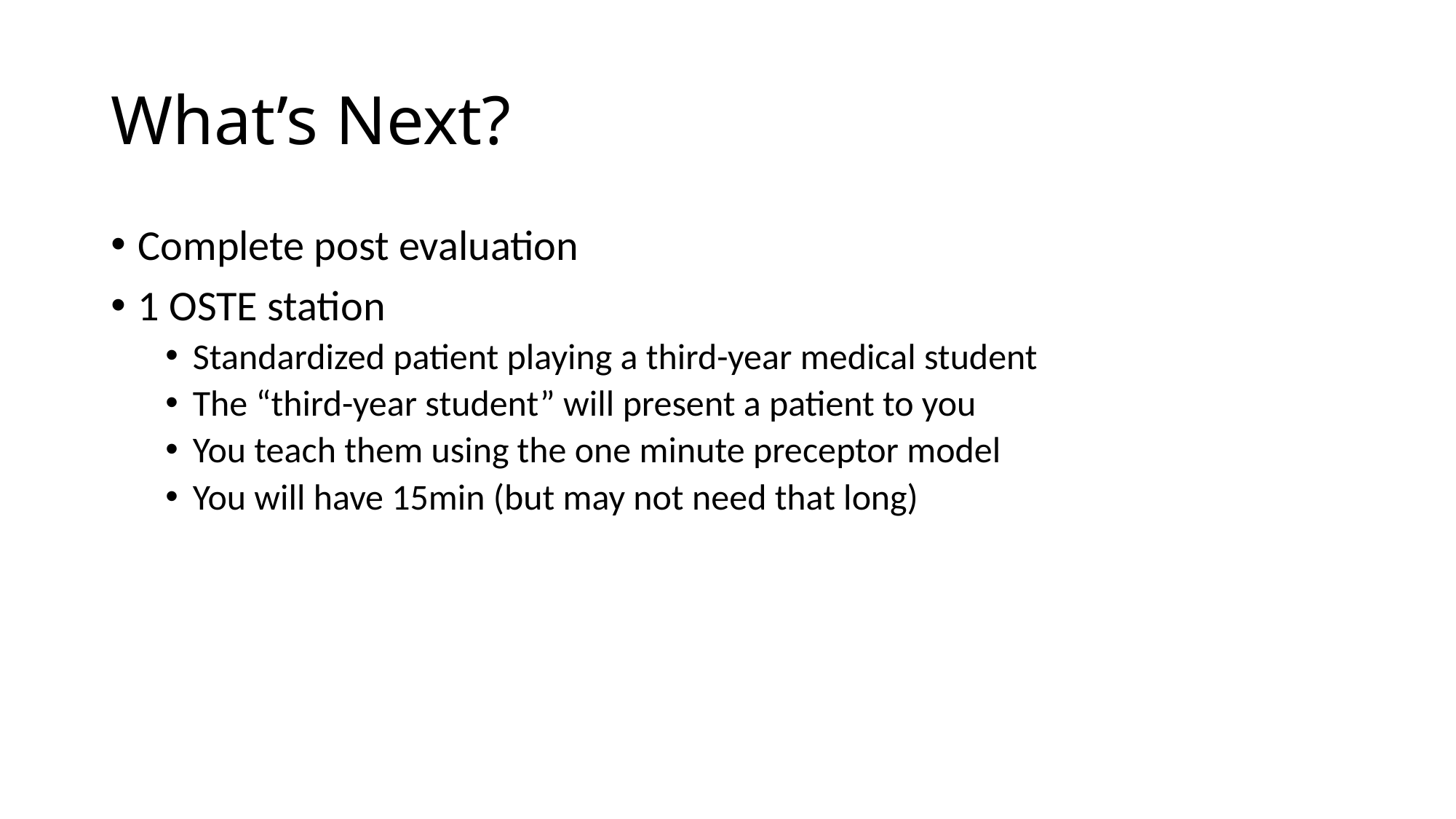

# What’s Next?
Complete post evaluation
1 OSTE station
Standardized patient playing a third-year medical student
The “third-year student” will present a patient to you
You teach them using the one minute preceptor model
You will have 15min (but may not need that long)

## Slide 39
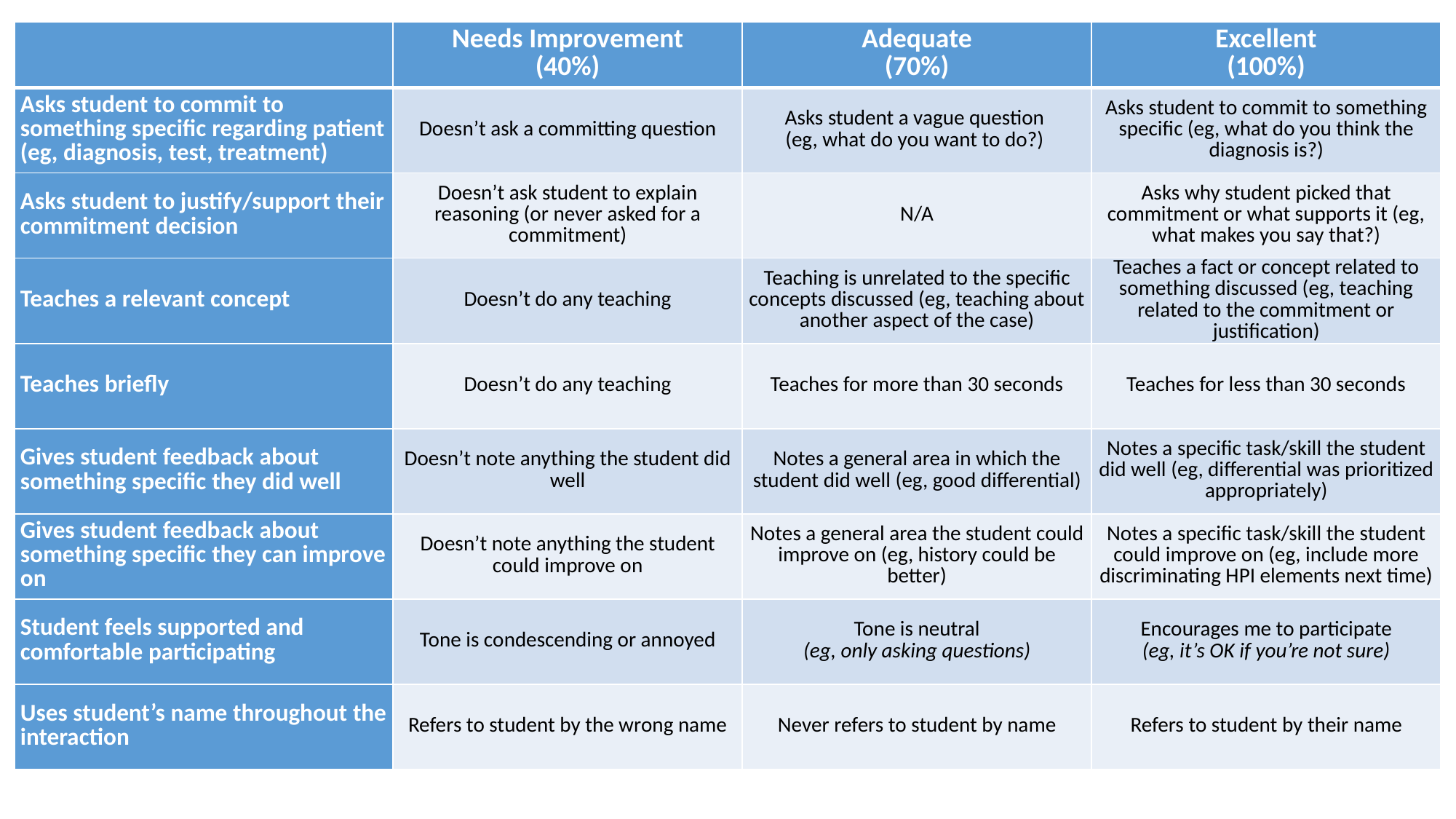

| | Needs Improvement (40%) | Adequate (70%) | Excellent (100%) |
| --- | --- | --- | --- |
| Asks student to commit to something specific regarding patient (eg, diagnosis, test, treatment) | Doesn’t ask a committing question | Asks student a vague question (eg, what do you want to do?) | Asks student to commit to something specific (eg, what do you think the diagnosis is?) |
| Asks student to justify/support their commitment decision | Doesn’t ask student to explain reasoning (or never asked for a commitment) | N/A | Asks why student picked that commitment or what supports it (eg, what makes you say that?) |
| Teaches a relevant concept | Doesn’t do any teaching | Teaching is unrelated to the specific concepts discussed (eg, teaching about another aspect of the case) | Teaches a fact or concept related to something discussed (eg, teaching related to the commitment or justification) |
| Teaches briefly | Doesn’t do any teaching | Teaches for more than 30 seconds | Teaches for less than 30 seconds |
| Gives student feedback about something specific they did well | Doesn’t note anything the student did well | Notes a general area in which the student did well (eg, good differential) | Notes a specific task/skill the student did well (eg, differential was prioritized appropriately) |
| Gives student feedback about something specific they can improve on | Doesn’t note anything the student could improve on | Notes a general area the student could improve on (eg, history could be better) | Notes a specific task/skill the student could improve on (eg, include more discriminating HPI elements next time) |
| Student feels supported and comfortable participating | Tone is condescending or annoyed | Tone is neutral (eg, only asking questions) | Encourages me to participate (eg, it’s OK if you’re not sure) |
| Uses student’s name throughout the interaction | Refers to student by the wrong name | Never refers to student by name | Refers to student by their name |
#
