# Supplementary material for: Preparing Interns as Teachers: Teaching Fourth-Year Medical Students the Tenets of the One-Minute Preceptor Model
Source: MedEdPORTAL. 2023 Dec 26;19:11371. doi: 10.15766/mep_2374-8265.11371 (PMC10749993; doi:10.15766/mep_2374-8265.11371)
Supplement: Supplementary file 1 — Intern-as-Teacher Didactic.pptxCommitment and Justification Cases.docxTeach a General Rule Cases.docxFeedback Cases.docxFull OMP Practice Cases.docxOSTE Case.docxOSTE Rubric.docxPre-Post Evaluation.docxFacilitator Guide.docx [file mep_2374-8265.11371-s001.zip › G. OSTE Rubric.docx]

Intern as Teacher

OSTE Rubric

|  | **Needs Improvement**  (40%) | **Adequate**  (70%) | **Excellent**  (100%) |
| --- | --- | --- | --- |
| **Asks me to commit to something specific regarding patient (e.g., diagnosis, test, treatment)** | Doesn’t ask a committing question | Asks student a vague question *(e.g., what do you want to do?)* | Asks student to commit to something specific *(e.g., what do you think the diagnosis is?)* |
| **Asks me to justify/support my commitment decision** | Doesn’t ask student to explain reasoning (or never asked for a commitment) | N/A | Asks why student picked that commitment or what supports it *(e.g., what makes you say that?)* |
| **Teaches a relevant concept** | Doesn’t do any teaching | Teaching is unrelated to the specific concepts discussed *(e.g., teaching about another aspect of the case)* | Teaches a fact or concept related to something discussed *(e.g., teaching related to the commitment or justification)* |
| **Teaches briefly** | Doesn’t do any teaching | Teaching is >3 sentences in length | Teaching is 1-3 sentences in length |
| **Gives me feedback about something specific I did well** | Doesn’t note anything the student did well | Notes a general area in which the student did well *(e.g., good differential)* | Notes a specific task/skill the student did well *(e.g., differential was prioritized appropriately)* |
| **Gives me feedback about something specific I can improve on** | Doesn’t note anything the student could improve on | Notes a general area the student could improve on *(e.g., history could be better)* | Notes a specific task/skill the student could improve on *(e.g., include more discriminating HPI elements next time)* |
