# Supplementary material for: Preparing Interns as Teachers: Teaching Fourth-Year Medical Students the Tenets of the One-Minute Preceptor Model
Source: MedEdPORTAL. 2023 Dec 26;19:11371. doi: 10.15766/mep_2374-8265.11371 (PMC10749993; doi:10.15766/mep_2374-8265.11371)
Supplement: Supplementary file 1 — Intern-as-Teacher Didactic.pptxCommitment and Justification Cases.docxTeach a General Rule Cases.docxFeedback Cases.docxFull OMP Practice Cases.docxOSTE Case.docxOSTE Rubric.docxPre-Post Evaluation.docxFacilitator Guide.docx [file mep_2374-8265.11371-s001.zip › _Educational Summary Report_11371.pdf]

MedEdPORTAL® The AAMC Journal of Teaching and Learning Resources

Original Publication

Open Access

Preparing Interns as Teachers: Teaching Fourth-Year Medical Students the Tenets of the One-Minute Preceptor Model

Sarah Vick, MD*, John Ragsdale, MD, MS

*Corresponding author: sarah.vick@uky.edu

Abstract

Introduction: Often, interns are expected to teach medical students early in their residency, but most are not formally taught how to be effective teachers before residency. Currently, there is emphasis on developing teaching skills of residents rather than students before they become residents. Most published student-as-teacher courses are voluntary and do not assess skill acquisition. **Methods:** We taught 290 fourth-year medical students across two academic years (2020-2022) the tenets of the One-Minute Preceptor (OMP) using a 2-hour workshop during their transition to residency course. A variety of role-play cases allowed students to practice the different parts of the OMP in isolation and combined. Then, we assessed their teaching skills after the workshop using an objective structured teaching exam (OSTE). **Results:** Two hundred seventy-eight students (96%) completed the self-assessment of their confidence demonstrating the skills of the OMP before and after the workshop. Their confidence improved in all domains, with *p*s $< .001$. Additionally, all students successfully demonstrated competency on the OSTE. **Discussion:** We used a 2-hour workshop based on the OMP to improve fourth-year medical students' confidence in their teaching skills and allow them to demonstrate competence in those skills before starting their intern year.

Keywords
Intern as Teacher, One-Minute Preceptor, Clinical Skills Assessment/OSCEs, Clinical Teaching/Bedside Teaching, Mentoring/Coaching

Educational Objectives

By the end of this activity, learners will be able to:

1. List the steps in the One-Minute Preceptor model.
2. Ask questions of a student to get a clinical commitment and justification.
3. Provide specific, balanced feedback to a student.
4. Provide a brief teaching point to a student.

Introduction

Interns across specialties are often expected to teach medical students as soon as they begin residency, but they are often not taught skills on how to teach effectively before starting their intern year. Currently, most teaching programs are designed to focus on resident or faculty teaching skills, and there are few programs focused on enhancing the teaching skills of fourth-year medical students. In a recent review of student-as-teacher curricula, most were optional, and few were linked to observation of teaching skills or feedback.[1] Our resource focused on teaching fourth-year medical students the One-Minute Preceptor (OMP) model to prepare them for teaching from the first day of their intern year.

Citation:
Vick S, Ragsdale J. Preparing interns as teachers: teaching fourth-year medical students the tenets of the One-Minute Preceptor model. *MedEdPORTAL*. 2023;19:11371.
https://doi.org/10.15766/mep_2374-8265.11371

Many medical schools utilize a transition to residency (TTR) course that teaches critical intern skills to fourth-year students. These courses often focus on discrete clinical skills such as answering pages, postacute care, handoff, end-of-life care, and so on.[2-7] As these courses have grown nationally, a TTR Compendium began in 2023 to house TTR resources used in different courses.[8] As with the other TTR content currently published, the compendium focuses on clinical skills like cross-cover, informed consent, and delivering serious news. However, currently, there is no readily available workshop on developing the teaching skills of fourth-year students during these courses, even though teaching medical students is a common role for interns. We identified this gap in our TTR course at the University of Kentucky and sought to provide our graduating students with a teaching construct using the OMP.

The OMP is a frequently used modality for improving teaching skills. A systematic review of various teaching methodologies

Copyright © 2023 Vick and Ragsdale. This is an open-access publication distributed under the terms of the Creative Commons Attribution license.

showed that use of the OMP demonstrated a significant improvement in faculty teaching behaviors.[9] Additionally, a randomized controlled trial recommended the use of the OMP as the most effective way to improve teaching behaviors in residents.[10] This randomized controlled trial also recommended the use of an objective structured teaching exam (OSTE) to assess behavior changes related to the teaching intervention.[10] Due to the evidence of the OMP's effectiveness, we chose to focus our workshop on this modality.

Several workshops and materials are available for teaching residents to be more effective teachers.[11,12] However, many residents already have some experience teaching and supervising students before these materials are provided to them. The OMP is used in many of these resident-as-teacher programs. One program focuses directly on teaching the OMP as the way to help residents become more effective teachers.[13] Other programs use the OMP as one part of a larger resident teaching curriculum.[14,15] Another program targets senior residents rather than interns since they have more responsibility in teaching the entire team.[16] However, all of these interventions occur after the beginning of their residency programs, rather than preparing new interns before they begin.

There are many published workshops and teaching series for faculty development of teaching skills. Some focus on teaching the OMP to faculty to use when working with students and residents.[17] Other programs use the OMP as a part of a larger way to teach different levels of learners or train faculty in different teaching skills.[18,19] Some faculty development programs use role-play to develop teaching skills, though not all use the OMP.[20,21] Overall, faculty development for teaching is often based on the OMP model.

Regardless of the target audience, one method for assessing the effectiveness of teaching training programs is an OSTE. Several interventions and OSTEs for residents and faculty are available that focus on providing objective assessment of teaching skills.[10,22-26] These programs assess a variety of teaching skills in an objective way to provide assessment and feedback to residents and faculty on their teaching skills. Some OSTEs focus on feedback[24] and others on clinical teaching skills.[22,25,26] A few include the OMP as part of a larger teaching program for residents.[23] No students are assessed in these published OSTEs.

Methods

Workshop Overview

Our intern-as-teacher workshop was part of a required capstone TTR course for fourth-year medical students. This 4-week course taught and assessed a variety of skills needed for the successful transition to internship through didactics, workshops, simulation, and OSCEs. Every fourth-year medical student at our institution took this course in April of their fourth year across all campuses. The instructional method for our innovation was a 2-hour, faculty-facilitated workshop teaching the tenets of the OMP model using role-play. Knowing that the majority of our graduates would go on to residencies where teaching would be part of their daily practice, we felt it was important to provide them with teaching skills they could use from the start of their intern year. The OMP model was one of the most common methods taught, and breaking the skills apart into sections with practice allowed students to master each skill before using them all together.

The overall workshop size was 30-45 fourth-year students, who were organized into sessions based on specialty of interest (adult medicine, surgical specialties, or pediatrics). We had those going into fields like OB/GYN, psychiatry, and ear, nose, and throat (ENT) choose one of the three tracks that aligned best with their future goals. For the 2022-2023 academic year (outside the scope of the results reported here), we added additional cases in OB/GYN, ENT, anesthesia, and orthopedics in order to provide more specific questions for these groups, and we plan to continue to add specialty-specific cases in the future. We created these different groups so that the practice cases students used would be linked to their specialty of choice to increase student buy-in and transference of the skills.

Workshop Organization

The organization of the workshop was based on the five microskills of the OMP,[27] explained in a PowerPoint presentation (Appendix A). We first had the third-year students reflect on their own experiences of being taught and then introduced them to published data about the OMP. We started with reflection to encourage participation early in the workshop and to prime the students to think about effective and less effective teachers they had experienced. We grouped the five microskills into three segments: (1) get a commitment and probe for justification, (2) teach a general rule, and (3) provide reinforcing and constructive feedback. These segments were logical breaks in the microskills that facilitated scaffolding and practice. For each segment, we taught the skills using the PowerPoint presentation, with the students role-playing each skill in small groups of four to five of their peers. One student would play a third-year student who was being taught, and a different student would play the intern who was practicing the OMP skills. Before our workshop, participants' familiarity with each skill of the OMP varied. Therefore, we intentionally separated the skills into these segments, with dedicated practice for each, so that students could hone each

Copyright © 2023 Vick and Ragsdale. This is an open-access publication distributed under the terms of the Creative Commons Attribution license.

skill individually before being asked to put them all together. After intentional practice, we had students put all the microskills together for a final role-play using the entire OMP model at the end of the workshop.

Workshop by Section

The first section was the "get a commitment and probe for justification" segment in which the intern asked for some type of commitment from the third-year and then asked a question about why the third-year had chosen that answer. For this section, we reviewed the skills and provided multiple examples. Then, we addressed common pitfalls related to getting commitments and justifications from students. Students took turns role-playing, with the third-year reading a case to the intern (Appendix B). The case included history, exam, and labs but lacked any diagnosis or assessment so that the intern would be forced to get a commitment from the third-year and probe for justification. Several examples of possible questions and justifications were provided with each case to ensure the third-year could answer any questions the intern asked. Each participant played the intern once and the third-year once.

The second section was the "teach a general rule" segment in which the intern taught a brief concept related to the case. In the presentation, we reviewed the skill and provided examples with common pitfalls related to brief teaching. For the role-play, students drew a slip of paper with a brief case description and a diagnosis (Appendix C). Playing the intern, they read their case aloud and then provided their own general teaching rule related to that case to the rest of their group. The goal was to keep teaching brief, ideally less than 90 seconds. Each participant completed two cases, providing a brief teaching point for each.

The third section was the "provide reinforcing and constructive feedback" segment in which the intern provided feedback to the third-year. We reviewed the skills for providing feedback and showed examples with common pitfalls related to giving feedback. For the role-play, the third-year read a clinical case presentation to the intern (Appendix D). The cases were written to provide a variety of potential feedback, including opportunities for reinforcing and constructive feedback in each case. After listening to the presentation, the intern provided at least one point of reinforcing feedback and one point of constructive feedback. Each participant played the intern once and the third-year once.

In the last section of the workshop, students incorporated all five of the microskills (from all three segments) to perform the entire OMP model of teaching. The third-year read a case presentation to the intern (Appendix E). The intern then practiced all OMP skills together with the third-year. Each participant played the intern once and the third-year once.

OSTE Assessment

To assess competency in teaching, all students completed an OSTE applying what they had learned to a novel case (Appendix F) after the workshop. The standardized patient (SP) in the OSTE played the role of a third-year medical student and read a script of a typical case presentation on a new patient admitted with pneumonia. The actual fourth-year student played the role of the intern and taught the SP using the OMP model. The SP evaluated the student after the encounter using a rubric based on the educational objectives for the session (Appendix G). The SP was responsible for reading the script and grading the teaching (not the medicine). The role of the SP could be played by a faculty member or staff person familiar with the rubric if SPs were not readily available.

We developed the case and the rubric for this assessment. The OSTE case mirrored the practice cases used throughout the workshop. The case provided chances for the student to ask for a commitment and justification, but it also had some opportunities for improvement so that there was ample material for feedback. We designed the rubric to reflect the individual tenets of the OMP to ensure that every student mastered each component of the model. We provided specific examples in the rubric to help the SPs differentiate between the different levels. Additionally, as discussed in the Training and Standardization section below, we conducted a training session with the SPs prior to the OSTE to ensure reliability and validity of their assessments.

We chose to feature a patient with pneumonia because it is a common condition that all students are exposed to during medical school and would have some knowledge about it available when using the OMP model to teach. Nearly all specialties encounter pneumonia in some manner, making it applicable to the entire group. Additionally, since teaching was the skill we were assessing, we felt that one case was sufficient. In the 2022-2023 academic year (not included in these data), we did create cases for surgery and pediatrics for a more specialty-specific assessment.

Student Self-Assessment

As another measure of the effectiveness of our resource, we surveyed the students about their perception of their teaching skills before and after the workshop. Students filled out a pre/post survey (Appendix H) rating their confidence in their teaching skills before and after the workshop. Participants

Copyright © 2023 Vick and Ragsdale. This is an open-access publication distributed under the terms of the Creative Commons Attribution license.

rated their confidence level on a 5-point Likert scale (1 = *not at all confident,* 5 = *extremely confident*). The questions on the survey mirrored our educational objectives for the session. The pre/post results were linked by a number in the top right corner of the surveys but were otherwise anonymous. Since this project involved medical students, we obtained IRB exemption (University of Kentucky, 84301, February 16, 2023).

Workshop Materials

The workshop took place in a large classroom with tables so that the 35-40 students per session could spread out comfortably into seven or eight groups of four to five students. The room had a large central screen for projecting the PowerPoint presentation. All materials were printed before the workshop and grouped by section of the workshop. For the "get a commitment and probe for justification," "provide reinforcing and constructive feedback," and final "putting it all together" sections, eight copies of each case were printed so that each student in a given group received a different case. For the "teach a general rule" section, eight copies of the cases were printed (one for each group), and then the cases were cut into individual slips of paper so that students could draw a single case and teach about it. We passed out the cases for a section when it was time to practice that section to make sure the correct cases were being used each time.

Training and Standardization

Our institution has four campuses. We created a facilitator guide to ensure appropriate timing of the workshop across all campuses (Appendix I). We trained faculty facilitators during a 1-hour session where we walked them through the PowerPoint presentation, the cases for each section, and the OSTE. For the OSTE, we reviewed and practiced the case with the SPs. One of the faculty facilitators played the role of the intern in the case to provide feedback to the SPs on their portrayal of the third-year student. The SPs filled out the rubric based on the faculty member's performance, and we provided feedback on their grading to ensure that all SPs portrayed the case consistently. This session was electronically transmitted to our regional campuses and recorded, so all SPs received the same training.

Analysis

After the session, we paired the surveys by number to analyze the difference between the student ratings before and after the workshop. We analyzed the ratings using a paired *t* test with a two-tailed alpha of .05. We analyzed the OSTE results using means and proportions.

Results

Over the two academic years of 2020-2021 and 2021-2022, 290 fourth-year medical students completed the workshop. We had pre/post survey data available for 278 students (96%) for analysis. Nine of the surveys had no data when submitted, and three were incomplete (missing either a pre- or posttest). Student confidence in all six domains improved by at least 0.9 out of 5.0, with $p < .001$ (Table). The greatest increase was in eliciting a commitment (1.2 increase in mean), and the smallest increase was in providing a brief teaching point (0.9 increase in mean).

All 290 students were evaluated using an OSTE case. One hundred percent of students passed the OSTE, with a mean score of 96% of the rubric items completed correctly. We acknowledge that this pass rate is very high. We anticipate that it is likely due to the rubric sections following the specific practice components of the workshop. Additionally, unless a student did not perform a specific skill, it would be hard to fail this OSTE. Our goal was to ensure that students learned the skills and felt confident in teaching when they started their intern year. We are encouraged by the high passing rate, as it makes us feel that our graduates will have these teaching skills going into their intern year. We hope that these skills would be retained after graduation, but we did not conduct a follow-up survey of our graduates to determine if they were retained or regularly used.

Discussion

Our workshop innovates by using the OMP to develop the teaching skills of fourth-year students before they begin their intern year. Other workshops and series using the OMP published to date develop faculty and resident teaching skills, but many interns teach earlier than that. It is important to equip fourth-year students with a tool in their teaching toolbox before they arrive in residency. We believe our workshop was effective because students reported pre- and postimprovement in skills (Kirkpatrick level 1) and passed an OSTE based on performance of these skills (Kirkpatrick level 2).[28]

This workshop has evolved in several ways based on course evaluations and facilitator reflection. First, we found that students were eager to perform the entire OMP from the start. We had to intentionally remind them at the practice time for each section to focus only on the skill(s) for that section so

Table. Pre/Post Survey Data ($N = 278$)

Item[a]	Pre *M*	Post *M*	*p*
Teach students as intern	2.7	3.8	<.001
Elicit commitment	3.1	4.3	<.001
Probe for justification	3.1	4.2	<.001
Affirming feedback	3.1	4.1	<.001
Constructive feedback	2.7	3.8	<.001
Brief teaching point	2.9	3.8	<.001

[a]Rated on a 5-point Likert scale (1 = *not at all confident,* 5 = *extremely confident*).

Copyright © 2023 Vick and Ragsdale. This is an open-access publication distributed under the terms of the Creative Commons Attribution license.

they could intentionally practice each skill through scaffolding. Additionally, we found students were more eager to participate in the workshop after we invited them at the start to reflect on their own experiences of having been taught. We believe that this priming enhanced their engagement during our workshop.

In addition, prior to the cohorts presented here (in an earlier version of the workshop), we used SPs to play the third-year students, as we were worried about participant engagement in role-play. We provided the SPs with extensive scripts about what possible medical decision-making questions they might have to answer, as they would not have the medical background to answer some questions asked in the natural flow of the workshop. However, we found that even with answers to potential questions provided, the SPs' lack of medical background made the activity feel less authentic to the students. Additionally, students reported in course evaluations that they would have preferred role-play rather than using SPs. The next year, we changed to role-play rather than adding the additional resource for SPs. This change was well received by the students and allowed the cases to feel more like a real encounter.

An important strength of this resource is that it includes an OSTE objectively assessing the skills acquired by students. With an average score of 96%, students were able to master these skills during our 2-hour workshop. Another strength is the intentional use of scaffolding, in which students practice each microskill before putting them all together as an entire model. Based on our large cohort of nearly 300 students, each objective was met according to the student ratings. Additionally, having practice cases tracked to our students' specialty of interest increased their buy-in during the workshop and its applicability to their future careers. Another important strength of this resource is generalizability. Given that the workshop has been offered at four campuses by different facilitators, we believe that it can be implemented by other institutions using the same materials.

One limitation of the workshop is the time required to deliver it. While 2 hours is not a large investment within the context of an entire TTR course, for institutions without such a course there may not be a structure in which to deliver the workshop. Other challenges include availability of SPs to conduct the OSTE and faculty time to train the SPs. Lastly, we did not have a control group, so it is unknown how students would have performed on the OSTE without this workshop.

In summary, teaching the OMP model to fourth-year students was well received and enhanced self-assessed confidence in teaching skills and demonstrated competence on an OSTE. This resource focuses on the most common teaching model, the OMP, providing graduating students with a tool for teaching on day one of their intern year.

Appendices

A. Intern-as-Teacher Didactic.pptx
B. Commitment and Justification Cases.docx
C. Teach a General Rule Cases.docx
D. Feedback Cases.docx
E. Full OMP Practice Cases.docx
F. OSTE Case.docx
G. OSTE Rubric.docx
H. Pre-Post Evaluation.docx
I. Facilitator Guide.docx

All appendices are peer reviewed as integral parts of the Original Publication.

Sarah Vick, MD: Assistant Professor, Department of Internal Medicine, University of Kentucky College of Medicine; ORCID: https://orcid.org/0009-0001-2197-8759

John Ragsdale, MD, MS: Associate Professor, Department of Internal Medicine, University of Kentucky College of Medicine; ORCID: https://orcid.org/0000-0002-9942-1192

Disclosures
None to report.

Funding/Support
None to report.

Ethical Approval
The University of Kentucky Institutional Review Board deemed further review of this project not necessary.

References

1. Cohen A, Steinert Y, Ruano Cea E. Teaching medical students to teach: a narrative review and literature-informed recommendations for student-as-teacher curricula. *Acad Med*. 2022;97(6):909-922. https://doi.org/10.1097/ACM.0000000000004608
2. Cetrone E, Mutter K, Pedersen K, Shah N, Martindale J. A paging training program for a fourth-year internship readiness course. *MedEdPORTAL*. 2020;16:11021. https://doi.org/10.15766/mep_2374-8265.11021

Copyright © 2023 Vick and Ragsdale. This is an open-access publication distributed under the terms of the Creative Commons Attribution license.

3. Lescinskas E, Sargsyan Z, Ayyala US, Fisher J. Preparing for medical internship: a case-based strategy to teach management of common overnight calls to students. *MedEdPORTAL*. 2020; 16:10966. https://doi.org/10.15766/mep_2374-8265.10966
4. Tischendorf J, O'Connor C, Alvarez M, Johnson S. Mock paging and consult curriculum to prepare fourth-year medical students for medical internship. *MedEdPORTAL*. 2018;14:10708. https://doi.org/10.15766/mep_2374-8265.10708
5. Nonaillada J. Rehab concepts and discharge dispositions: workshop for medical students. *MedEdPORTAL*. 2018;14:10785. https://doi.org/10.15766/mep_2374-8265.10785
6. Chen T, Stapleton S, Babcock M, Kelley MN, Frallicciardi A. Handoffs and nurse calls: overnight call simulation for fourth-year medical students. *MedEdPORTAL*. 2021;17:11138. https://doi.org/10.15766/mep_2374-8265.11138
7. Jeffers JM, Bord S, Hooper JE, Fleishman C, Cayea D, Garibaldi B. End-of-life care: a multimodal and comprehensive curriculum for graduating medical students utilizing experiential learning opportunities. *MedEdPORTAL*. 2021;17:11149. https://doi.org/10.15766/mep_2374-8265.11149
8. TTR Compendium. Transition to Residency (TTR) Course Educators. Accessed November 8, 2023. https://www.ttreducators.com/compendium
9. Furney SL, Orsini AN, Orsetti KE, Stern DT, Gruppen LD, Irby DM. Teaching the One-Minute Preceptor: a randomized controlled trial. *J Gen Intern Med*. 2001;16(9):620-624. https://doi.org/10.1046/j.1525-1497.2001.016009620.x
10. Post RE, Quattlebaum RG, Benich JJ III. Residents-as-teachers curricula: a critical review. *Acad Med*. 2009;84(3):374-380. https://doi.org/10.1097/ACM.0b013e3181971ffe
11. Newman L, Tibbles CD, Atkins KM, et al. Resident-as-teacher DVD series. *MedEdPORTAL*. 2015;11:10152. https://doi.org/10.15766/mep_2374-8265.10152
12. Harrell H, Wipf J, Aronowitz P, et al. Resident as teacher curriculum. *MedEdPORTAL*. 2015;11:10001. https://doi.org/10.15766/mep_2374-8265.10001
13. Bensinger LD, Meah YS, Smith LG. Resident as teacher: the Mount Sinai experience and a review of the literature. *Mt Sinai J Med*. 2005;72(5):307-311.
14. Hayes JR, Zeman J, Johnston B, Campbell N. Outpatient teaching and feedback skills workshop for resident physicians. *MedEdPORTAL*. 2020;16:10930. https://doi.org/10.15766/mep_2374-8265.10930
15. Palamara K, Ray A. Resident-as-teacher workshop. *MedEdPORTAL*. 2014;10:9673. https://doi.org/10.15766/mep_2374-8265.9673
16. Hilburg R, Coyle A. Resident as preceptor: an ambulatory internal medicine curriculum for third-year resident precepting. *MedEdPORTAL*. 2020;16:11000. https://doi.org/10.15766/mep_2374-8265.11000
17. Farrell L, DeWolfe C, Cuzzi S, Ismail L, Newman D, Kalburgi S. Strategies for teaching medical students: a faculty development workshop for pediatric preceptors in the community setting. *MedEdPORTAL*. 2020;16:10890. https://doi.org/10.15766/mep_2374-8265.10890
18. Khidir A, Alhammadi A, Wagdy M, Mian M. Sharing the light: teaching different levels of learners. *MedEdPORTAL*. 2016;12: 10388. https://doi.org/10.15766/mep_2374-8265.10388
19. Loyal J, Porto A, Camenga D. Creating a program for junior faculty professional development: a tool kit. *MedEdPORTAL*. 2018;14:10703. https://doi.org/10.15766/mep_2374-8265.10703
20. Servey J, Wyrick K. Teaching clinical precepting: a faculty development workshop using role-play. *MedEdPORTAL*. 2018; 14:10718. https://doi.org/10.15766/mep_2374-8265.10718
21. Oller D. The ambulatory teaching minute: development of brief, case-based, evidence-based medicine exercises for the internal medicine resident continuity clinic. *MedEdPORTAL*. 2020;16: 10909. https://doi.org/10.15766/mep_2374-8265.10909
22. Ricciotti HA. Implementing an objective structured teaching exercise (OSTE) in an OBGYN residency program. *MedEdPORTAL*. 2013;9:9425. https://doi.org/10.15766/mep_2374-8265.9425
23. Scott D, Cook S, DuBeau C, Podrazik P. A four-station OSTE (objective structured teaching exercise) in geriatric medicine. *MedEdPORTAL*. 2013;9:9574. https://doi.org/10.15766/mep_2374-8265.9574
24. Smith J. An objective structured teaching exercise (OSTE) for physicians employing multi-source feedback. *MedEdPORTAL*. 2015;11:10157. https://doi.org/10.15766/mep_2374-8265.10157
25. Zackoff M, Jerardi K, Unaka N, Fleming A, Patterson B, Klein M. Resident-as-teacher curriculum and assessment tool for brief didactic teaching in pediatrics. *MedEdPORTAL*. 2015;11:10030. https://doi.org/10.15766/mep_2374-8265.10030
26. Morrison E. An objective structured teaching examination (OSTE) for generalist resident physicians. *MedEdPORTAL*. 2005;1:103. https://doi.org/10.15766/mep_2374-8265.103
27. Neher JO, Gordon KC, Meyer B, Stevens N. A five-step "microskills" model of clinical teaching. *J Am Board Fam Pract*. 1992;5(4):419-424.
28. Kirkpatrick JD, Kirkpatrick WK. *Kirkpatrick's Four Levels of Training Evaluation*. ATD Press; 2016.

Received: May 17, 2023
Accepted: October 5, 2023
Published: December 26, 2023

Copyright © 2023 Vick and Ragsdale. This is an open-access publication distributed under the terms of the Creative Commons Attribution license.
